# Synthesis of bis-spirocyclic derivatives of 3-azabicyclo[3.1.0]hexane via cyclopropene cycloadditions to the stable azomethine ylide derived from Ruhemann's purple

**DOI:** 10.3762/bjoc.18.77

**Published:** 2022-06-29

**Authors:** Alexander S Filatov, Olesya V Khoroshilova, Anna G Larina, Vitali M Boitsov, Alexander V Stepakov

**Affiliations:** 1 Saint-Petersburg State University, Universitetskaya nab. 7/9, 199034, St. Petersburg, Russian Federation; 2 Saint-Petersburg National Research Academic University of the Russian Academy of Sciences, ul. Khlopina 8/3, 194021, St. Petersburg, Russian Federation; 3 Saint-Petersburg State Institute of Technology, Moskovskii pr. 26, 190013, St. Petersburg, Russian Federation

**Keywords:** azomethine ylides, cycloaddition, cyclopropenes, DFT calculations, spiro heterocycles

## Abstract

A reliable method for the synthesis of bis-spirocyclic derivatives of 3-azabicyclo[3.1.0]hexanes through the 1,3-dipolar cycloaddition (1,3-DC) reactions of cyclopropenes to the stable azomethine ylide – protonated form of Ruhemann's purple (PRP) has been developed. Both 3-substituted and 3,3-disubstituted cyclopropenes reacted with PRP, affording the corresponding bis-spirocyclic 3-azabicyclo[3.1.0]hexane cycloadducts in moderate to good yields with high diastereofacial selectivity. Moreover, several unstable 1,2-disubstituted cyclopropenes were successfully trapped by the stable 1,3-dipole under mild conditions. The mechanism of the cycloaddition reactions of cyclopropenes with PRP has been thoroughly studied using density functional theory (DFT) methods at the M11/cc-pVDZ level of theory. The cycloaddition reactions have been found to be HOMO_cyclopropene_–LUMO_ylide_ controlled while the transition-state energies for the reaction of 3-methyl-3-phenylcyclopropene with PRP are fully consistent with the experimentally observed stereoselectivity.

## Introduction

**Scheme 1:**
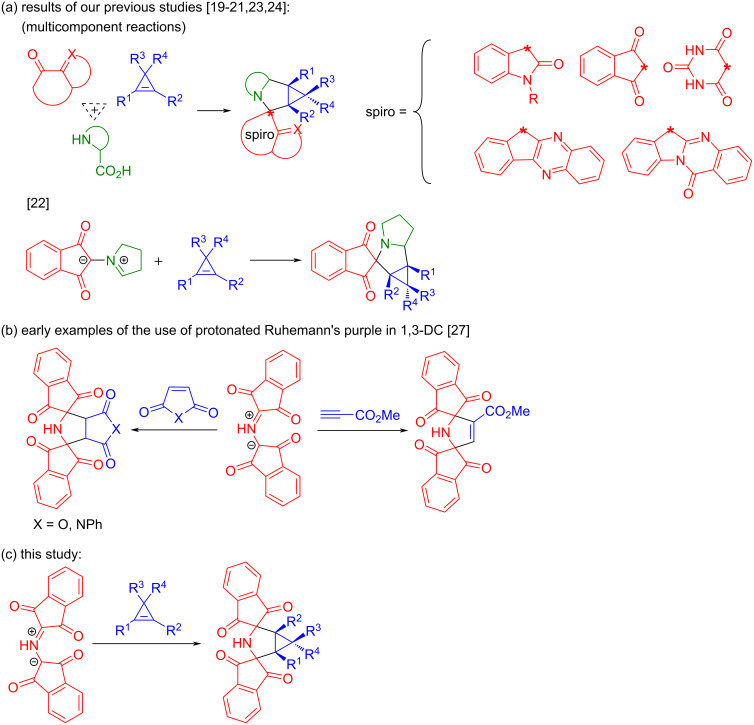
Early studies concerning cyclopropene cycloadditions to azomethine ylides and cycloaddition reactions involving protonated Ruhemann's purple (PRP).

Spiro compounds (molecules containing at least two rings with only one common atom) are an important class of both synthetic and naturally occurring substances. Many biologically active natural products have a spirocyclic skeleton in their structure [1–2]. In this regard, there is interest in studying heterocyclic spiro compounds for drug discovery. These compounds were found to exhibit a broad range of biological activities, including antioxidant [3], anticancer [4], antidiabetic [5], and antibacterial [6] properties. It is also worth noting that spiro compounds have found application in agriculture as fungicides [7], as well as in materials science as organic semiconductors [8]. The 3-azabicyclo[3.1.0]hexane framework is a valuable structural fragment found in natural compounds [9–11]. It is used in pharmaceuticals [12–15] and key intermediates [16–17]. Compounds containing a 3-azabicyclo[3.1.0]hexane moiety are antagonists of morphine-induced antinociception [18], histone deacetylase inhibitors [13], and opioid receptor antagonists [15]. In our recent studies, great attention was paid to developing methods for the synthesis of spiro[3-azabicyclo[3.1.0]hexanes] based on 1,3-dipolar cycloaddition reactions involving azomethine ylides and cyclopropene dipolarophiles, and also the in vitro activity of some synthesized compounds has been explored [19–24]. To generate azomethine ylides, we used a classical method based on the reaction of cyclic carbonyl compounds with α-amino acids. Μono-, bi-, and tetracyclic carbonyl compounds were utilized in these studies. 1,3-Dipolar cycloaddition of cyclopropenes to azomethine ylides generated from primary or secondary α-amino acids and carbonyl compounds such as alloxan, isatin, tryptanthrin, and 11*H*-indeno[1,2-*b*]quinoxalin-11-one were performed in a multicomponent fashion [19–21,24]. In these reactions, the azomethine ylides generated in situ are highly reactive and cannot be isolated as individual compounds. At the same time, the reaction of ninhydrin and proline results in the formation of the stable azomethine ylide. This 1,3-dipole demonstrated high reactivity towards diverse cyclopropenes, including parent cyclopropene [22]. Note that the reactions of cyclopropenes with azomethine ylides from ninhydrin were also successfully carried out in a multicomponent fashion [23]. Mention should also be made of the recent advances in developing enantioselective approaches to the synthesis of 3-azabicyclo[3.1.0]hexane derivatives. Deng and co-workers reported the first asymmetric 1,3-dipolar cycloaddition of azomethine ylides and cyclopropenes catalyzed by a chiral Cu-(CH_3_CN)_4_BF_4_/Ph-Phosferrox complex for the construction of 3-azabicyclo[3.1.0]hexane derivatives [25]. Another concise enantioselective approach towards 3-azabicyclo[3.1.0]hexanes is based on a Cp*Ir-catalyzed reductive amination/cyclization of enantiopure *cis*-cyclopropane dicarbonyls [26]. The strategy based on azomethine ylide cycloadditions to cyclopropenes enables ready access to a wide range of spiro-fused 3-azabicyclo[3.1.0]hexanes (Scheme 1a). Inspired by our recent achievements, we have focused on developing an approach to the synthesis of bis-spiro[3-azabicyclo[3.1.0]hexanes] (compounds containing spiro units at the 2,4-positions of the 3-azabicyclo[3.1.0]hexane moiety) that are a hitherto unknown class of spirocyclic compounds. When considering a synthetic approach to obtaining such compounds, we have turned our attention to a tetrasubstituted azomethine ylide – the *N*-protonated Ruhemann's purple (PRP). This stable ylide was first utilized as a 1,3-dipole in cycloaddition reactions by Grigg and co-workers [27]. This research group demonstrated that the reactions of PRP with dipolarophiles such as *N*-phenylmaleimide, maleic anhydride, and methyl propiolate resulted in the formation of the corresponding bis-spirocyclic cycloadducts (Scheme 1b). It is clear that PRP has been studied in reactions with a small number of dipolarophiles. Accordingly, it is impossible to fully evaluate the synthetic potential of PRP only on the basis of this study. Positioning the current study as a continuation of a series of our works, in which cyclopropenes are utilized as dipolarophiles, we have described a synthetic route to bis-spirocyclic derivatives of 3-azabicyclo[3.1.0]hexane through cyclopropene cycloadditions to stable azomethine ylide PRP (Scheme 1c).

## Results and Discussion

**Scheme 2:**
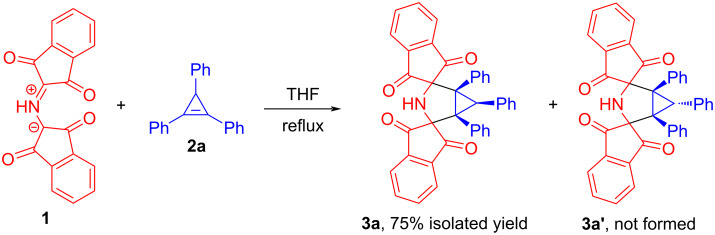
The pilot experiment aimed at studying the cycloaddition reaction between the protonated form of Ruhemann's purple (**1**) and 1,2,3-triphenylcyclopropene (**2a**).

The study commenced with testing the feasibility of the cycloaddition reaction between protonated Ruhemann's purple (PRP, **1**) [28–29] and cyclopropene dipolarophiles **2**. 1,2,3-Triphenylcycloprop-1-ene (TPC, **2a**) [30] was chosen as a model substrate since this alkene had worked well in cycloaddition reactions with various stabilized azomethine ylides [19–24]. Initially, we considered the reaction conditions suggested by Grigg and co-workers in the study [27]. An equimolar mixture of **1** and **2a** was dissolved in tetrahydrofuran (THF), and the resulting mixture was maintained at reflux. After heating for 2 h, completion of the reaction was indicated by both the colour change of the reaction mixture and TLC (thin-layer chromatography) analysis. The proposed cycloadduct was isolated after recrystallization from methanol (MeOH) in 75% yield. Eventually, the constitution of **3a** was unambiguously corroborated by NMR spectra. Given the results of previous studies [22–23] concerning cycloadditions of ninhydrin-derived azomethine ylides to cyclopropenes, it was suggested that the stable azomethine ylide **1** appears to react diastereoselectively with TPC (**2a**), resulting in the formation of bis-spirocyclic meso compound **3a** in which the three phenyl substituents attached to the cyclopropane ring are oriented in the same direction (Scheme 2). This suggests that the diastereomer **3a** resulting from the approach of the 1,3-dipole **1** from the less-hindered face of TPC (**2a**) is more favorable than the opposite diastereoisomer **3a'** (Scheme 2). Subsequently, our hypothesis about the relative configuration of **3a** was confirmed by X-ray analysis that was carried out for the related compound **3e** (vide infra).

Subsequently, our efforts focused on the optimization of the reaction conditions for improving the yield of the cycloadduct **3a**. A broad range of solvents was screened at different temperatures. As presented in Table 1, aprotic solvents such as 1,4-dioxane, acetonitrile, and dimethylformamide (DMF) at 65 °C favored the formation of the desired cycloadduct **3a**. 3-Azabicyclo[3.1.0]hexane derivative **3a** was obtained after recrystallization from MeOH in yields 67, 70, and 61%, respectively (Table 1, entries 2–4). In contrast to aprotic solvents, alcohols such as methanol (MeOH) and ethanol (EtOH) were absolutely unsuitable for carrying out this reaction owing to incompatibility of 1,3-dipole **1** with this medium (Table 1, entries 5 and 6). PRP (**1**) was found to immediately undergo a proton transfer reaction with alcohols, converting it into the conjugate base (Ruhemann's purple). When using dichloromethane as a solvent, the reaction took place at reflux temperature. However, in this case, the yield of **3a** significantly decreased to 42% due to incomplete conversion of **1** and **2a** (Table 1, entry 7). Also, we attempted to carry out this transformation in aprotic solvents at room temperature. Unfortunately, even after 12 h, we failed to completely convert reactants **1** and **2a** into product **3a** in both cases (Table 1, entries 8 and 9). Accordingly, it was concluded that tetrahydrofuran is the most appropriate solvent for carrying out the cycloaddition reaction between **1** and **2a**. To reach full conversion of the reactants, it is necessary to conduct the reaction at reflux for 2 h (Table 1, entry 1).

**Table 1:** Optimization of the reaction conditions^a,b^.

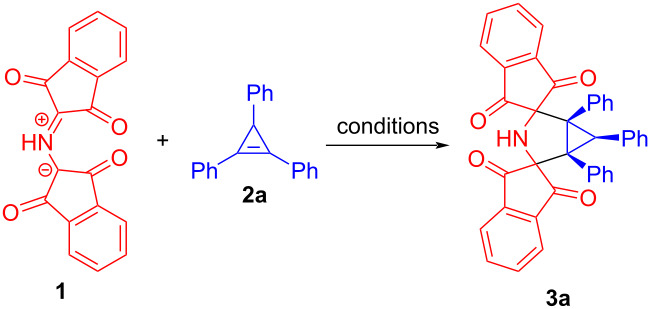				

Entry	Solvent	Temperature (°C)	Time (h)	Yield of **3a** (%)^c^

1	THF	reflux	2	75
2	1,4-dioxane	65	2	67
3	CH_3_CN	65	2	70
4	DMF	65	2	61
5	MeOH	reflux	2	NR^d^
6	EtOH	65	2	NR^d^
7	CH_2_Cl_2_	reflux	12	42
8	THF	rt	12	44
9	CH_3_CN	rt	12	47

^a^Abbreviations: NR – no reaction, rt – room temperature. ^b^Reaction conditions: **1** (1 equiv), **2a** (1 equiv), solvent. ^c^Isolated yield. ^d^Azomethine ylide **1** exclusively underwent deprotonation in protic solvents.

**Scheme 3:**
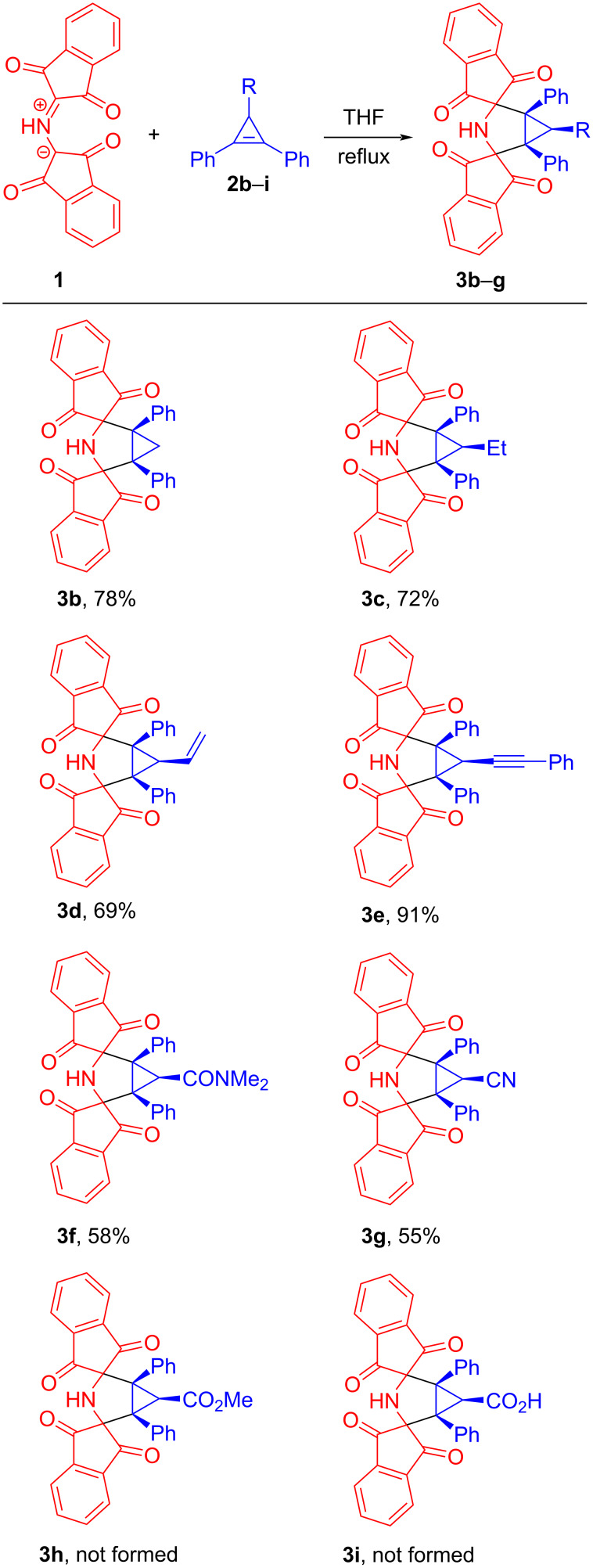
Synthesis of meso-3'-azadispiro[indene-2,2'-bicyclo[3.1.0]hexane-4',2''-indene] derivatives **3b**–**g** via 1,3-DC reactions of *N*-protonated Ruhemann's purple (**1**) with 3-substituted 1,2-diphenylcyclopropenes **2b**–**g**.

**Figure 1:**
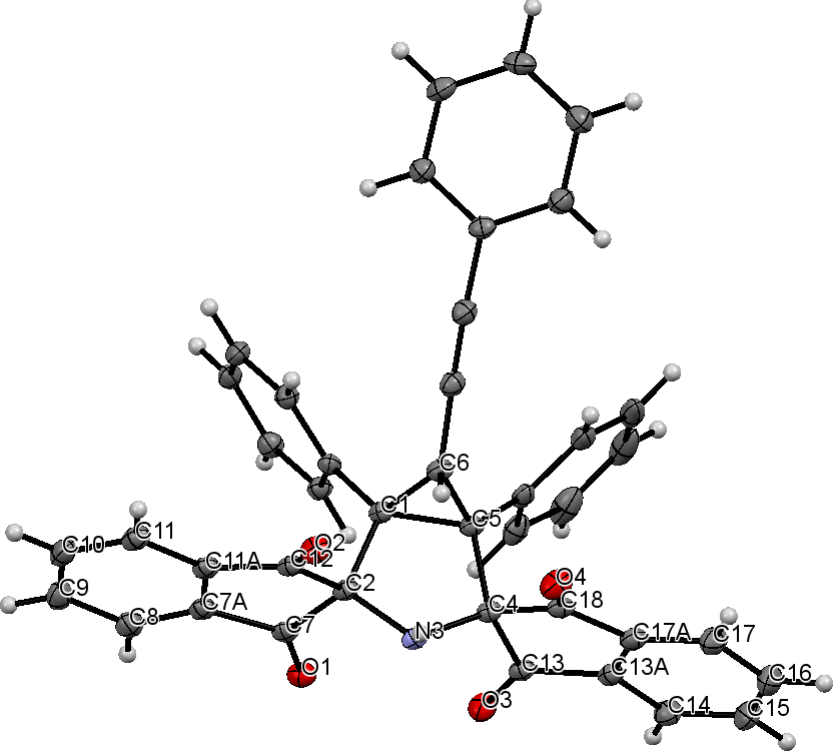
ORTEP representation of the molecular structure of **3e**.

With the optimized conditions in hand, 1,2-diphenylcyclopropenes **2b**–**i** differently substituted at the C3 position were tested as dipolarophiles to evaluate the effect of C3-substituents on the 1,3-DC reaction (Scheme 3). 1,2-Diphenylcyclopropene (**2b**) [31] smoothly underwent the cycloaddition reaction to azomethine ylide **1** to form bis-spiro 3-azabicyclo[3.1.0]hexane **3b** in 78% yield (Scheme 3). Remarkably, the structure of cycloadduct **3b** was additionally verified by X-ray analysis (see Supporting Information File 1, Figure S26 and Table S1). The reaction of 3-ethyl-substituted cyclopropene **2c** [32] with PRP (**1**) also proceeded with full diastereofacial selectivity, giving the corresponding cycloadduct **3c** in acceptable yield (72%). On treatment of cyclopropenes **2d** [32], **2e** [33] both containing multiple bonds with stable azomethine ylide **1**, the 1,3-DC reaction occurred in a highly chemo- and diastereoselective manner and brought about the formation of 3-azabicyclo[3.1.0]hexane cycloadducts **3d** and **3e** in 69% and 91% yields, correspondingly (Scheme 3). As shown by these experiments, the cyclopropene double bond demonstrates higher reactivity compared to exocyclic double or triple bonds due to ring strain. It is noteworthy that we managed to determine the relative configuration of cycloadduct **3e** by carrying out the corresponding X-ray structural analysis (Figure 1 and Table S2 in Supporting Information File 1). As anticipated, the azomethine ylide **1** cycloaddition to cyclopropene **2e** led to the formation of the diastereomer with all three substituents (at the cyclopropane ring) oriented in the same direction.

A notable substituent effect on the reactivity of cyclopropene dipolarophiles **2** was observed for the reactions between PRP (**1**) and derivatives of 2,3-diphenylcycloprop-2-ene-1-carboxylic acid **2f**–**i**. The amide **2f** and nitrile **2g** [34] were found to be less active dipolarophiles towards the azomethine ylide **1** than hydrocarbons **2a**–**e**. As a result, the corresponding cycloadducts **3f** and **3g** were obtained in moderate yields (58% and 55%, respectively) as single diastereomers (Scheme 3). Notably, it took longer reaction times (6 h) for achieving full consumption of **1** while significant amounts of alkenes **2f** and **2g** remained unreacted. In turn, both the ester **2h** and acid **2i** [35] proved to be totally unreactive towards PRP (**1**) under the optimized reaction conditions (THF, reflux). Based on these observations, we concluded that electronic properties of the substituent at the C3 position of a cyclopropene ring have a major impact on the reactivity. A more detailed discussion of this issue is presented in the section devoted to the DFT (density functional theory) computational study. The constitution of compounds **3b**–**g** was established by analyzing ^1^H and ^13^C NMR spectra. In line with the structure of meso compound **3e**, the relative configuration, that is shown in Scheme 3, was assigned to cycloadducts **3a, 3c**, **3d**, **3f**, and **3g**. Thus, the cycloadditions of 3-substituted-1,2-diphenylcyclopropenes **2a**, **2c**–**g** to azomethine ylide **1** were found to exclusively proceed by a pathway in which PRP (**1**) approaches the cyclopropenes **2** from the more sterically available side of **2a, 2c**–**g** (from the side containing a hydrogen substituent).

**Scheme 4:**
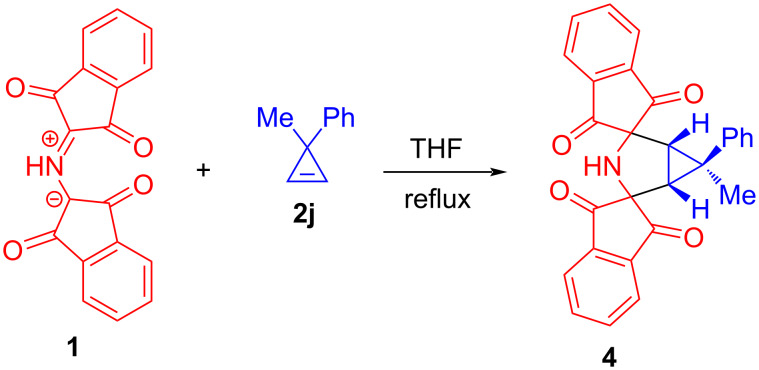
The reaction of protonated Ruhemann's purple (**1**) with 3-methyl-3-phenylcyclopropene (**2j**).

Next, unsymmetrically 3,3-disubstituted cyclopropenes **2j**–**l** were studied in the reaction with **1** to determine whether these 1,3-DC reactions would similarly proceed with high diastereofacial selectivity. The reaction of 3-methyl-3-phenylcyclopropene (**2j**) [36] with ylide **1** resulted in the formation of a 1:1 adduct and the cycloaddition with cyclopropene **2j** occurred at slower rates than with 3-monosubstiuted 1,2-diphenylcyclopropenes. Approximately 6 h at reflux were needed to complete the reaction. The analysis of the ^1^H NMR spectrum of the crude mixture indicated that the formation of cycloadduct **4** occurred with a high level of diastereocontrol (Scheme 4). The other observed signals were at the limit of detection and whether they actually correspond to a minor diastereomer could not be ascertained unambiguously. The major product **4** was purified by recrystallization of the crude mixture from MeOH and obtained in 62% yield. The structure and relative configuration of cycloadduct **4** were unequivocally established on the basis of its two-dimensional (2D) NMR spectrum (^1^H,^1^H nuclear Overhauser effect spectroscopy (NOESY), see Supporting Information File 1, Figures S17–S19). This experiment showed that azomethine ylide **1** also predominantly undergoes cycloaddition to the prochiral cyclopropene **2j** from the less sterically hindered side. This is consistent with previous experiments in which 3-substituted-1,2-diphenylcyclopropenes were utilized as dipolarophiles.

**Scheme 5:**
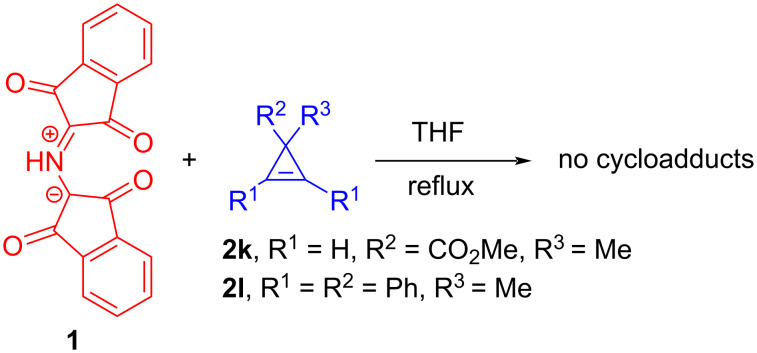
Attempts to carry out the cycloaddition reactions between 3,3-disubstituted cyclopropenes **2k**,**l** and azomethine ylide **1**.

Regretfully, neither methyl 1-methylcycloprop-2-enecarboxylate (**2k**) [37] nor 3-methyl-1,2,3-triphenylcycloprop-1-ene (**2l**) [30] reacted with ylide **1** in THF at reflux (Scheme 5). The inactivity of substrate **2k** appears to be caused by electronic effects while the tetrasubstituted cyclopropene **2l** seems to be too bulky to undergo a cycloaddition reaction with **1**.

**Scheme 6:**
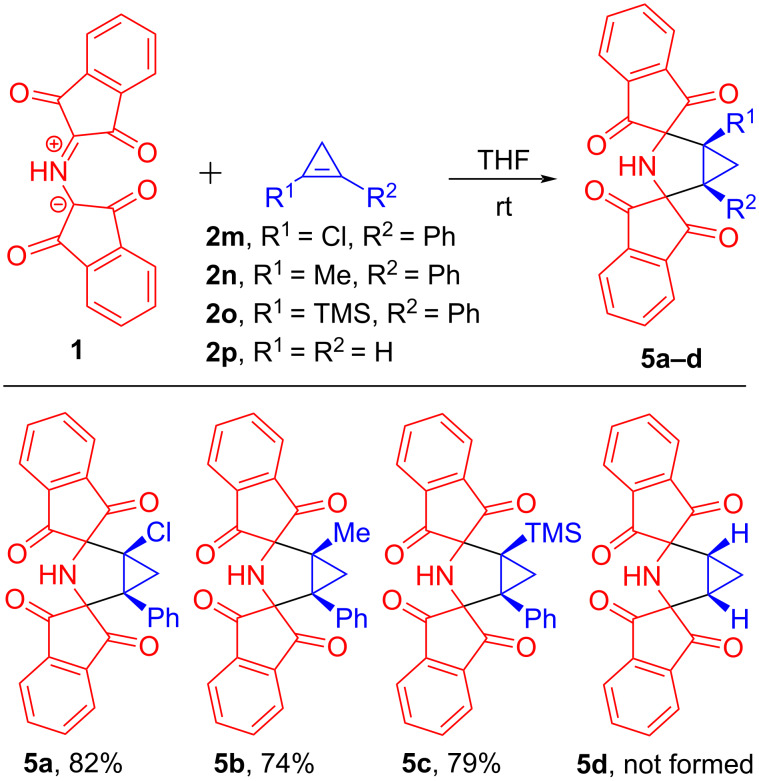
The reactions of protonated Ruhemann's purple (**1**) with unstable cyclopropenes **2m**–**p**.

To the best of our knowledge, PRP (**1**) is one of the few stable azomethine ylides. This fact prompted us to test this azomethine ylide **1** as an effective trap for capturing unstable cyclopropenes (Scheme 6). At first, 1-chloro-2-phenylcyclopropene (**2m**) [38], that is stable only in a solution, was studied as a dipolarophile in reaction with **1**. Thus, a freshly prepared carbon tetrachloride solution of 1-chloro-2-phenylcyclopropene (**2m**) was treated with PRP (**1**) in THF medium. To avoid cyclopropene decomposition, the reaction was carried out at room temperature with stirring. Twelve hours later, we were pleased to note that azomethine ylide **1** was fully consumed as a result of the cycloaddition reaction with cyclopropene **2m**. The corresponding bis-spiro compound **5a** was readily isolated in 82% yield after recrystallization from ethanol. Similarly, we succeeded in carrying out reactions between unsymmetrically 1,2-disubstituted cyclopropenes **2n**, **2o** and PRP (**1**). 1-Methyl-2-phenylcyclopropene (**2n**) and 1-phenyl-2-(trimethylsilyl)cyclopropene (**2o**) synthesized from reliable precursor 1,1,2-tribromo-2-phenylcyclopropane [39–40] were immediately added to a THF solution of nitrogen ylide **1** at room temperature, resulting in the formation of cycloadducts **5b** and **5c** in satisfactory yields (74% and 79%, respectively). Finally, we attempted to trap parent cyclopropene (**2p**) as a cycloadduct with PRP (**1**). In one of our previous studies [22], we have demonstrated that stable ninhydrin-derived azomethine ylide – DHPO (2-(3,4-dihydro-2*H*-pyrrolium-1-yl)-1-oxo-1*H*-inden-3-olate) is an excellent chemical trap for detecting parent cyclopropene. Having carried out the experiment with ylide **1** as a trap in a similar fashion, we found that PRP (**1**) is unreactive towards parent cyclopropene (**2p**) under these conditions. Cyclopropene **2p**, generated *in situ* from allyl chloride [41] and driven into a chilled tube containing PRP (**1**), appeared to undergo free-radical polymerization when increasing the temperature to 25 °C. Despite this failed experiment, in general, PRP (**1**) has established itself as a highly reactive 1,3-dipole towards cyclopropene dipolarophiles **2**.

**Scheme 7:**
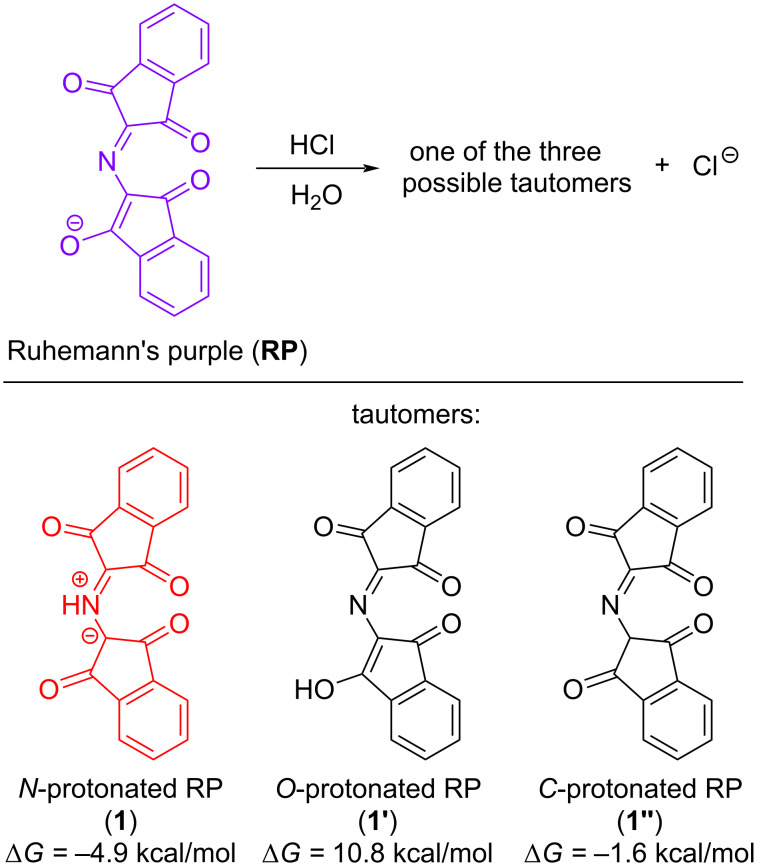
The acid–base reaction of Ruhemann's purple with hydrochloric acid and relative Gibbs free energy change (Δ*G*, kcal/mol) for acid–base reactions resulting in the formation of three protonated forms of Ruhemann's purple.

In this study, we did not confine ourselves exclusively to carrying out laboratory experiments. We turned to DFT calculations (M11 density functional theory) [42–46] to interpret the experimental results. At the beginning of the computational study, we evaluated the relative stability of prototropic tautomers which are formed during protonation of Ruhemann's purple. Although there was conclusive evidence of the structure of PRP (**1**) in the Grigg's study (proven by X-ray analysis) [27], we aimed to establish a stability order of tautomers on the basis of calculated relative values of the Gibbs free energy. Upon treatment of Ruhemann's purple with hydrochloric acid, three tautomers **1**, **1'**, and **1''** may be theoretically formed, i.e., protonation could occur at the nitrogen atom, the oxygen atom or the carbon atom, respectively (Scheme 7). According to literature data [27], the nitrogen atom of Ruhemann's purple is considered to be the most basic site in the molecule. We carried out full geometry optimization of all possible tautomers **1**, **1'**, **1''**, and aza-allylic anion Ruhemann's purple to calculate the Gibbs free energy change for the corresponding acid–base reactions (Scheme 7). As expected, the calculation data showed that the betaine form **1** is the most thermodynamically stable of all tautomers (Δ*G* = −4.9 kcal/mol). It is also not surprising that the *O*-protonated form **1'**, which is both a ketone and an enol, is found to be the most unfavorable (Δ*G* = 10.8 kcal/mol). In contrast to the *O*-protonated tautomer **1'**, both the *N*-protonated **1** and *C*-protonated **1''** tautomers do not contain an enol functional group. It is therefore likely that the lowest stability of the *O*-protonated form **1'** compared to tautomers **1** and **1''** is due to the favorability of the C=O bond over the C=C bond.

Next, using the equations recommended by Parr [47] and Domingo [48], we calculated global electrophilicity indexes (GEI, *ω*) for both PRP (**1**) and cyclopropenes **2** to determine which type of electron demand takes place during cycloaddition reactions. The global value of the electrophilicity index for azomethine ylide **1** (1.29 eV) revealed that this compound displays a moderate electrophilicity (Table 2, entry 1). By comparing the global electrophilicity indexes of reactants **1**, **2a**–**p** (Table 2, entries 1–17), we concluded that the cyclopropene cycloadditions to PRP (**1**) appear to be inverse electron demand (IED) reactions, i.e., they can be considered as the interaction of the lowest unoccupied molecular orbital (LUMO) of the azomethine ylide **1** with the highest occupied molecular orbital (HOMO) of cyclopropenes **2**. It follows that the greater the nucleophilicity of the cyclopropene substrate **2** is, the higher is its reactivity towards the 1,3-dipole **1**. In fact, during the experiments, 1,2-diphenylcyclopropenes **2a**–**e** containing either an alkyl or an alkenyl substituent at the C3 position were found to demonstrate a high reactivity in the 1,3-DC reactions with PRP (**1**). In contrast to substrates **2a**–**e**, cyclopropene dipolarophiles **2f**–**i** bearing electron-withdrawing groups are less nucleophilic. This is evidenced both by fairly large ω values of these cyclopropenes (Table 2, entries 7–10) and by the reaction outcomes. Introducing electron-withdrawing groups at the C3 position of the 1,2-diphenylcyclopropene framework instead of alkyl or alkenyl substituents results in a decrease in the highest occupied molecular orbital (HOMO) and lowest unoccupied molecular orbital (LUMO) energies of such cyclopropenes, thereby increasing the HOMO_cyclopropene_–LUMO_ylide_ energy gap (Table 2, entries 7–10). In practice, this has an impact on the cyclopropene reactivity towards azomethine ylide **1**. For example, the reactions of 2,3-diphenylcyclopropene-1-carboxylic acid derivatives **2f**–**i** with PRP (**1**) either bring about the formation of the corresponding cycloadducts **3** in poor yields or result in the formation of complex mixtures. Thus, having carried out an analysis of global reactivity descriptors, we have found a correlation between the structure of cyclopropene substrates **2** and their reactivity towards PRP (**1**).

**Table 2:** FMO energies (a.u.), electronic chemical potential (μ, eV), chemical hardness (η, eV), and global electrophilicity index (ω, eV) for PRP (**1**) and cyclopropenes **2**.^a,b,c^.

Entry	Compd	HOMO	LUMO	μ	η	ω	NED*^c^*	IED*^c^*	ED

1	**1**	−0.3110	−0.0207	−4.51	7.90	1.29	–	–	–
2	**2a**	−0.2867	0.0614	−3.06	9.47	0.50	10.13	7.24	IED
3	**2b**	−0.2815	0.0637	−2.96	9.39	0.47	10.19	7.10	IED
4	**2c**	−0.2802	0.0650	−2.93	9.39	0.46	10.23	7.06	IED
5	**2d**	−0.2860	0.0612	−3.06	9.45	0.50	10.13	7.22	IED
6	**2e**	−0.2910	0.0591	−3.16	9.53	0.52	10.07	7.35	IED
7	**2f**	−0.2925	0.0557	−3.22	9.47	0.55	9.98	7.39	IED
8	**2g**	−0.2996	0.0526	−3.36	9.58	0.59	9.89	7.59	IED
9	**2h**	−0.2935	0.0581	−3.20	9.56	0.54	10.04	7.42	IED
10	**2i**	−0.2947	0.0571	−3.23	9.57	0.55	10.02	7.45	IED
11	**2j**	−0.3179	0.1212	−2.68	11.95	0.30	11.76	8.09	IED
12	**2k**	−0.3805	0.1350	−3.34	14.03	0.40	12.14	9.79	IED
13	**2l**	−0.2853	0.0618	−3.04	9.44	0.49	10.14	7.20	IED
14	**2m**	−0.3107	0.0820	−3.11	10.68	0.45	10.69	7.89	IED
15	**2n**	−0.2972	0.0944	−2.76	10.66	0.36	11.03	7.52	IED
16	**2o**	−0.3019	0.0824	−2.99	10.46	0.43	10.70	7.65	IED
17	**2p**	−0.3574	0.1556	−2.74	13.96	0.27	12.70	9.16	IED

^a^Abbreviations: HOMO – highest occupied molecular orbital, LUMO – lowest unoccupied molecular orbital, NED – normal electron demand, IED – inverse electron demand, ED – electron demand. ^b^FMO energy values (eV) were computed by using HF/6-311g single point calculation on the M11/cc-pVDZ optimized geometries. ^c^Energy gaps for both possible HOMO–LUMO interactions between PRP (**1**) and cyclopropenes **2** are given in eV.

In the next step, we concentrated on studying the mechanism of the 1,3-DC reactions between PRP (**1**) and cyclopropenes **2**. At first, it was planned to explore the mechanism of 1,3-DC reactions involving cyclopropenes substituted at the C3 position. It is precisely for these cyclopropenes that two diastereomeric cycloadducts can be theoretically obtained. In fact, only one of the two epimers is formed, i.e., the cycloaddition reaction proceeds with complete diastereofacial stereoselectivity. Our goal was to identify the nature of transition states associated with two theoretically possible diastereomers and to find out if the experimentally observed diastereomer is more kinetically favorable than the opposite one.

**Scheme 8:**
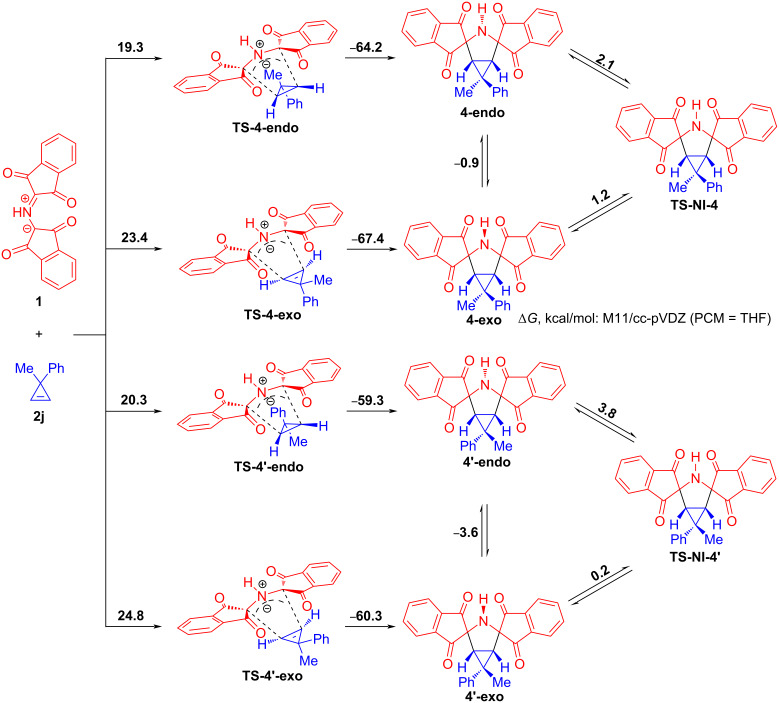
Plausible mechanism of the 1,3-DC reaction of protonated Ruhemann's purple (**1**) with 3-methyl-3-phenylcyclopropene (**2j**) and corresponding DFT calculations (relative Gibbs free energy change between reagents, transition states and possible products are given in kcal/mol).

The reaction between 3-methyl-3-phenylcyclopropene (**2j**) and PRP (**1**) that led to the stereoselective formation of cycloadduct **4** was studied in detail for this purpose (Scheme 8). In analyzing the potential energy surface (PES) of this cycloaddition reaction using the M11 functional together with the cc-pVDZ basis set, we could locate the four transition state structures **ΤS-4-*endo***, **TS-4-*exo***, **TS-4'-*endo***, and **TS-4'-*exo*** that were associated with a concerted mechanism of the 1,3-dipolar cycloaddition. Two of them, namely **TS-4-*endo*** and **TS-4-*exo*** correspond to the two nitrogen invertomers **4-*endo*** and **4-*exo*** of cycloadduct **4** while the other two **TS-4'-*endo*** and **TS-4'-*exo*** associate with nitrogen invertomers **4'-*endo*** and **4'-*exo*** of cycloadduct **4'**, respectively. Next, we determined the Gibbs energy of activation for nitrogen inversion in cycloadducts **4** and **4'** to figure out if the invertomers undergo rapid interconversion. Having carried out full geometry optimization of both transition states **TS-4-NI** and **TS-4'-NI** corresponding to pyramidal inversion in cycloadducts **4** and **4'** and two pairs of invertomers, we have found that each of the diastereomers **4** and **4'** is a mixture of two easily interconverting invertomers (Δ*G*^‡^_4-exo->4-endo_ = 1.2 kcal/mol and Δ*G*^‡^_4-endo->4-exo_ = 2.1 kcal/mol, Δ*G*^‡^_4'-exo->4'-endo_ = 0.2 kcal/mol, and Δ*G*^‡^_4'-endo->4'-exo_ = 3.8 kcal/mol). Since rapid interconversion takes place, it does not matter via which transition state (**ΤS-4-*endo*** or **ΤS-4-*exo***) cycloadduct **4** is formed. The same pattern is consistent with cycloadduct **4'**. It should be mentioned that the endo approaches (Δ*G*^‡^_1+2j->4-endo_ = 19.3 kcal/mol and Δ*G*^‡^_1+2j->4'-endo_ = 20.3 kcal/mol) are much more profitable than the exo ones (Δ*G*^‡^_1+2j->4-exo_ = 23.4 kcal/mol and Δ*G*^‡^_1+2j->4'-exo_ = 24.8 kcal/mol). It follows that cycloadducts **4** and **4'** appear to be formed via **ΤS-4-*endo*** and **TS-4'-endo**, respectively. In turn, the Gibbs energies of activation corresponding to the endo approaches indicate that cycloadduct **4** (Δ*G*^‡^_1+2j->4-endo_ = 19.3 kcal/mol) is more kinetically favorable than its epimer **4'** (Δ*G*^‡^_1+2j->4'-endo_ = 20.3 kcal/mol). The small free energy difference (1.0 kcal/mol) between the two competing pathways is in good agreement with the experimental data and explains the reason why the cycloaddition reaction does not result in the exclusive formation of **4**.

**Scheme 9:**
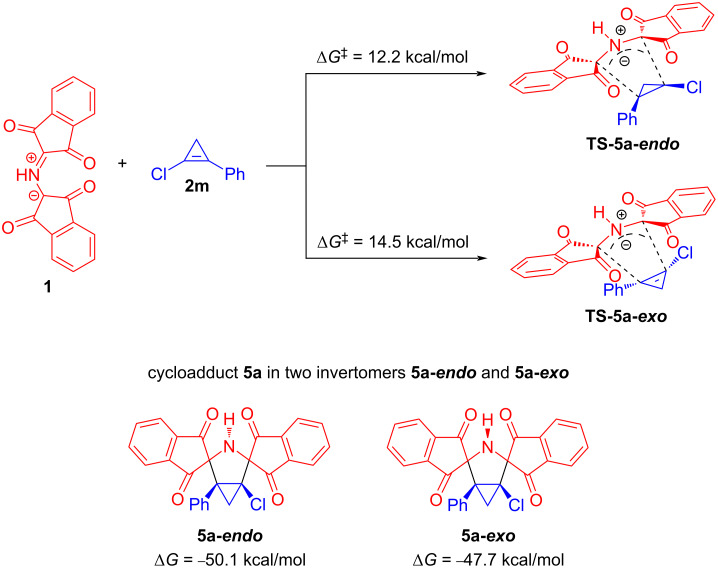
Plausible mechanism of the 1,3-DC reaction of protonated Ruhemann's purple (**1**) with 1-chloro-2-phenylcyclopropene (**2m**) and corresponding DFT calculations (relative Gibbs free energy change between reagents, transition states and possible invertomers are given in kcal/mol).

Additionally, the reaction of PRP (**1**) with 1-chloro-2-phenylcyclopropene (**2m**) was investigated to calculate the Gibbs energy of activation for this cycloaddition reaction and to compare this value with the Gibbs free energy barrier calculated for the reaction between **1** and **2j** (Scheme 9). The azomethine ylide **1** cycloaddition to the chloro-substituted cyclopropene **2m** was also found to proceed by a one-step mechanism via two transition states **TS-5a-endo** and **TS-5a-exo** that bring about invertomers **5a-endo** and **5a-exo** of cycloadduct **5a**, respectively. According to the values of the Gibbs energy of activation, the endo cycloaddition (Δ*G*^‡^ = 12.2 kcal/mol) significantly prevails over the exo one (Δ*G*^‡^ = 14.5 kcal/mol). When comparing the values of the Gibbs energies of activation calculated for the above-mentioned reactions, it was established that the reaction involving **1** and **2m** (Δ*G*^‡^ = 12.2 kcal/mol) should proceed significantly faster than the cycloaddition between **1** and **2j** (Δ*G*^‡^ = 19.3 kcal/mol). Thus, the calculation data are in full accordance with the experimental results, taking into consideration the fact that the reaction between **1** and **2m** smoothly occurs at room temperature while the reaction involving 3-methyl-3-phenylcyclopropene (**2j**) requires harsher conditions.

## Conclusion

In conclusion, we have developed a convenient and diastereoselective approach for the synthesis of bis-spirocyclic derivatives of 3-azabicyclo[3.1.0]hexane through cycloaddition reactions of a stable azomethine ylide – protonated Ruhemann's purple to cyclopropenes. The cycloaddition reaction is compatible with a broad scope of cyclopropenes. DFT calculations revealed that the cycloaddition reactions are under kinetic control and belong to the class of inverse electron demand 1,3-DC reactions. We believe that the outcome of this work will serve as a basis for developing synthetic approaches to other bis-spirocyclic derivatives of 3-azabicyclo[3.1.0]hexane via cycloadditions of tetrasubstituted azomethine ylides with cyclopropenes.

## Supporting Information

File 1 Experimental details for the synthesis and characterization of all compounds, copies of ^1^H NMR and ^13^C NMR spectra, X-ray data and details of calculations.
