# Supplementary material for: Synthesis of bis-spirocyclic derivatives of 3-azabicyclo[3.1.0]hexane via cyclopropene cycloadditions to the stable azomethine ylide derived from Ruhemann's purple
Source: Beilstein J Org Chem. 2022 Jun 29;18:769–80. doi: 10.3762/bjoc.18.77 (PMC9263550; doi:10.3762/bjoc.18.77)
Supplement: File 1 — Experimental details for the synthesis and characterization of all compounds, copies of 1H NMR and 13C NMR spectra, X-ray data and details of calculations. [file Beilstein_J_Org_Chem-18-769-s001.pdf]

BEILSTEIN JOURNAL OF ORGANIC CHEMISTRY

Supporting Information

for

Synthesis of bis-spirocyclic derivatives of 3-azabicyclo[3.1.0]hexane via cyclopropene cycloadditions to the stable azomethine ylide derived from Ruhemann's purple

Alexander S. Filatov, Olesya V. Khoroshilova, Anna G. Larina, Vitali M. Boitsov and Alexander V. Stepakov

Beilstein J. Org. Chem. **2022,** *18,* 769–780. doi:10.3762/bjoc.18.77

Experimental details for the synthesis and characterization of all compounds, copies of ^{1}H NMR and ^{13}C NMR spectra, X-ray data and details of calculations

License and Terms: This is a supporting information file under the terms of the Creative Commons Attribution License (https://creativecommons.org/licenses/by/4.0). Please note that the reuse, redistribution and reproduction in particular requires that the author(s) and source are credited and that individual graphics may be subject to special legal provisions.
The license is subject to the *Beilstein Journal of Organic Chemistry* terms and conditions: (https://www.beilstein-journals.org/bjoc/terms)

Table of contents

1. General information S1
2. Experimental details and characterization data S3
3. Copies of ^{1}H and ^{13}C NMR spectra S9
4. X-ray data for compounds **3b** and **3e** S22
5. Calculation details S25
6. References S46

1. General information

a) Synthesis

Unless otherwise stated, all reagents were purchased from commercial suppliers and used as received. Solvents were dried by standard procedures and freshly distilled prior to use: tetrahydrofuran and 1,4-dioxane were distilled from sodium and benzophenone ketyl, dimethylformamide and dichloromethane from calcium hydride, acetonitrile from phosphorus pentoxide. Hexane and ethyl acetate used for TLC were distilled without pre-treatment with desiccants. Technical grade methanol was dried by heating over iodine-activated magnesium with a magnesium loading of 2.0 g/L. Commercially available ethanol 96% was used without purification. All reactions were carried out under normal atmosphere. The reaction vessels were heated using a silicone oil bath on a magnetic stirrer with a heating plate and a temperature controller. The progress of reactions was monitored by thin-layer chromatography (TLC) on aluminum sheets with 0.2 mm silica gel with fluorescent indicator using UV-light and iodine for visualization. All synthesized compounds were dried under high vacuum (<1 mbar) before determination of chemical yields and spectroscopic characterization.

b) Characterization and analysis

Melting points were measured on a melting point apparatus and are uncorrected. ^{1}H (400 MHz) and ^{13}C (101 MHz) spectra were recorded on an NMR spectrometer in $CDCl_3$ or DMSO-d_6 at ambient temperature. ^{13}C NMR spectra were registered with broad-band proton decoupling. Chemical shifts (δ) in ppm are reported relative to residual undeuterated solvent in $CDCl_3$ (7.26 ppm for ^{1}H and 77.2 ppm for ^{13}C) and DMSO-d_6 (2.50 ppm for ^{1}H and 39.5 ppm for ^{13}C). The signal patterns are indicated as follows: s = singlet, d = doublet, dd = doublet of doublets, t = triplet, dt = doublet of triplets, m = multiplet, br s = broad singlet. Integrals are given in accordance with assignments, coupling constants are reported in Hz. NMR spectra were processed, analyzed, and prepared with MestReNova x64 NMR software. IR spectra were recorded in KBr pellets and reported in wave numbers (cm^{-1}). Electrospray ionization (ESI) mass spectra were measured on a mass spectrometer, HRMS-ESI-QTOF, electrospray ionization, in positive mode.

c) Preparation of starting materials

Stable azomethine ylide protonated Ruhemann's purple (**1**) was readily synthesized by using a two-step procedure. Ruhemann’s purple (as a sodium salt) was obtained by the reaction of ninhydrin and glycine in citrate buffer (0.2 M, pH ≈ 5.0) [1]. In the second step, Ruhemann's purple was treated with concentrated hydrochloric acid in aqueous media, resulting in the target azomethine ylide **1** [2]. The following cyclopropenes were prepared according to the literature data: 1,2,3-

triphenylcyclopropene (**2a**) [3]; 1,2-diphenylcyclopropene (**2b**) [4]; 3-ethyl-1,2-diphenylcyclopropene (**2c**) [5]; 1,2-diphenyl-3-vinylcyclopropene (**2d**) [5]; 1,2-diphenyl-3-(phenylethynyl)cyclopropene (**2e**) [6]; *N*,*N*-dimethyl-2,3-diphenylcycloprop-2-ene-1-carboxamide (**2f**) [7]; 2,3-diphenylcycloprop-2-ene-1-carbonitrile (**2g**) [7]; methyl 2,3-diphenylcycloprop-2-ene-1-carboxylate (**2h**) [8]; 2,3-diphenylcyclop-2-ene-1-carboxylic acid (**2i**) [8]; 3-methyl-3-phenylcyclopropene (**2j**) [9]; methyl 1-methylcycloprop-2-enecarboxylate (**2k**) [10]; 3-methyl-1,2,3-triphenylcyclopropene (**2l**) [3]; 1-chloro-2-phenylcyclopropene (**2m**) [11]; 1-methyl-2-phenylcyclopropene (**2n**) [12]; 1-phenyl-2-(trimethylsilyl)cyclopropene (**2o**) [13]; and parent cyclopropene (**2p**) [14].

2. Experimental details and characterization data

General procedure A for the preparation of cycloadducts 3a–g, and **4:** Protonated Ruhemann's purple (**1**, 121 mg, 0.400 mmol) and cyclopropene **2a–g, 2j** (0.400 mmol) were dissolved in THF (15 mL). The reaction mixture was heated at reflux for 2–6 h and then cooled to room temperature. The mixture was filtered through a plug of celite to remove trace amounts of an insoluble dark brown solid. The plug of celite was carefully rinsed with THF (20 mL). The filtrate was evaporated to dryness under vacuum. The crude residue was purified by recrystallization from a suitable solvent to obtain cycloadducts **3a–g**, **4**.

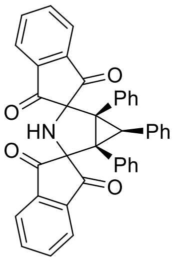

3a

***meso*-(1'*R*,5'*S*,6'*r*)-1',5',6'-Triphenyl-3'-azadispiro[indene-2,2'-bicyclo[3.1.0]hexane-4',2''-indene]-1,1'',3,3''-tetraone (3a)**

Cycloadduct **3a** was obtained as a single diastereomer according to General procedure A from protonated Ruhemann's Purple (**1**, 121 mg, 0.400 mmol) and cyclopropene **2a** (107 mg, 0.400 mmol). The reaction mixture was refluxed for 2 h to achieve a satisfactory degree of cyclopropene conversion. The crude product was purified by recrystallization from ethanol, giving rise to pure **3a** in 75% yield (171 mg); yellow solid; mp > 300 °C (EtOH); R_f 0.42 (SiO_2, hexane–EtOAc, 1:1).

IR (KBr, cm^{-1}): 3358, 3093, 3053, 3034, 1745, 1715, 1592, 1495, 1444, 1353, 1324, 1257, 1205, 1158, 1096, 1081, 1040, 1018, 998, 972, 791, 755, 697.

^{1}H NMR (400 MHz, DMSO-d_6): δ = 7.89–7.84 (m, 2 H), 7.81–7.75 (m, 2 H), 7.74–7.68 (m, 2 H), 7.48–7.42 (m, 2 H), 7.02–6.68 (m, 13 H), 6.17–6.10 (m, 2 H), 4.39 (s, 1 H), 4.05 (s, 1 H).

^{13}C NMR (101 MHz, DMSO-d_6): δ = 199.14 (2 C), 199.09 (2 C), 141.0 (2 C), 139.8 (2 C), 136.4 (2 C), 135.7 (2 C), 135.1, 133.1 (4 C), 130.6 (2 C), 129.6 (2 C), 127.33 (2 C), 127.26 (4 C), 126.5 (2 C), 125.5, 122.4 (4 C), 79.1 (2 C), 53.0 (2 C), 28.0.

HRMS (ESI): calcd. for $C_{39}H_{25}NNaO_4^+$ [M + Na]$^+$: 594.1676; found: 594.1670.

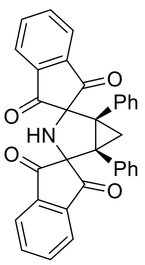

3b

***meso*-(1'*R*,5'*S*)-1',5'-Diphenyl-3'-azadispiro[indene-2,2'-bicyclo[3.1.0]hexane-4',2''-indene]-1,1'',3,3''-tetraone (3b)**

Cycloadduct **3b** was obtained according to General procedure A from protonated Ruhemann's Purple (**1**, 121 mg, 0.400 mmol) and cyclopropene **2b** (77 mg, 0.400 mmol). The reaction mixture was refluxed for 2 h to achieve a satisfactory degree of cyclopropene conversion. The crude product was purified by recrystallization from methanol, giving rise to pure **3b** in 78% yield (155 mg); beige solid; mp 252–253 °C (MeOH); R_f 0.41 (SiO_2, hexane–EtOAc, 1:1).

IR (KBr, cm^{-1}): 3318, 3067, 3022, 2960, 2879, 1747, 1717, 1596, 1494, 1447, 1344, 1262, 1193, 1113, 1080, 1056, 1025, 1004, 943, 800, 773, 749, 732, 705.

^{1}H NMR (400 MHz, DMSO-d_6): δ = 7.90–7.86 (m, 2 H), 7.82–7.72 (m, 4 H), 7.59–7.56 (m, 2 H), 7.10–7.05 (m, 4 H), 6.97–6.87 (m, 6 H), 3.71 (s, 1 H), 2.86 (d, J = 5.5 Hz, 1 H), 1.29 (d, J = 5.5 Hz, 1 H).

^{13}C NMR (101 MHz, DMSO-d_6): δ = 199.6 (2 C), 198.7 (2 C), 141.0 (2 C), 139.4 (2 C), 136.5 (2 C), 135.8 (2 C), 133.7 (2 C), 131.8 (4 C), 127.6 (4 C), 127.6 (2 C), 122.49 (2 C), 122.48 (2 C), 78.7 (2 C), 48.7 (2 C), 15.2.

HRMS (ESI): calcd. for $C_{33}H_{22}NO_4^+$ [M + H]$^+$: 496.1543; found: 496.1553.

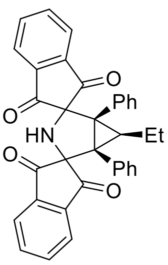

3c

***meso*-(1'*R*,5'*S*,6'*r*)-6'-Ethyl-1',5'-diphenyl-3'-azadispiro[indene-2,2'-bicyclo[3.1.0]hexane-4',2''-indene]-1,1'',3,3''-tetraone (3c)**

Cycloadduct **3c** was obtained as a single diastereomer according to General procedure A from protonated Ruhemann's Purple (**1**, 121 mg, 0.400 mmol) and cyclopropene **2c** (88 mg, 0.400 mmol). The reaction mixture was refluxed for 2 h to achieve a satisfactory degree of cyclopropene conversion. The crude product was purified by recrystallization from ethanol, giving rise to pure **3c** in 72% yield (151 mg); yellow solid; mp 259–261 °C (EtOH); R_f 0.45 (SiO_2, hexane–EtOAc, 1:1).

IR (KBr, cm^{-1}): 3296, 3054, 2965, 2921, 2869, 1748, 1715, 1599, 1493, 1443, 1346, 1324, 1278, 1257, 1192, 1152, 1082, 1021, 1003, 952, 776, 762, 701.

^{1}H NMR (400 MHz, DMSO-d_6): δ = 8.00–7.94 (m, 2 H), 7.90–7.83 (m, 2 H), 7.82–7.75 (m, 2 H), 7.54–7.47 (m, 2 H), 7.10–6.90 (m, 10 H), 3.90 (s, 1 H), 2.93 (t, J = 6.5 Hz, 1 H), 1.08–0.98 (m, 2 H), 0.90 (t, J = 7.0 Hz, 3 H).

^{13}C NMR (101 MHz, DMSO-d_6): δ = 199.1 (2 C), 198.9 (2 C), 141.2 (2 C), 140.0 (2 C), 136.6 (2 C), 135.7 (2 C), 132.3 (2 C), 131.5 (4 C), 127.5 (4 C), 127.0 (2 C), 122.7 (2 C), 122.6 (2 C), 78.2 (2 C), 49.7 (2 C), 25.2, 19.1, 13.6.

HRMS (ESI): calcd. for $C_{35}H_{26}NO_4^+$ [M + H]$^+$: 524.1856; found: 524.1857.

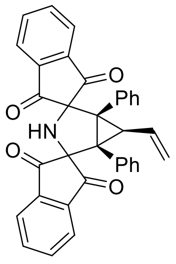

3d

***meso*-(1'*R*,5'*S*,6'*r*)-1',5'-Diphenyl-6'-vinyl-3'-azadispiro[indene-2,2'-bicyclo[3.1.0]hexane-4',2''-indene]-1,1'',3,3''-tetraone (3d)**

Cycloadduct **3d** was obtained as a single diastereomer according to General procedure A from protonated Ruhemann's Purple (**1**, 121 mg, 0.400 mmol) and cyclopropene **2d** (87 mg, 0.400 mmol). The reaction mixture was refluxed for 2 h to achieve a satisfactory degree of cyclopropene conversion. The crude product was purified by recrystallization from ethanol, giving rise to pure **3d** in 69% yield (144 mg); yellow solid; mp > 300 °C (EtOH); R_f 0.44 (SiO_2, hexane–EtOAc, 1:1).

IR (KBr, cm^{-1}): 3355, 3105, 3052, 2979, 1746, 1717, 1594, 1493, 1445, 1351, 1325, 1259, 1206, 1156, 1095, 1043, 993, 908, 754, 704.

^{1}H NMR (400 MHz, DMSO-d_6): δ = 7.92–7.88 (m, 2 H), 7.83–7.77 (m, 2 H), 7.75–7.70 (m, 2 H), 7.48–7.43 (m, 2 H), 7.18–6.88 (m, 10 H), 5.38 (dd, J = 17.1, 1.1 Hz, 1 H), 5.00 (dd, J = 10.3, 1.1 Hz, 1 H), 4.47 (dt, J = 17.1, 10.3 Hz, 1H), 3.88 (s, 1 H), 3.83 (d, J = 10.3 Hz, 1 H).

^{13}C NMR (101 MHz, DMSO-d_6): δ = 199.1 (2 C), 198.7 (2 C), 141.0 (2 C), 139.7 (2 C), 136.4 (2 C), 135.7 (2 C), 135.6, 132.7 (4 C), 131.0 (2 C), 127.6 (4 C), 127.4 (2 C), 122.45 (2 C), 122.41 (2 C), 115.8, 78.9 (2 C), 51.4 (2 C), 27.1.

HRMS (ESI): calcd. for $C_{35}H_{23}NNaO_4^+$ [M + Na]$^+$: 544.1519; found: 544.1537.

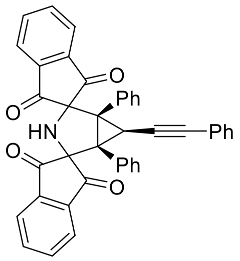

3e

meso-(1'_R_,5'_S_,6'_r_)-1',5'-Diphenyl-6'-(phenylethynyl)-3'-azadispiro[indene-2,2'-bicyclo[3.1.0]hexane-4',2''-indene]-1,1'',3,3''-tetraone (3e)

Cycloadduct **3e** was obtained as a single diastereomer according to General procedure A from protonated Ruhemann's Purple (**1**, 121 mg, 0.400 mmol) and cyclopropene **2e** (117 mg, 0.400 mmol). The reaction mixture was refluxed for 2 h to achieve a satisfactory degree of cyclopropene conversion. The crude product was purified by recrystallization from ethanol, giving rise to pure **3e** in 91% yield (217 mg); yellow solid; mp > 300 °C (EtOH); R_f 0.42 (SiO_2, hexane–EtOAc, 1:1).

IR (KBr, cm^{-1}): 3286, 3059, 3026, 2873, 1750, 1722, 1594, 1493, 1444, 1348, 1259, 1192, 1155, 1080, 1021, 798, 760, 703.

^{1}H NMR (400 MHz, DMSO-d_6): δ = 7.98–7.90 (m, 2 H), 7.87–7.80 (m, 2 H), 7.79–7.72 (m, 2 H), 7.54–7.48 (m, 2 H), 7.27–7.13 (m, 7 H), 7.06–6.91 (m, 8 H), 4.13 (s, 1 H), 4.09 (s, 1 H).

^{13}C NMR (101 MHz, DMSO-d_6): δ = 198.9 (2 C), 198.8 (2 C), 140.8 (2 C), 139.6 (2 C), 136.5 (2 C), 135.8 (2 C), 132.6 (4 C), 130.7 (2 C), 130.2 (2 C), 128.3 (2 C), 128.2, 127.6 (2 C), 127.4 (4 C), 122.5 (4 C), 122.4, 87.3, 86.4, 77.9 (2 C), 52.4 (2 C), 15.1.

HRMS (ESI): calcd. for $C_{41}H_{25}NNaO_4^+$ [M + Na]$^+$: 618.1676; found: 618.1647.

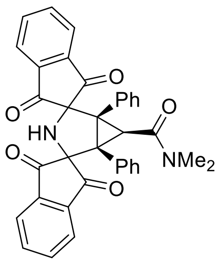

3f

meso-(1'_R_,5'_S_,6'_r_)-_N_,_N_-Dimethyl-1,1'',3,3''-tetraoxo-1',5'-diphenyl-1,1'',3,3''-tetrahydro-3'-azadispiro[indene-2,2'-bicyclo[3.1.0]hexane-4',2''-indene]-6'-carboxamide (3f)

Cycloadduct **3f** was obtained as a single diastereomer according to General procedure A from protonated Ruhemann's Purple (**1**, 121 mg, 0.400 mmol) and cyclopropene **2f** (105 mg, 0.400 mmol). The reaction mixture was refluxed for 6 h to achieve a satisfactory degree of cyclopropene conversion. The crude product was purified by recrystallization from methanol, giving rise to pure **3f** in 58% yield (131 mg); yellow solid; mp 275–278 °C (MeOH); R_f 0.13 (SiO_2, hexane–EtOAc, 1:1).

IR (KBr, cm^{-1}): 3306, 3077, 3051, 3025, 2937, 1746, 1710, 1639, 1598, 1493, 1447, 1402, 1349, 1327, 1260, 1204, 1157, 1094, 1021, 781, 761, 705.

^{1}H NMR (400 MHz, DMSO-d_6): δ = 7.94–7.88 (m, 2 H), 7.84–7.78 (m, 2 H), 7.76–7.71 (m, 2 H), 7.46–7.41 (m, 2 H), 6.97–6.77 (m, 10 H), 4.46 (s, 1 H), 4.06 (s, 1 H), 3.57 (s, 3 H), 2.65 (s, 3 H).

^{13}C NMR (101 MHz, DMSO-d_6): δ = 199.7 (2 C), 199.0 (2 C), 166.1, 140.9 (2 C), 139.7 (2 C), 136.5 (2 C), 135.7 (2 C), 131.7 (4 C), 131.0 (2 C), 126.8 (4 C), 126.7 (2 C), 122.4 (4 C), 78.9 (2 C), 53.9 (2 C), 37.4, 35.3, 22.2.

HRMS (ESI): calcd. for $C_{36}H_{26}N_2NaO_5^+$ $[M + Na]^+$: 589.1734; found: 589.1731.

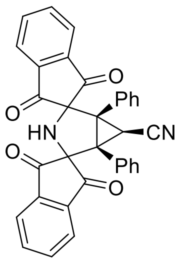

3g

***meso*-(1'*R*,5'*S*,6'*r*)-1,1'',3,3''-Tetraoxo-1',5'-diphenyl-1,1'',3,3''-tetrahydro-3'-azadispiro[indene-2,2'-bicyclo[3.1.0]hexane-4',2''-indene]-6'-carbonitrile (3g)**

Cycloadduct **3g** was obtained as a single diastereomer according to General procedure A from protonated Ruhemann's Purple (**1**, 121 mg, 0.400 mmol) and cyclopropene **2g** (87 mg, 0.400 mmol). The reaction mixture was refluxed for 6 h to achieve a satisfactory degree of cyclopropene conversion. The crude product was purified by recrystallization from ethanol, giving rise to pure **3g** in 55% yield (115 mg); yellow solid; mp > 300 °C (EtOH); R_f 0.35 (SiO_2, hexane–EtOAc, 1:1).

IR (KBr, cm^{-1}): 3287, 3078, 3055, 3014, 2924, 2878, 2238, 1751, 1716, 1592, 1446, 1351, 1261, 1206, 1160, 1081, 1023, 1002, 944, 779, 758, 700.

^{1}H NMR (400 MHz, DMSO-d_6): δ = 7.98–7.90 (m, 2 H), 7.88–7.81 (m, 2 H), 7.80–7.74 (m, 2 H), 7.58–7.50 (m, 2 H), 7.22–7.15 (m, 4 H), 7.13–7.01 (m, 6 H), 4.30 (s, 1 H), 4.25 (s, 1 H).

^{13}C NMR (101 MHz, DMSO-d_6): δ = 198.55 (2 C), 198.5 (2 C), 140.6 (2 C), 139.4 (2 C), 136.7 (2 C), 136.0 (2 C), 131.9 (4 C), 128.6 (2 C), 128.5 (2 C), 128.1 (4 C), 122.7 (2 C), 122.5 (2 C), 117.2, 76.7 (2 C), 51.4 (2 C), 11.8.

HRMS (ESI): calcd. for $C_{34}H_{20}N_2NaO_4^+$ $[M + Na]^+$: 543.1315; found: 543.1306.

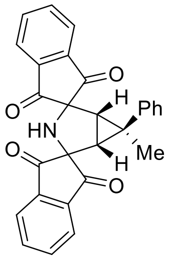

4

***meso*-(1'*R*,5'*S*,6'*r*)-6'-Methyl-6'-phenyl-3'-azadispiro[indene-2,2'-bicyclo[3.1.0]hexane-4',2''-indene]-1,1'',3,3''-tetraone (4)**

Cycloadduct was obtained as a single diastereomer **4** from protonated Ruhemann's Purple (**1**, 121 mg, 0.400 mmol) and cyclopropene **2j** (78 mg, 0.6 mmol). The reaction mixture was refluxed for 6 h to achieve a satisfactory degree of cyclopropene conversion. The crude product was purified by recrystallization from methanol, giving rise to pure diastereomer **4** in 62% yield (107 mg); beige solid; mp > 300 °C (MeOH); R_f 0.36 (SiO_2, hexane–EtOAc, 1:1).

IR (KBr, cm^{-1}): 3352, 3073, 3050, 3030, 2964, 2885, 1745, 1719, 1596, 1497, 1432, 1343, 1264, 1198, 1154, 1058, 960, 780, 747, 692.

^{1}H NMR (400 MHz, $CDCl_3$): δ = 8.13–8.01 (m, 4 H, C^4H ($C^{7''}$ H) + C^7H ($C^{4''}$ H)), 7.95–7.86 (m, 4 H, C^5H ($C^{6''}$ H) + C^6H ($C^{5''}$ H)), 7.21–7.13 (m, 2 H, C^9H ($C^{11}H$)), 7.12–7.05 (m, 1 H, $C^{10}H$), 6.98–6.87 (m, 2 H, C^8H ($C^{12}H$)), 3.26 (br s, 1 H, NH), 2.16 (s, 2 H, $C^{1'}H$ ($C^{5'}H$)), 1.93 (s, 3 H, CH_3–$C^{6'}$).

^{13}C NMR (101 MHz, $CDCl_3$): δ = 197.5 (2 C), 197.2 (2 C), 146.3, 141.2 (2 C), 140.5 (2 C), 136.5 (2 C), 136.0 (2 C), 128.6 (2 C), 126.58, 126.56 (2 C), 124.4 (2 C), 123.9 (2 C), 76.9 (2 C), 39.9 (2 C), 33.3, 18.4.

HRMS (ESI): calcd. for $C_{28}H_{19}NNaO_4^+$ [M + Na]$^+$: 456.1206; found: 456.1211.

General procedure B for the preparation of cycloadducts 5a–c: Cyclopropene **2m** as a solution in carbon tetrachloride or cyclopropene **2n**, **2o** (0.600 mmol) that had been just prepared from the corresponding precursors was added to a solution of protonated Ruhemann's purple (**1**, 121 mg, 0.40000 mmol) in anhydrous THF (15 mL) with stirring at room temperature. After detecting decolorization of solution (24 h later), the reaction mixture was filtered through a plug of celite, and the latter was rinsed with 20 mL of THF. The solvent was distilled off to obtain crude product **5** which was eventually recrystallized from a suitable solvent.

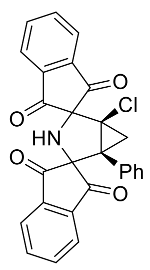

5a

(±)-(1'*R*,5'*R*)-1'-Chloro-5'-phenyl-3'-azadispiro[indene-2,2'-bicyclo[3.1.0]hexane-4',2''-indene]-1,1'',3,3''-tetraone (5a)

Cycloadduct **5a** was obtained according to General procedure B from protonated Ruhemann's purple (**1**, 121 mg, 0.400 mmol) and cyclopropene **2m** (90 mg, 0.6 mmol). The crude product was purified by recrystallization from ethanol, giving rise to pure **5a** in 82% yield (149 mg); yellow solid; mp > 300 °C (EtOH); R_f 0.42 (SiO_2, hexane–EtOAc, 1:1).

IR (KBr, cm^{-1}): 3365, 3088, 3064, 3031, 1746, 1720, 1598, 1495, 1447, 1425, 1340, 1268, 1229, 1201, 1157, 1043, 1027, 1004, 963, 913, 790, 758, 721, 701.

^{1}H NMR (400 MHz, $CDCl_3$): δ = 8.17 (d, *J* = 7.5 Hz, 1 H), 8.09 (d, *J* = 7.5 Hz, 1 H), 8.00–7.88 (m, 3 H), 7.72–7.66 (m, 1 H), 7.62–7.56 (m, 1 H), 7.43 (d, *J* = 7.6 Hz, 1 H), 7.12–7.03 (m, 5 H), 2.94 (br s, 1 H), 2.87 (d, *J* = 7.1 Hz, 1 H), 1.31 (d, *J* = 7.1 Hz, 1 H).

^{13}C NMR (101 MHz, $CDCl_3$): δ = 198.3, 196.9, 196.7, 196.5, 142.5, 142.2, 140.9, 139.6, 137.3, 136.5, 136.1, 135.3, 132.3 (2 C), 132.0, 128.4, 128.3 (2 C), 124.2, 123.9, 123.3, 123.1, 79.6, 74.3, 55.5, 48.5, 19.6.

HRMS (ESI): calcd. for $C_{27}H_{16}ClNNaO_4^+$ [M + Na]$^+$: 476.0660; found: 476.0659.

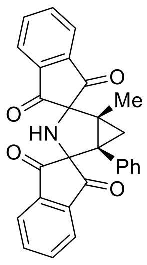

5b

(±)-(1'*R*,5'*R*)-1'-Methyl-5'-phenyl-3'-azadispiro[indene-2,2'-bicyclo[3.1.0]hexane-4',2''-indene]-1,1'',3,3''-tetraone (5b)

Cycloadduct **5b** was obtained according to General procedure B from protonated Ruhemann's purple (**1**, 121 mg, 0.400 mmol) and cyclopropene **2n** (78 mg, 0.6 mmol). The crude product was purified by recrystallization from methanol, giving rise to pure **5b** in 74% yield (128 mg); yellow solid; mp 280–282 °C (MeOH); R_f 0.37 (SiO_2, hexane–EtOAc, 1:1).

IR (KBr, cm^{-1}): 3379, 3082, 3057, 3026, 2924, 2852, 1745, 1716, 1590, 1442, 1350, 1253, 1200, 1159, 1033, 793, 756, 727, 703.

^{1}H NMR (400 MHz, $CDCl_3$): δ = 8.14 (d, J = 7.3 Hz, 1 H), 8.06 (d, J = 7.3 Hz, 1 H), 7.98–7.85 (m, 3 H), 7.68–7.62 (m, 1 H), 7.58–7.52 (m, 1 H), 7.44–7.38 (m, 1 H), 7.04–6.96 (m, 5 H), 3.23 (br s, 1 H), 2.39 (d, J = 5.9 Hz, 1 H), 0.78 (s, 3 H), 0.65 (d, J = 5.9 Hz, 1 H).

^{13}C NMR (101 MHz, $CDCl_3$): δ = 199.9, 199.5, 198.4, 197.5, 142.0, 141.8, 140.7, 139.8, 136.7, 135.9 (2 C), 134.9, 134.0, 132.0 (2 C), 128.0 (2 C), 127.6, 123.7, 123.4, 122.9, 122.7, 81.2, 75.9, 49.0, 39.3, 16.7, 16.0.

HRMS (ESI): calcd. for $C_{28}H_{20}NO_4^+$ $[M + H]^+$: 434.1387; found: 434.1396.

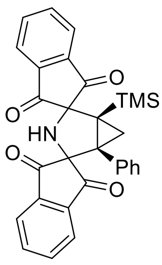

5c

(±)-(1'*R*,5'*R*)-1'-Phenyl-5'-(trimethylsilyl)-3'-azadispiro[indene-2,2'-bicyclo[3.1.0]hexane-4',2''-indene]-1,1'',3,3''-tetraone (5c)

Cycloadduct **5c** was obtained according to General procedure B from protonated Ruhemann's purple (**1**, 121 mg, 0.400 mmol) and cyclopropene **2o** (113 mg, 0.6 mmol). The crude product was purified by recrystallization from methanol, giving rise to pure **5c** in 79% yield (155 mg); yellow solid; mp 245–247 °C (MeOH); R_f 0.51 (SiO_2, hexane–EtOAc, 1:1).

IR (KBr, cm^{-1}): 3374, 3085, 2955, 2897, 1746, 1718, 1598, 1448, 1348, 1274, 1215, 1159, 1039, 959, 838, 794, 760, 703.

^{1}H NMR (400 MHz, $CDCl_3$): δ = 8.15 (d, J = 7.2 Hz, 1 H), 8.03 (d, J = 7.2 Hz, 1 H), 7.98–7.87 (m, 2 H), 7.84 (d, J = 7.4 Hz, 1 H), 7.64–7.58 (m, 1 H), 7.55–7.49 (m, 1 H), 7.39 (d, J = 7.4 Hz, 1 H), 7.22–7.02 (m, 2 H), 7.00–6.91 (m, 3 H), 2.41 (d, J = 5.6 Hz, 1 H), 2.27 (br s, 1 H), 0.92 (d, J = 5.6 Hz, 1 H), –0.60 (s, 9 H).

^{13}C NMR (101 MHz, $CDCl_3$): δ = 201.2, 199.3, 198.8, 197.3, 142.5, 141.6, 141.2, 140.0, 136.9, 136.2, 136.0, 135.6, 134.9, 131.7 (2 C), 128.0 (2 C), 127.8, 124.3, 123.6, 123.1, 122.7, 82.5, 74.8, 51.7, 33.2, 12.2, –1.1 (3 C).

HRMS (ESI): calcd. for $C_{30}H_{26}NO_4Si^+$ $[M + H]^+$: 492.1626; found: 492.1637.

3. Copies of ^{1}H and ^{13}C NMR spectra

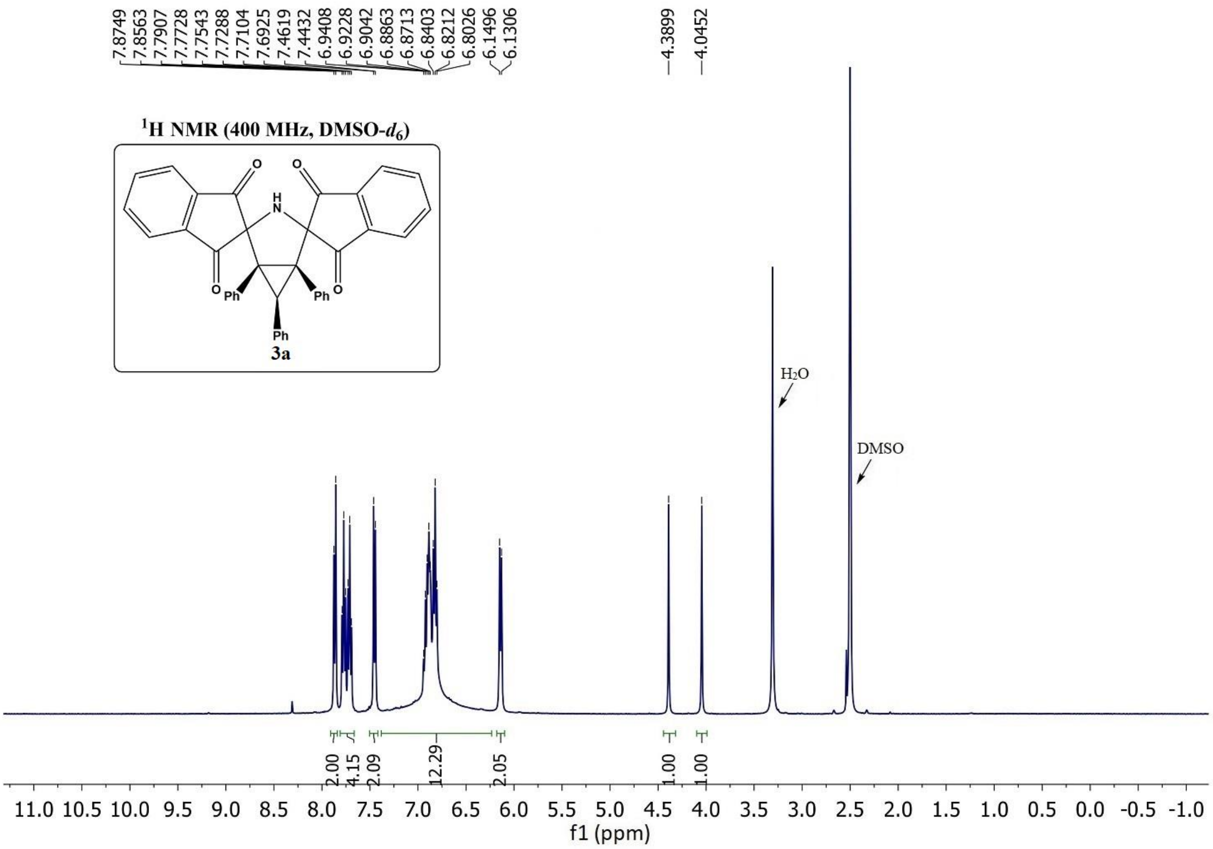

Figure S1: ^{1}H NMR spectrum of compound **3a** (400 MHz, DMSO-d_6)

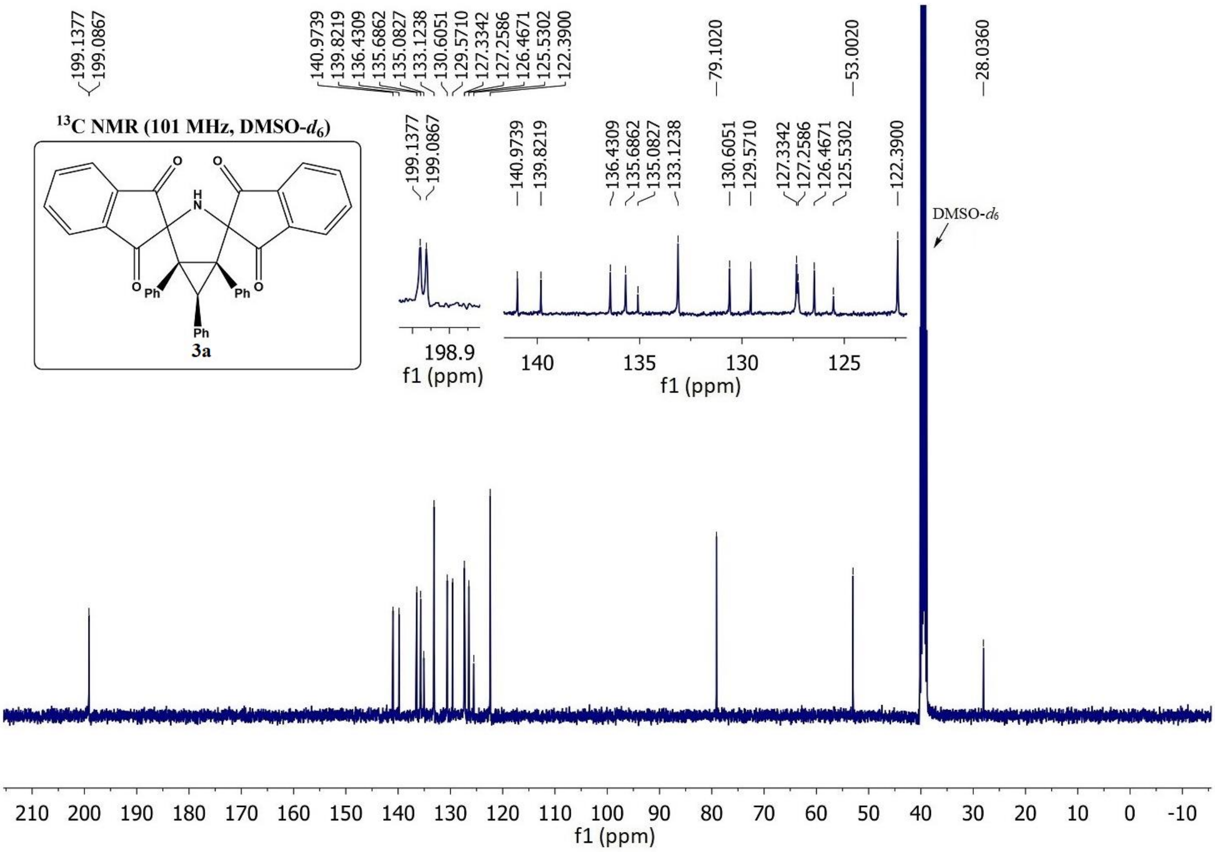

Figure S2: ^{13}C NMR spectrum of compound **3a** (101 MHz, DMSO-d_6)

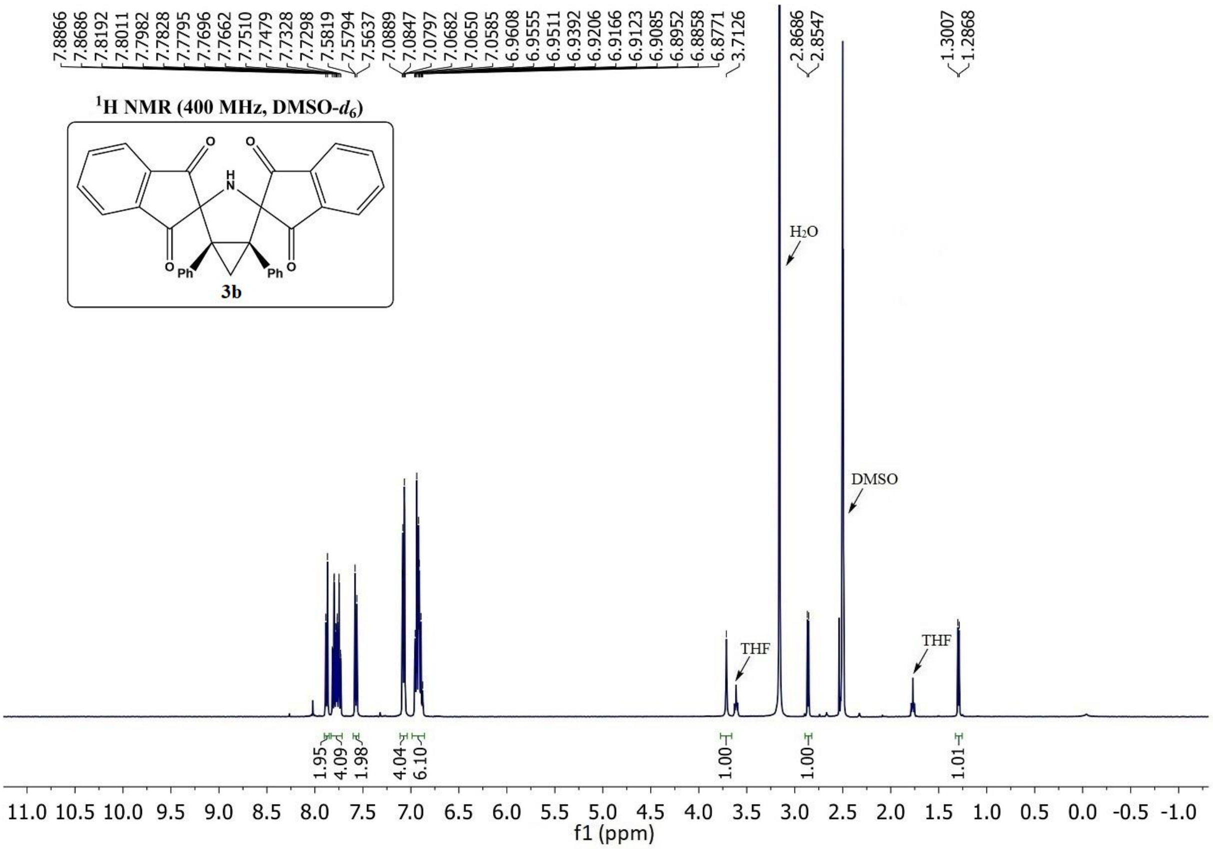

Figure S3: ^{1}H NMR spectrum of compound **3b** (400 MHz, DMSO-d_6)

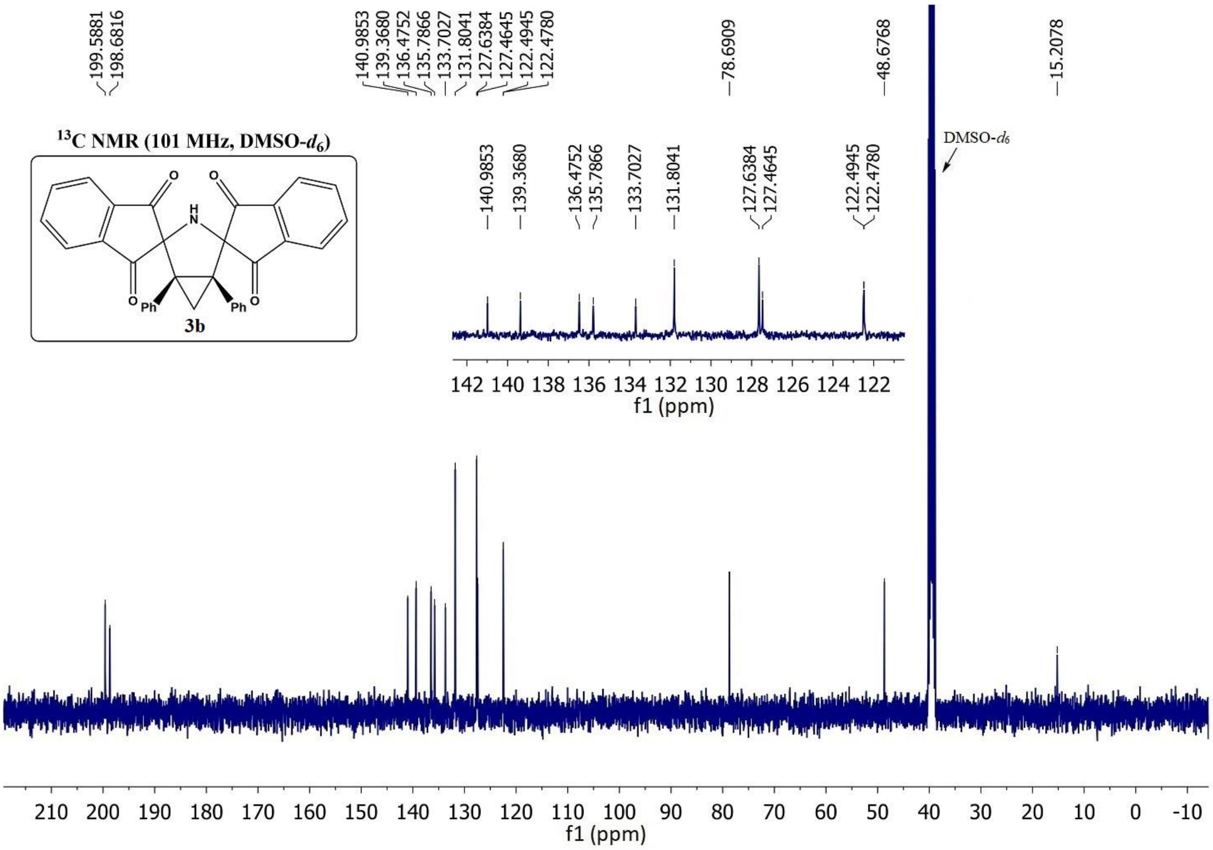

Figure S4: ^{13}C NMR spectrum of compound **3b** (101 MHz, DMSO-d_6)

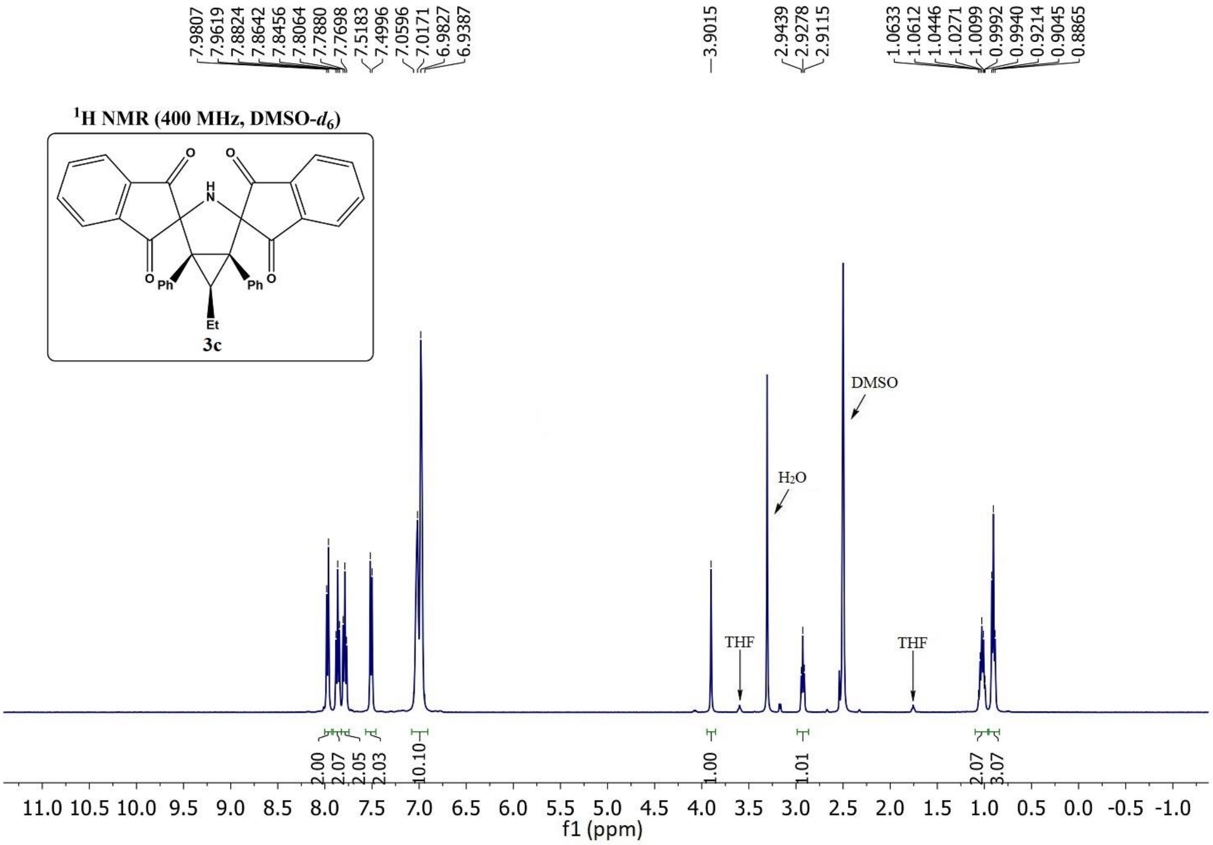

Figure S5: ^{1}H NMR spectrum of compound **3c** (400 MHz, DMSO-d_6)

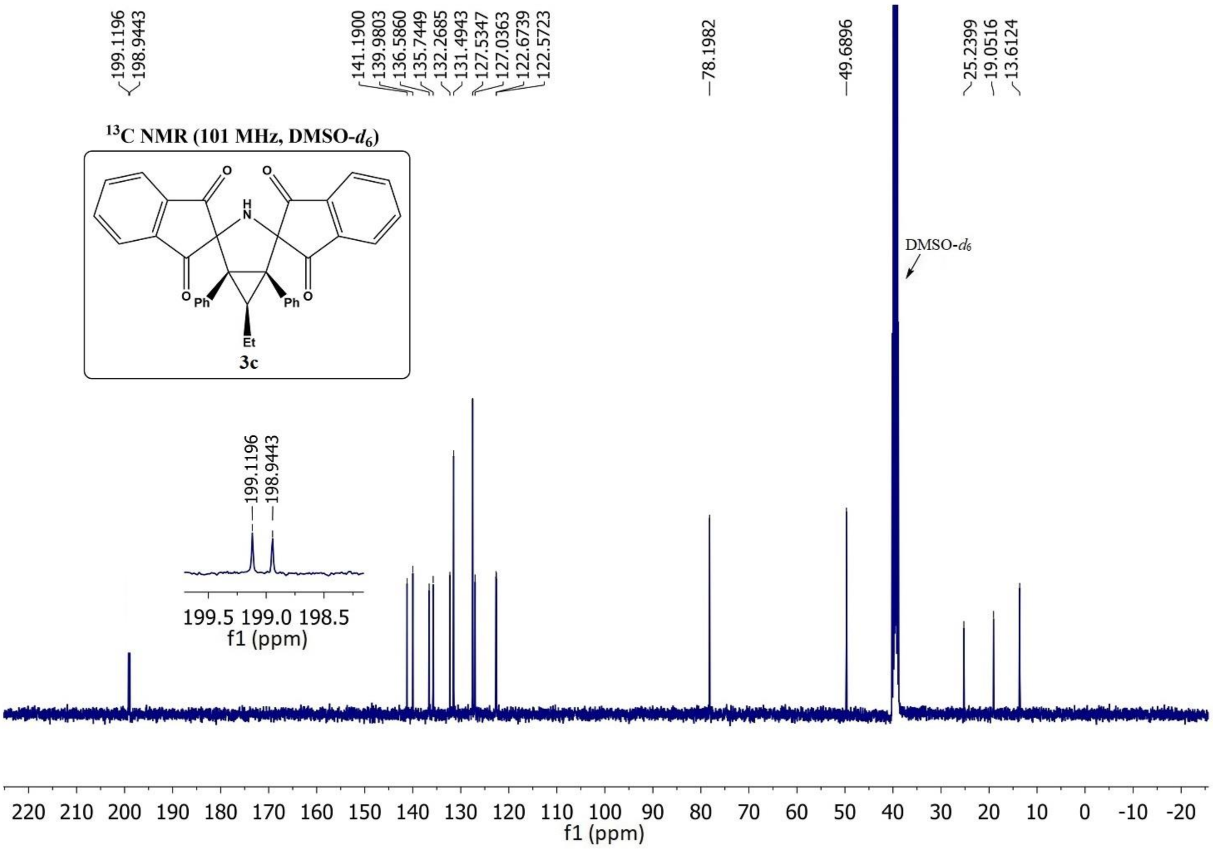

Figure S6: ^{13}C NMR spectrum of compound **3c** (101 MHz, DMSO-d_6)

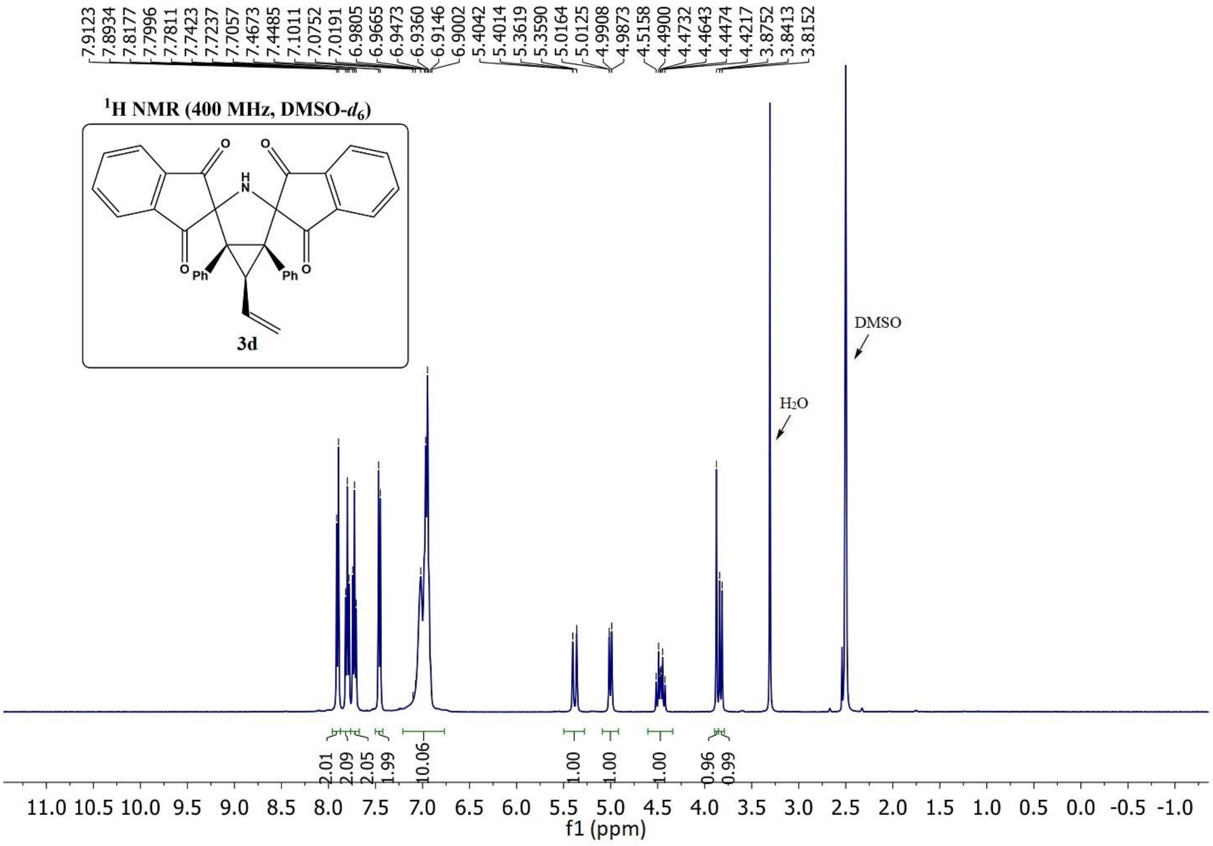

Figure S7: ^{1}H NMR spectrum of compound **3d** (400 MHz, DMSO-*d*$_6$)

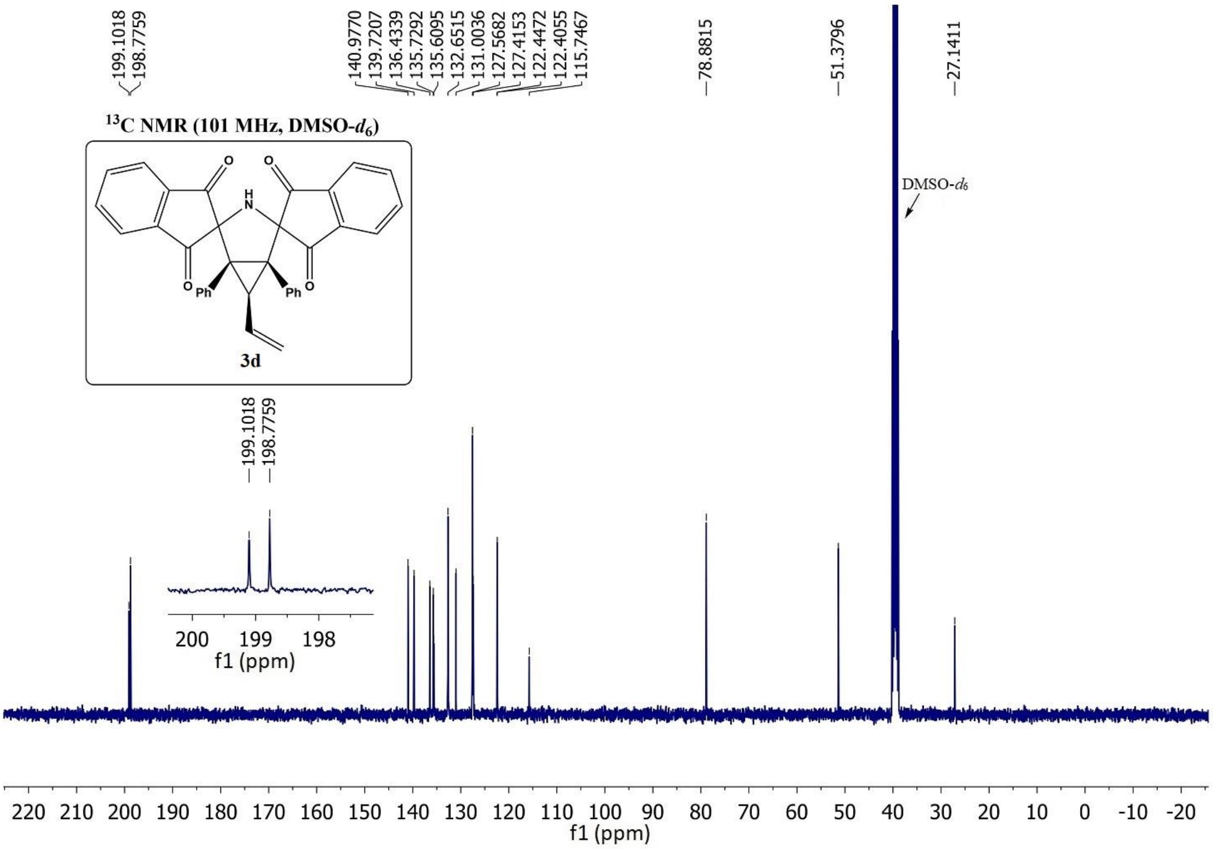

Figure S8: ^{13}C NMR spectrum of compound **3d** (101 MHz, DMSO-*d*$_6$)

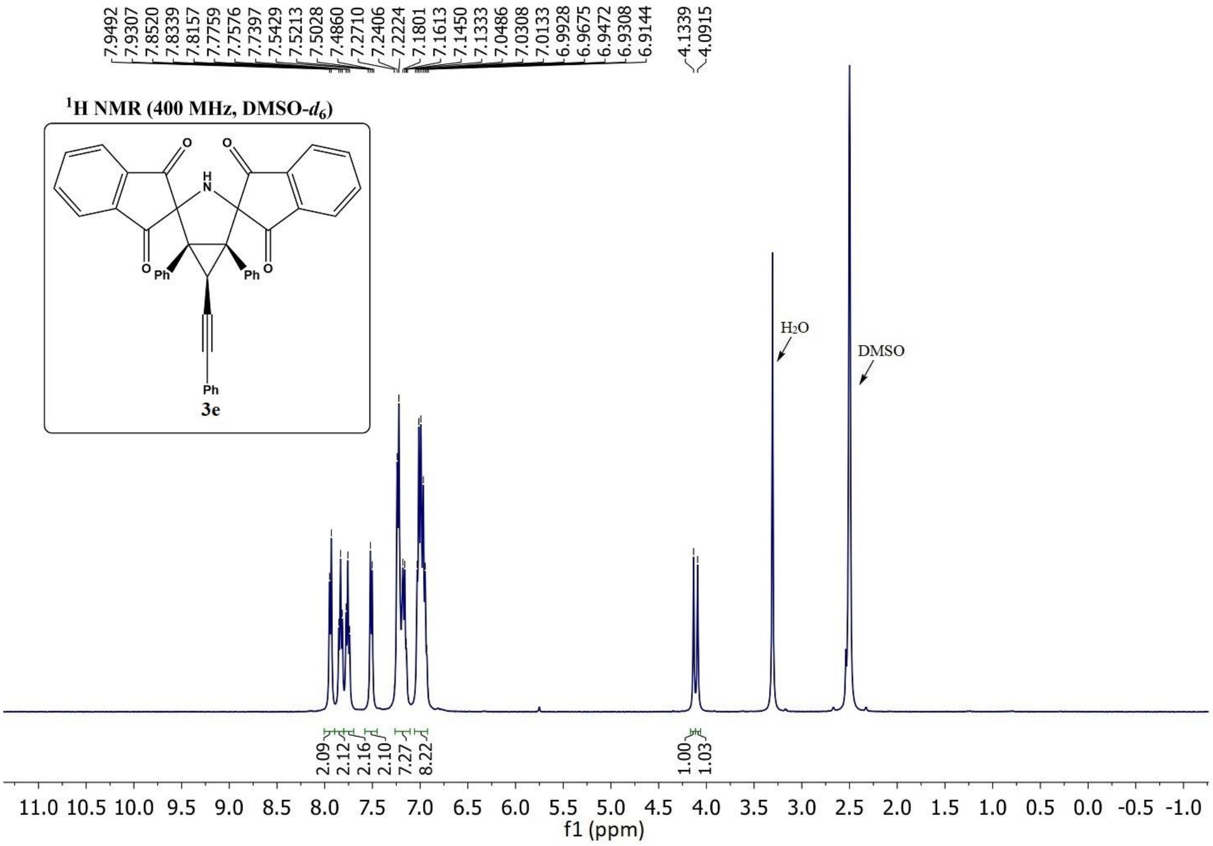

Figure S9: ^{1}H NMR spectrum of compound **3e** (400 MHz, DMSO-d_6)

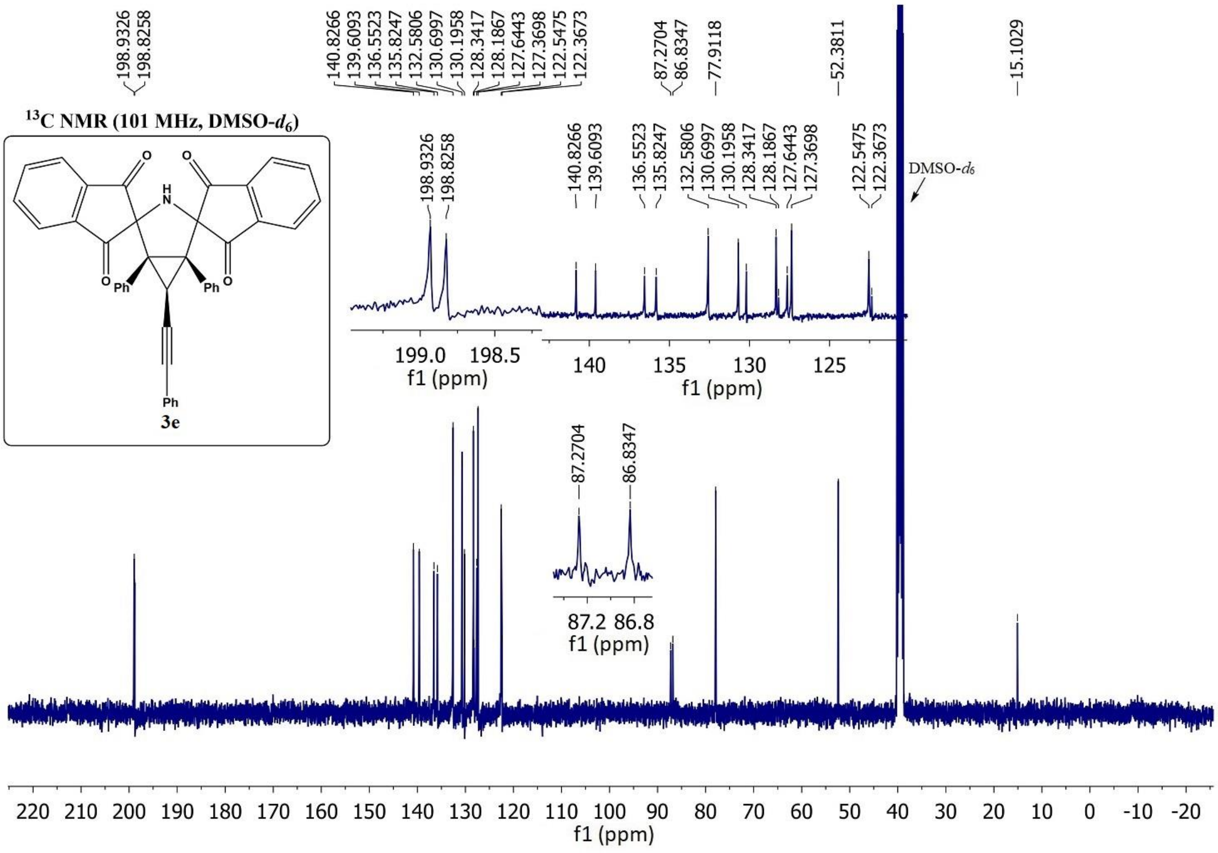

Figure S10: ^{13}C NMR spectrum of compound **3e** (101 MHz, DMSO-d_6)

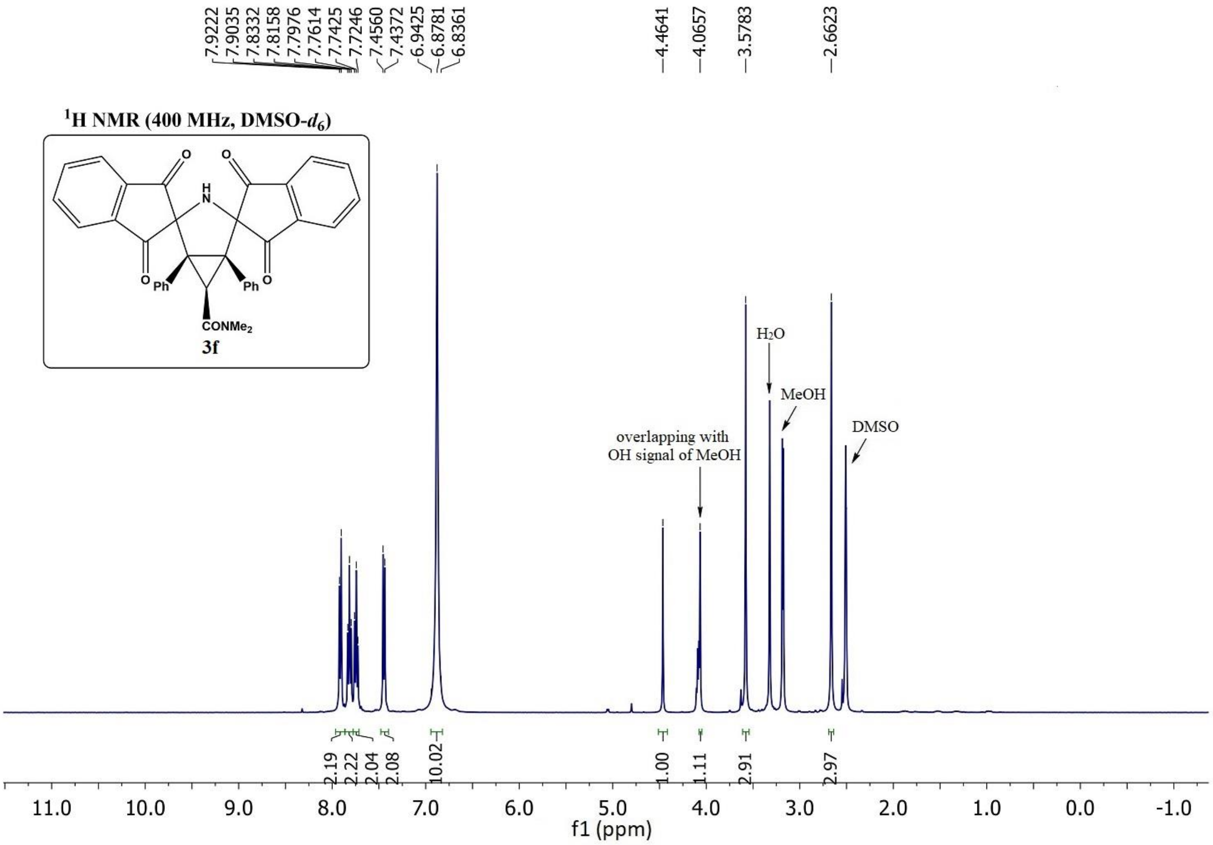

Figure S11: ^{1}H NMR spectrum of compound **3f** (400 MHz, DMSO-d_6)

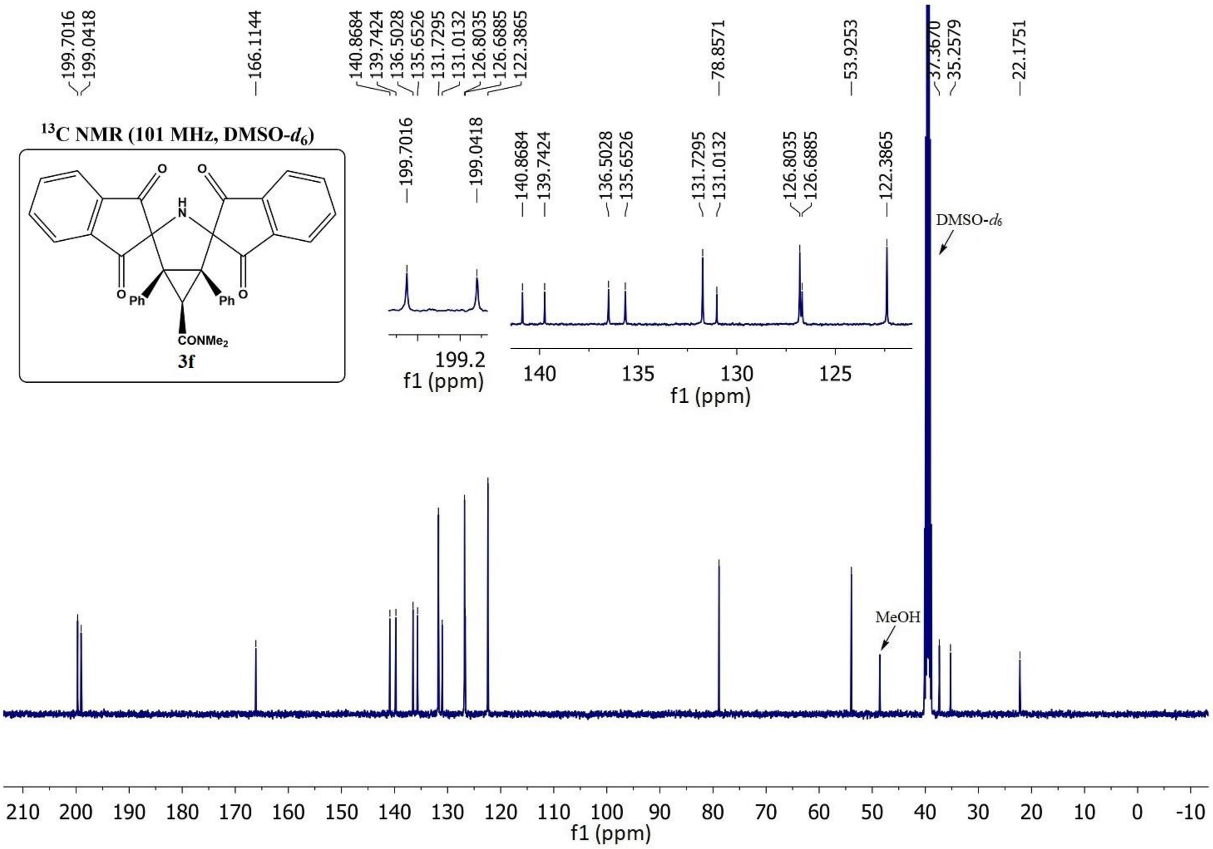

Figure S12: ^{13}C NMR spectrum of compound **3f** (101 MHz, DMSO-d_6)

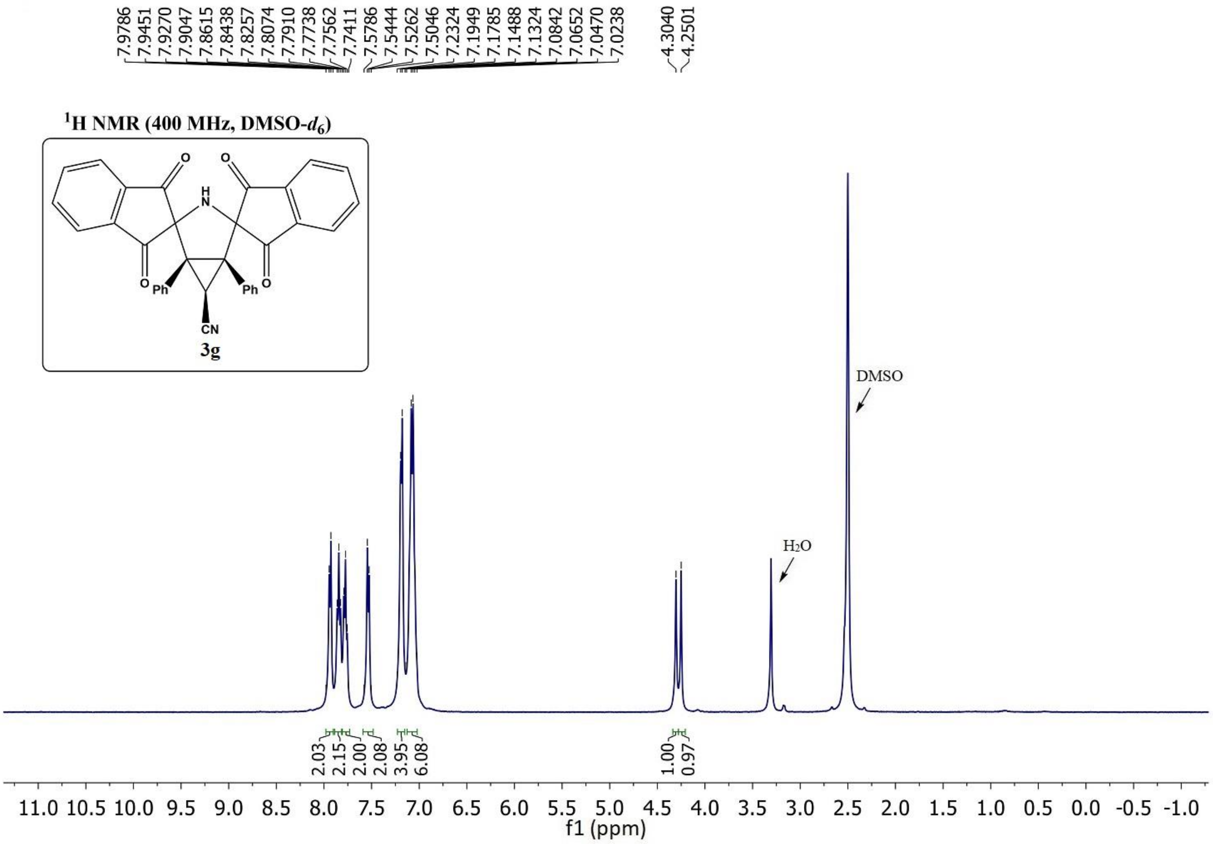

Figure S13: ^{1}H NMR spectrum of compound **3g** (400 MHz, DMSO-d_6)

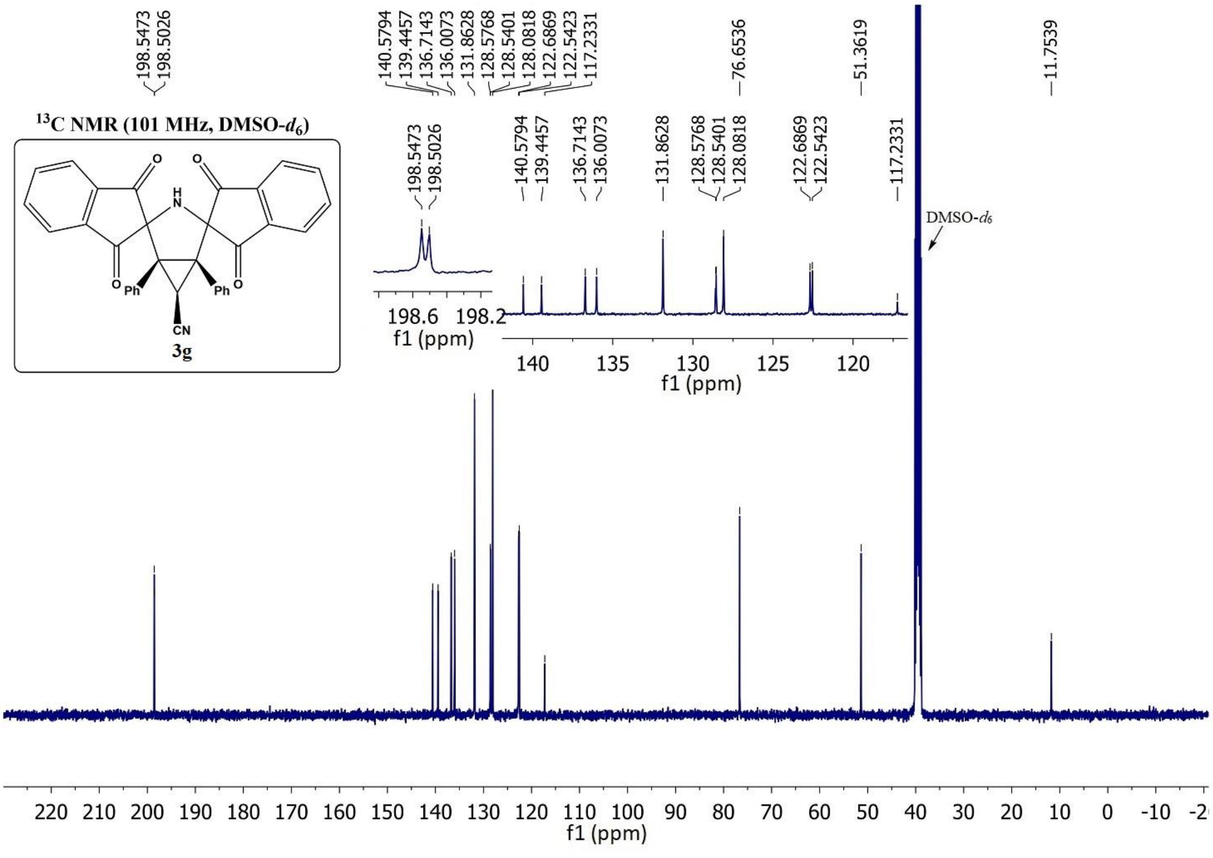

Figure S14: ^{13}C NMR spectrum of compound **3g** (101 MHz, DMSO-d_6)

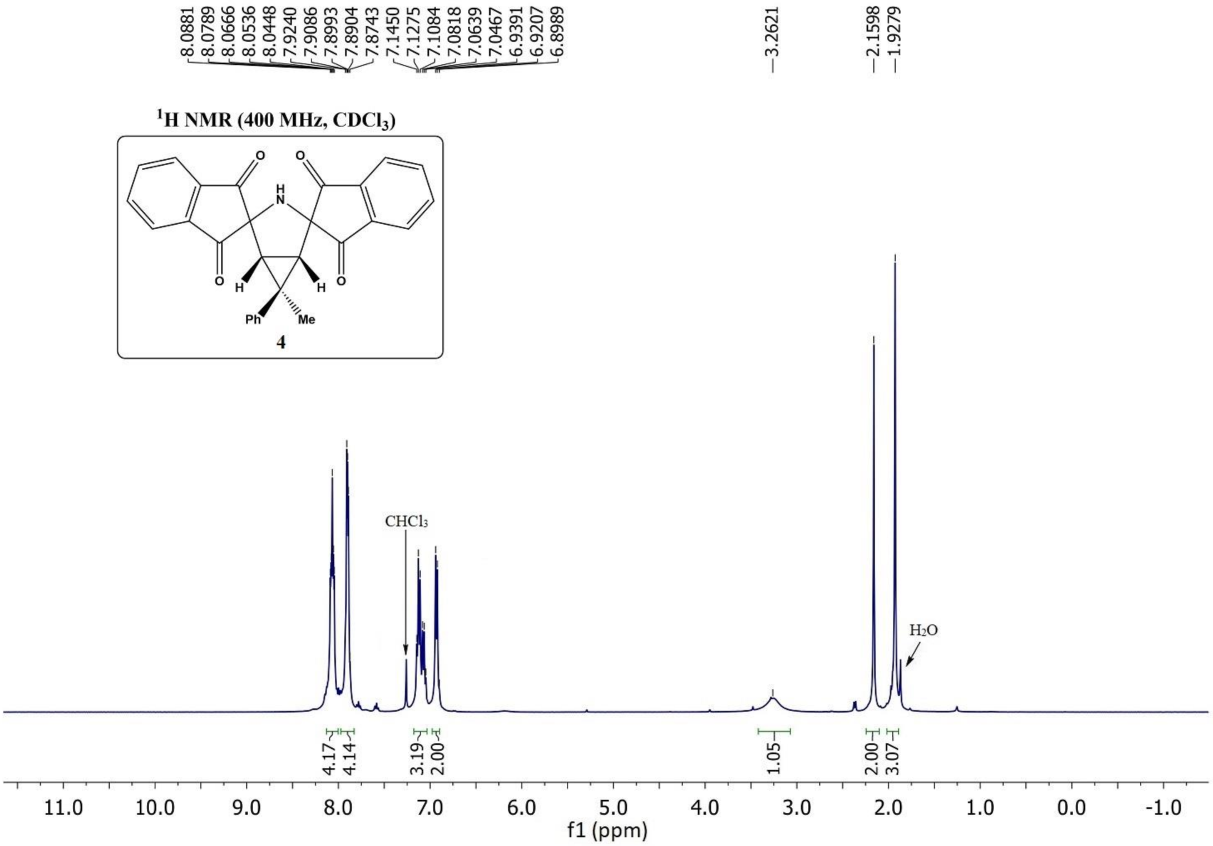

Figure S15: ^{1}H NMR spectrum of compound **4** (400 MHz, $CDCl_3$)

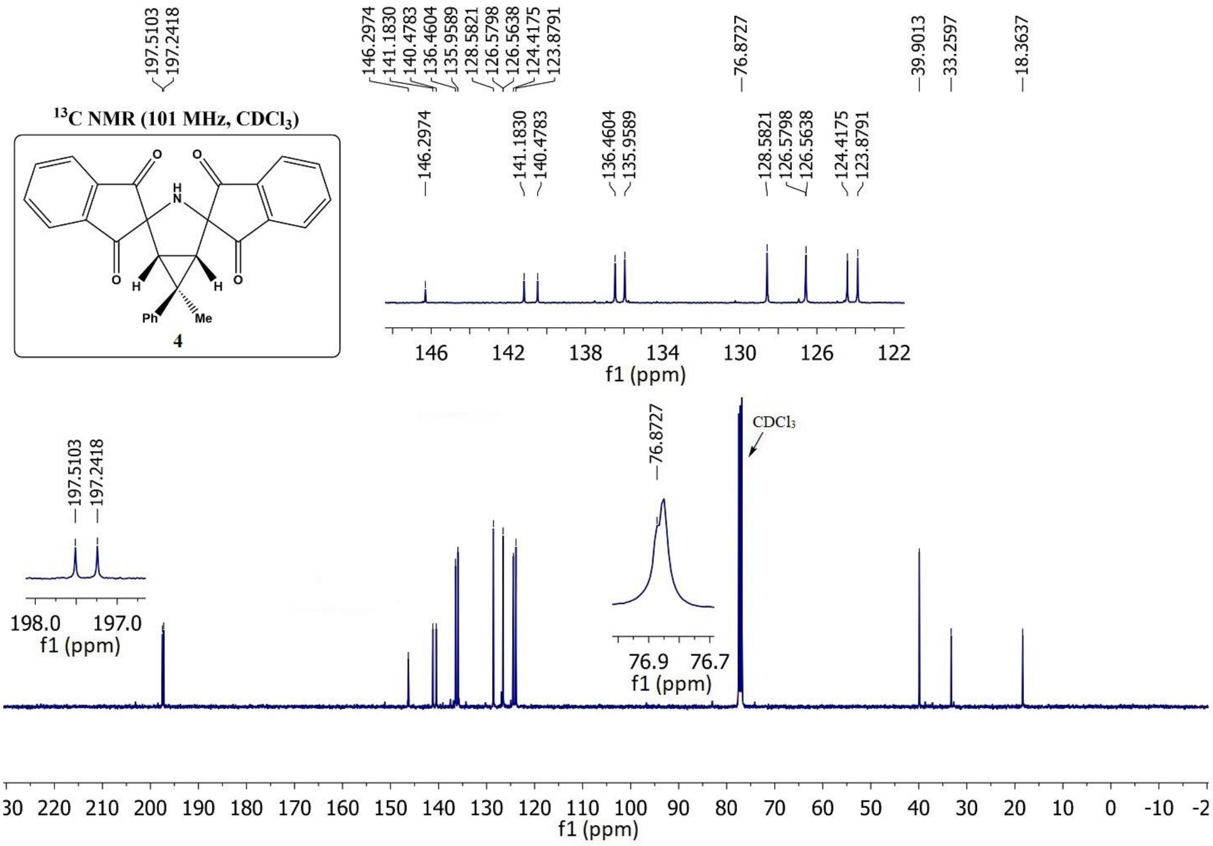

Figure S16: ^{13}C NMR spectrum of compound **4** (101 MHz, $CDCl_3$)

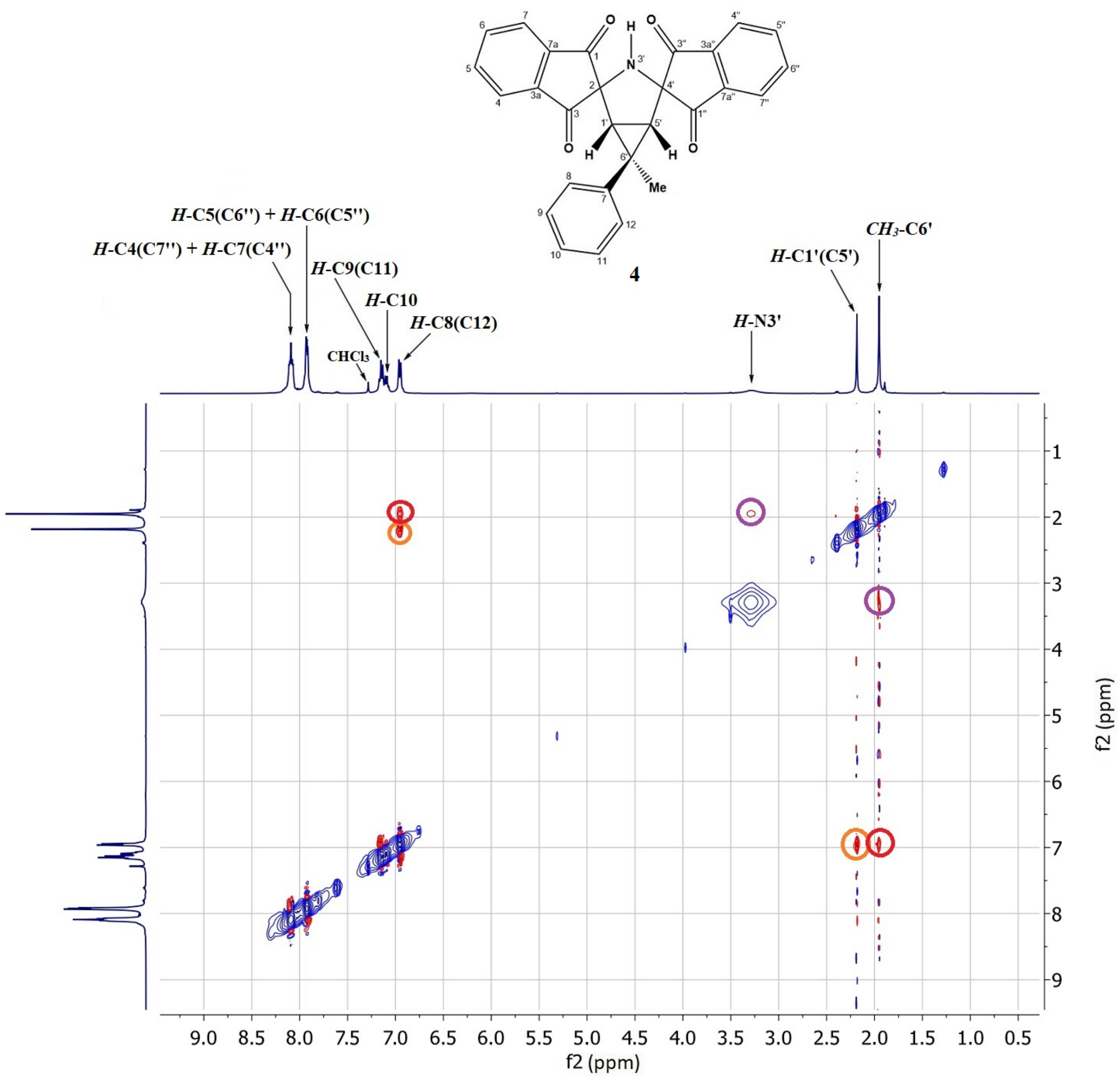

Figure S17: 2D ^{1}H-^{1}H NOESY spectrum of compound **4** (400 MHz, $CDCl_3$)

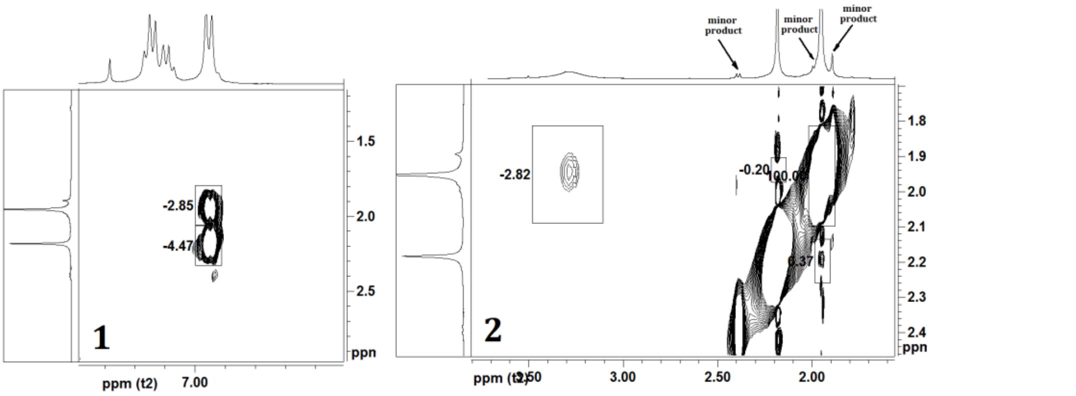

Figure S18: Fragment of 2D ^{1}H-^{1}H NOESY spectrum of compound **4**

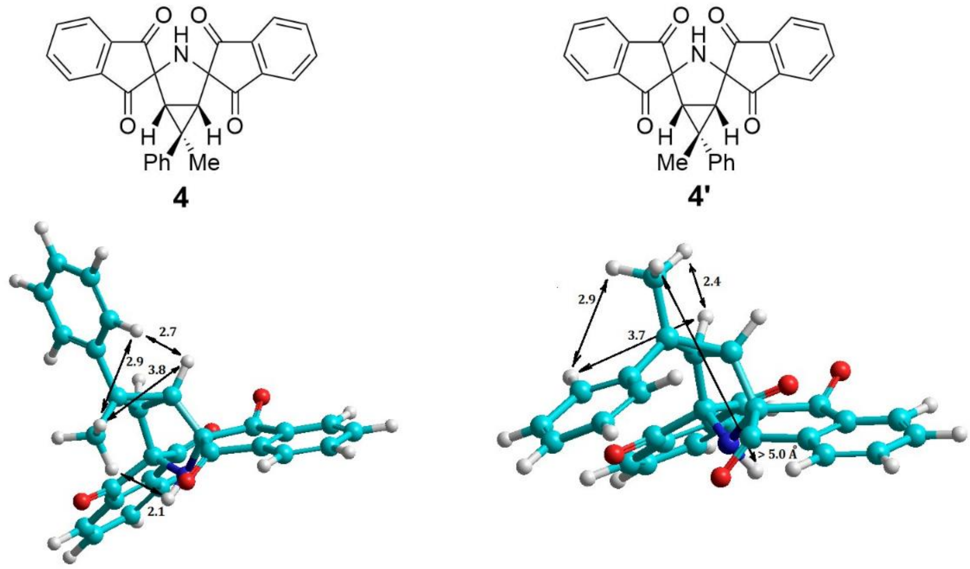

Figure S19: Spatial structure optimization of compounds **4** and **4'** by the molecular mechanic method (MM2)

Procedure for the assignment of the relative configuration for the cycloadduct derived from 3-methyl-3-phenylcyclopropene:

To determine the relative configuration of the cycloadduct derived from 3-methyl-3-phenylcyclopropene, we carried out a quantitative analysis of its two-dimensional (2D) NMR spectrum (^{1}H–^{1}H nuclear Overhauser effect spectroscopy (NOESY)).

A choice was made between two possible configurations (structures **4** and **4'**) by a comparison of interproton distances with corresponding cross-peak intensities.

The distance 2.9 Å between *H*-C8 and protons of a methyl group ($r_{H\text{-C8 - Me}}$) was used as a reference; this value is almost the same for both structures **4** and **4'**.

According to the well-known relationship S_{AB} / S_{AC} = (r_{AC} / r_{AB}) ^{6} , the values $S_{H\text{-C1-}H\text{-C8}}$ / $S_{H\text{-C1 - Me}}$ for structures **4** and **4'** must be equal (3.8 Å/2.7 Å) = 7.8 and (2.4 Å/3.7 Å) = 0.075, correspondingly. Integration of these cross peaks (Figure S18) provides an unambiguous answer since there is no a cross-peak between *H*-C1(5) and the Me group in the spectrum at all (Figure S17).

Furthermore, using value $S_{H\text{-C1 – }H\text{-C8}}$ / $S_{H\text{-C8 - Me}}$ and interproton distance $r_{\text{H8-Me}}$ as a reference, it is possible to calculate the distance between *H*-C8 and protons of the methyl group ($r_{H\text{-C8 - Me}}$ = 2.7 * (4.47 / 2.85)$^{1/6}$ = 2.91 Å). This value corresponds well to the calculated value for diastereomer **4**.

Finally, the distance between the methyl group and NH proton in diastereomer **4'** is more than 5.0 Å, while there is a quite intensive cross-peak (2.82) which corresponds to the distance less than 3.0 Å.

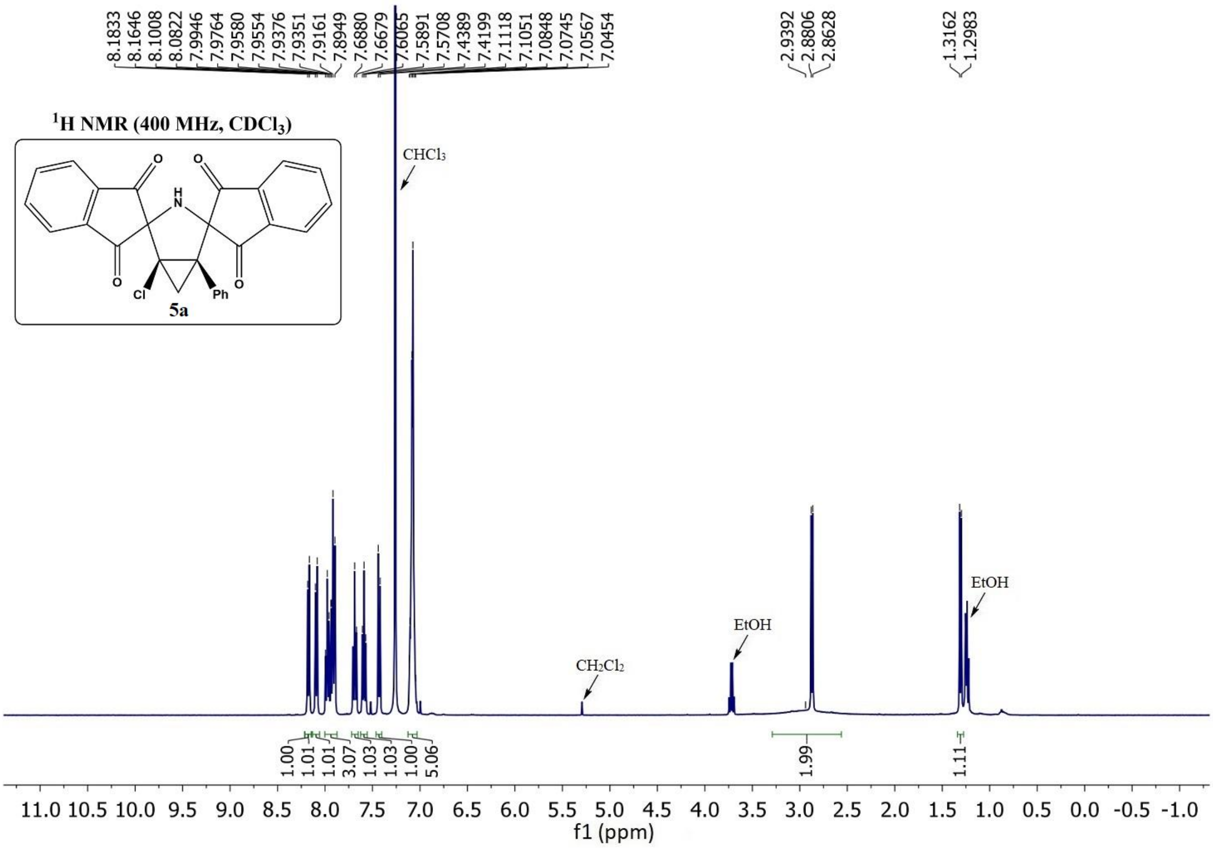

Figure S20: ^{1}H NMR spectrum of compound **5a** (400 MHz, $CDCl_3$)

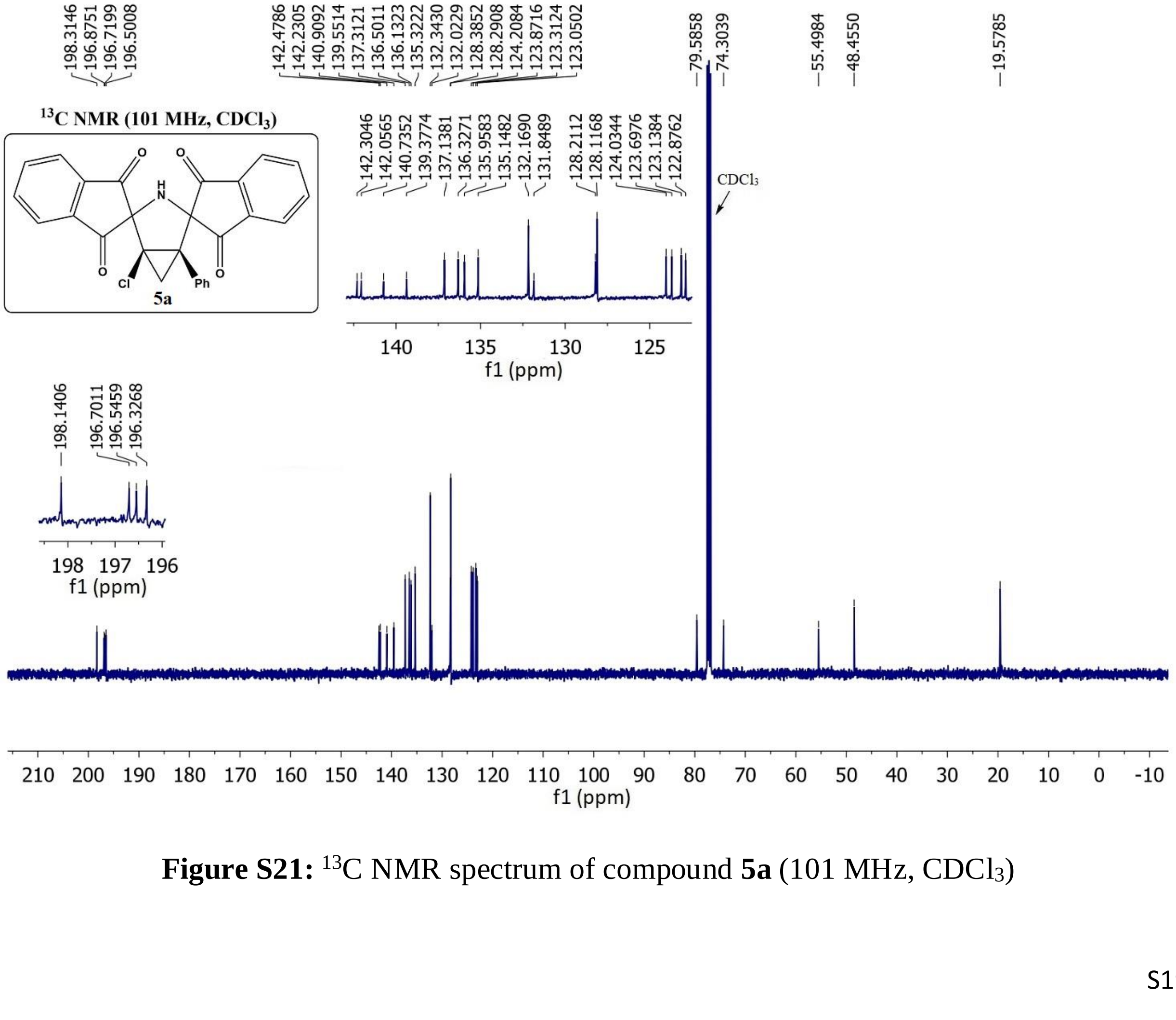

Figure S21: ^{13}C NMR spectrum of compound **5a** (101 MHz, $CDCl_3$)

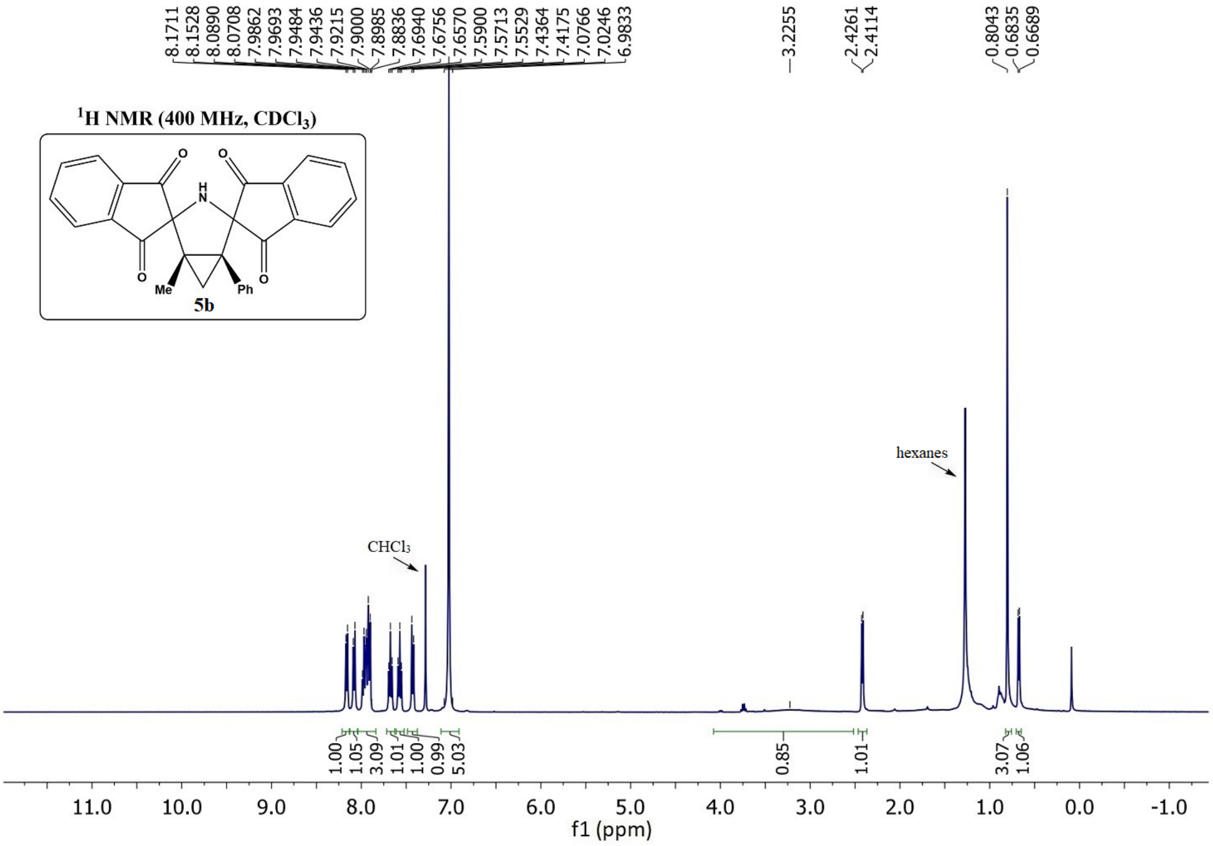

Figure S22: ^{1}H NMR spectrum of compound **5b** (400 MHz, $CDCl_3$)

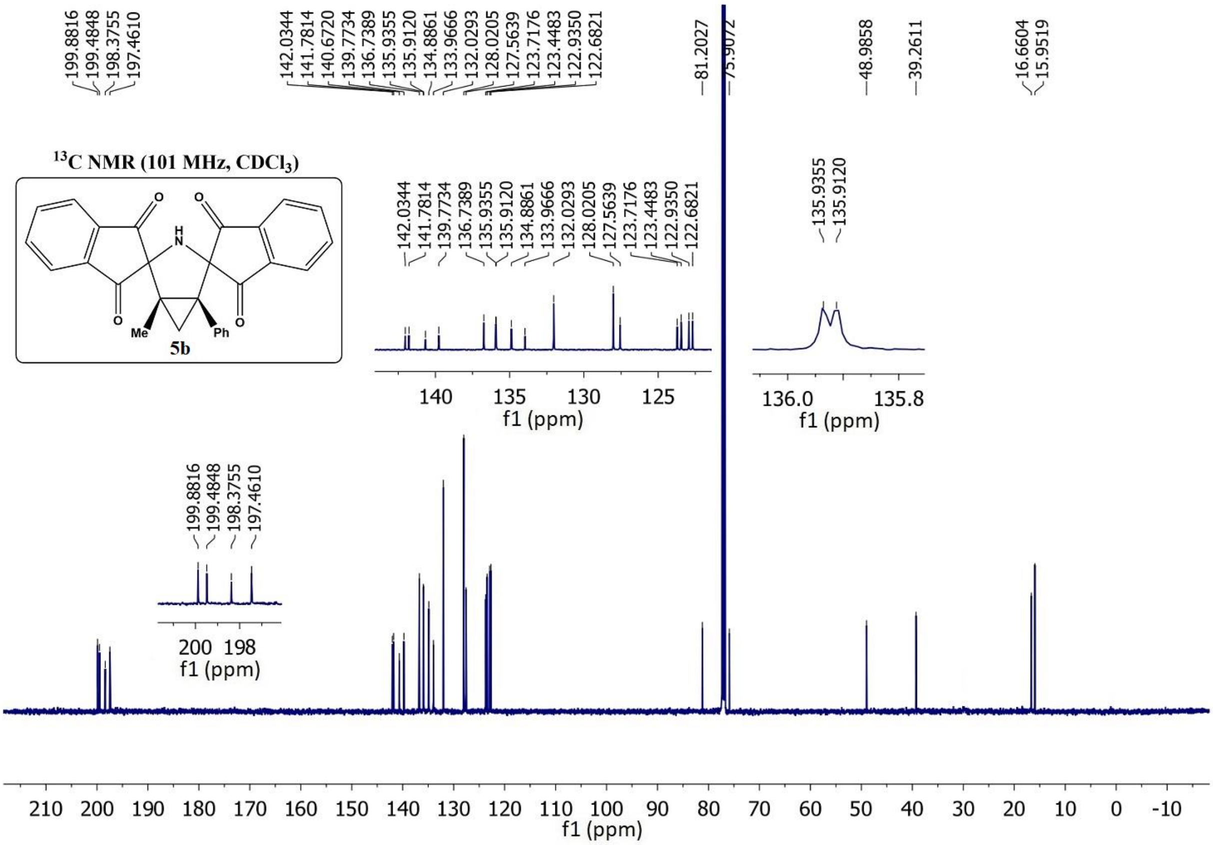

Figure S23: ^{13}C NMR spectrum of compound **5b** (101 MHz, $CDCl_3$)

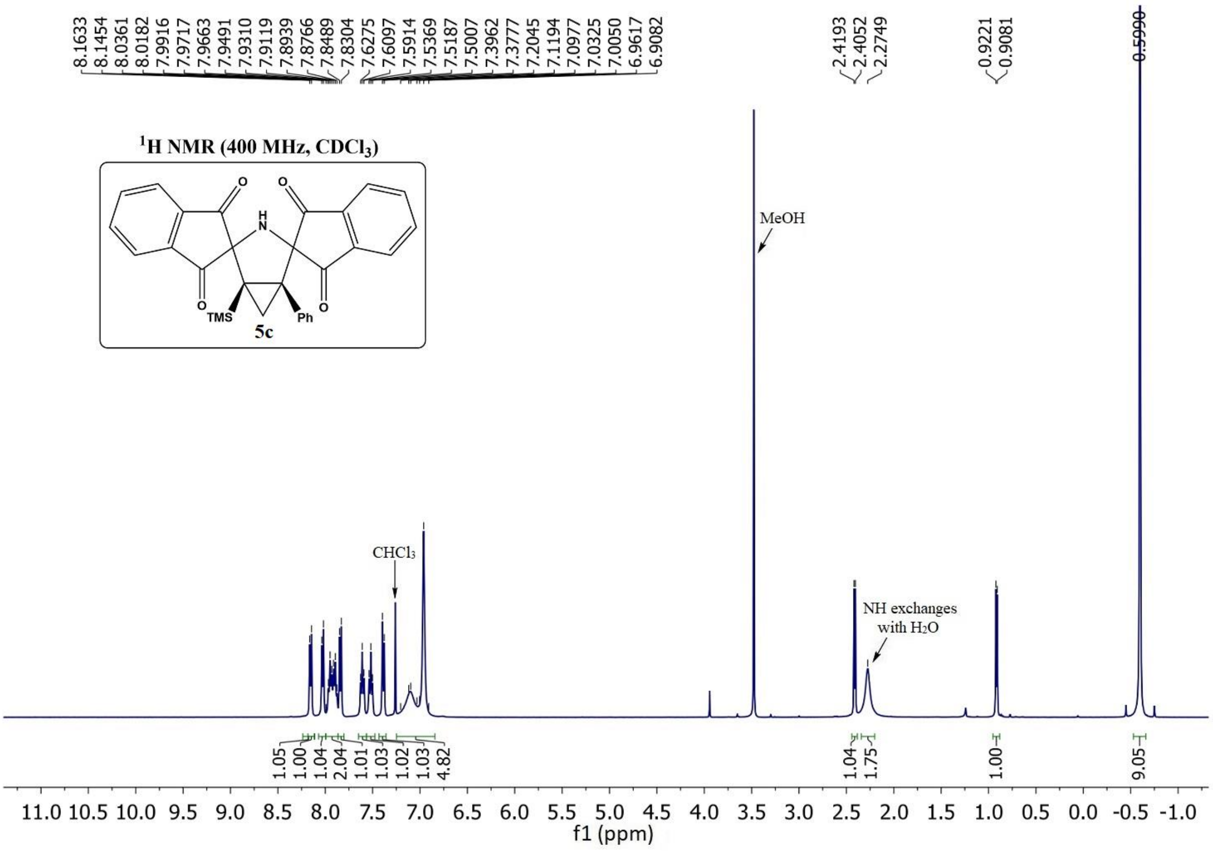

Figure S24: ^{1}H NMR spectrum of compound **5c** (400 MHz, $CDCl_3$)

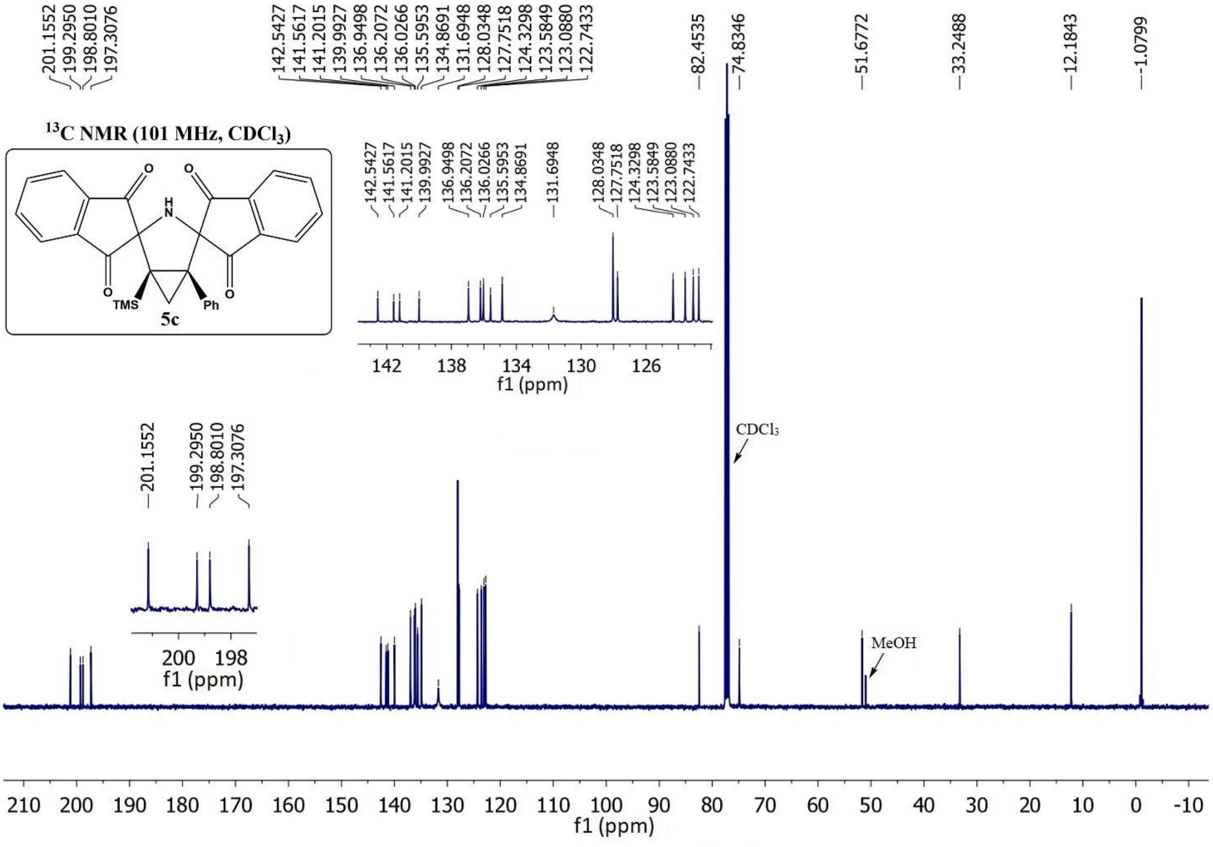

Figure S25: ^{13}C NMR spectrum of compound **5c** (101 MHz, $CDCl_3$)

4. X-ray data for compounds 3b and 3e

General procedure of the sample preparation and crystal structure determination: Single crystals of compounds **3b** and **3e** were grown by slow evaporation of their solutions in an ethanol–chloroform mixture at room temperature. For single crystal X-ray diffraction experiments crystals were fixed on a micro mount and placed on at SuperNova, single source at offset/far, HyPix3000 or Xcalibur Eos diffractometers and were measured at 100 K using monochromated MoKα (**3b**) and CuKα (**3e**) radiations, respectively. The structures were solved by the ShelXT1 structure solution program using Intrinsic Phasing and the Superflip2 structure solution program using Charge Flipping and refined by means of the SHELXL program3 incorporated in the OLEX2 program package. Empirical absorption correction was applied in CrysAlisPro program complex using spherical harmonics, implemented in SCALE3 ABSPACK scaling algorithm. The crystallographic data and some parameters of refinement are collected in Tables S1 and S2. Crystallographic data for compounds **3b** and **3e** have been deposited at the Cambridge Crystallographic Data Centre (Deposition nos. CCDC 2055282 (**3b**) and CCDC 2055281 (**3e**)) and can be obtained free of charge via www.ccdc.cam.ac.uk/data_request/cif.

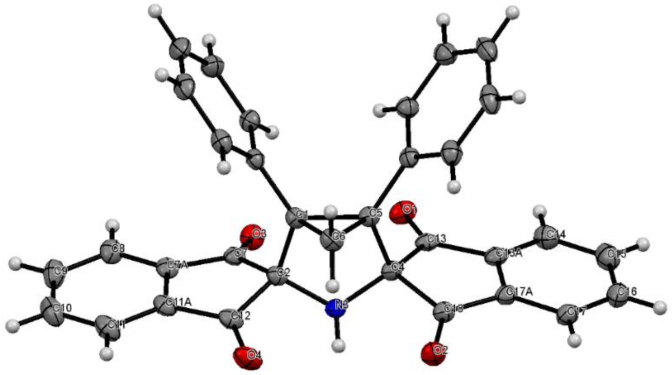

Figure S26: ORTEP representation of the molecular structure of **3b** (CCDC 2055282). Thermal ellipsoids are drawn at 50% probability level.

Table S1: Crystal data and structure refinement for compound **3b**

Empirical formula	$C_{33}H_{21}NO_4$
Formula weight	495.51
Temperature/K	100(2)
Crystal system	monoclinic
Space group	$P2_1/c$
a/Å	13.6792(4)
b/Å	10.3557(3)
c/Å	17.7456(5)
α/°	90
β/°	105.580(3)
γ/°	90

Volume/Å^3	2421.43(13)
Z	4
ρ_{calc}g/cm^3	1.359
μ/mm^{-1}	0.090
F(000)	1032.0
Crystal size/mm^3	0.54 × 0.5 × 0.1
Radiation	MoKα (λ = 0.71073)
2Θ range for data collection/°	5.172 to 61.946
Index ranges	$-19 \leq h \leq 16$, $-14 \leq k \leq 14$, $-23 \leq l \leq 24$
Reflections collected	28061
Independent reflections	7059 [R_{int} = 0.0356, R_{sigma} = 0.0419]
Data/restraints/parameters	7059/0/346
Goodness-of-fit on F^2	1.060
Final R indexes [I>=2σ (I)]	R_1 = 0.0514, wR_2 = 0.1163
Final R indexes [all data]	R_1 = 0.0711, wR_2 = 0.1256
Largest diff. peak/hole / e Å^{-3}	0.40/-0.25

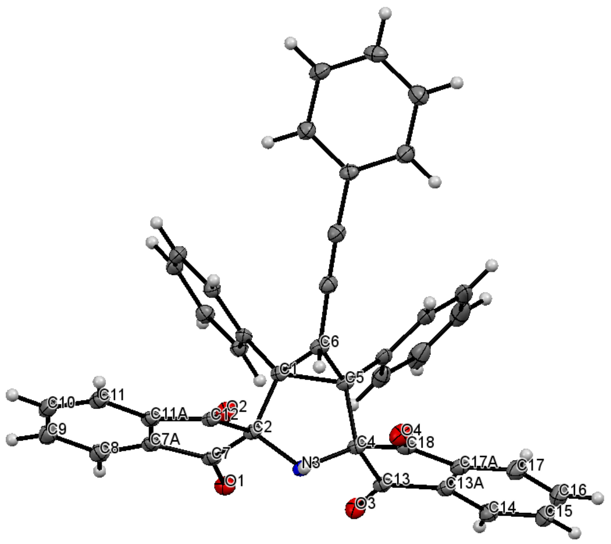

Figure S27: ORTEP representation of the molecular structure of **3e** (CCDC 2055281). Thermal ellipsoids are drawn at 50% probability level.

Table S2: Crystal data and structure refinement for compound **3e**

Empirical formula	$C_{41}H_{25}NO_4$
Formula weight	595.62
Temperature/K	100(2)
Crystal system	monoclinic
Space group	I2/a
a/Å	26.0260(2)
b/Å	10.16116(8)
c/Å	22.56815(19)
α/°	90
β/°	98.6498(8)

γ/°	90
Volume/Å^3	5900.37(9)
Z	8
ρ_{calc}g/cm^3	1.341
μ/mm^{-1}	0.690
F(000)	2480.0
Crystal size/mm^3	0.33 × 0.24 × 0.19
Radiation	CuKα (λ = 1.54184)
2Θ range for data collection/°	6.87 to 140.726
Index ranges	$-31 \le h \le 31$, $-12 \le k \le 12$, $-27 \le l \le 25$
Reflections collected	20037
Independent reflections	5600 [R_{int} = 0.0248, R_{sigma} = 0.0172]
Data/restraints/parameters	5600/0/422
Goodness-of-fit on F^2	1.044
Final R indexes [I>=2σ (I)]	R_1 = 0.0367, wR_2 = 0.0907
Final R indexes [all data]	R_1 = 0.0382, wR_2 = 0.0919
Largest diff. peak/hole / e Å^{-3}	0.21/-0.24

5. Calculation details

Computational methodology: The full geometry optimization of reactants, products, and transition state structures (TSs) were carried out at the DFT/HF level of theory using M11 hybrid exchange-correlation functional [15] and the cc-pVDZ basis set [16]. The polarizable continuum model (PCM) was used to calculate solvent effects of water and tetrahydrofuran [17]. The optimizations were performed using the Berny analytical gradient optimization method [18]. All stationary points were described by harmonic vibrational frequency calculations to prove the location of correct minima (only real frequencies) and transition states (only one imaginary frequency). For the transition states, the normal modes corresponding to the imaginary frequencies were related to the vibrations of new developing bonds. IRC calculations were conducted to check the energy profiles connecting each TS to the two associated minima of the proposed mechanism [19]. Due to the poor estimation of the Kohn–Sham orbitals for FMO energy values, HOMO and LUMO energies and the corresponding global descriptors for reactants were computed by using HF/6-311g single-point calculation based on the M11/cc-pVDZ optimized geometries. Thermal corrections to enthalpy and entropy values were evaluated at 298.15 K and 1.0 atm. All calculations were performed using the Gaussian 09 computational program package [20].

Table S3: Energies (a.u.) and Cartesian coordinates of stationary points for reactants, intermediates, products, and transition states (M11/cc-pVDZ, PCM = H_2O or THF).

Ruhemann's Purple, PCM = H_2O

E_0 = -1045.828195
E (298 K) = -1045.811289
H (298 K) = -1045.810345
G (298 K) = -1045.874024
Imaginary frequencies = 0

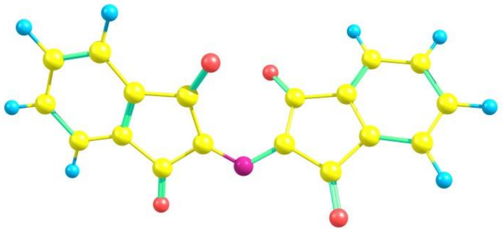

Cartesian coordinates:

C	5.748318	-0.413646	-0.168347
C	5.246647	-1.608523	0.363131
C	4.892267	0.651042	-0.474584
C	3.534700	0.477130	-0.242165
C	3.034851	-0.714378	0.283642
C	3.876641	-1.770354	0.603422
H	5.939941	-2.426232	0.595195
H	3.472288	-2.698520	1.025609
H	6.826665	-0.312708	-0.341396
H	5.273110	1.596358	-0.880321
C	2.395511	1.440428	-0.441844

N-Protonated Ruhemann's Purple (**1**), PCM = H_2O

E_0 = -1046.267172
E (298 K) = -1046.250052
H (298 K) = -1046.249107
G (298 K) = -1046.313363
Imaginary frequencies = 0

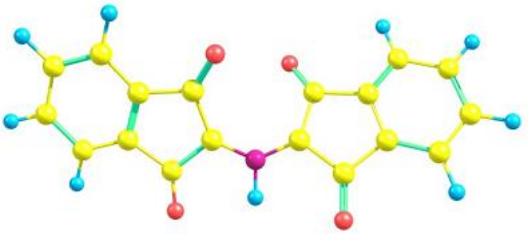

Cartesian coordinates:

C	5.845679	-0.206518	-0.117107
C	5.441902	-1.477441	0.312961
C	4.908349	0.799478	-0.370481
C	3.567015	0.488329	-0.190118
C	3.162825	-0.782872	0.234132
C	4.089416	-1.780433	0.501998
H	6.201143	-2.244356	0.506792
H	3.763553	-2.769007	0.846174
H	6.913699	0.001013	-0.251667
H	5.214381	1.800581	-0.696015

C	1.536326	-0.613870	0.439793
O	0.851003	-1.432104	1.032399
O	2.510262	2.592112	-0.829282
C	1.158108	0.704170	-0.102181
N	-0.007882	1.329497	-0.053956
C	-1.168299	0.696037	0.014321
C	-2.413545	1.431780	0.325422
C	-1.532990	-0.642128	-0.485819
C	-3.030447	-0.752969	-0.326774
C	-3.542769	0.450897	0.157299
C	-4.902238	0.619240	0.382503
C	-5.747248	-0.464141	0.113459
C	-5.233012	-1.671768	-0.375504
C	-3.861325	-1.827894	-0.609805
H	-3.447584	-2.766103	-0.999390
H	-6.826705	-0.368193	0.282410
H	-5.917736	-2.504179	-0.579028
H	-5.292785	1.574273	0.754825
O	-0.839365	-1.469029	-1.056215
O	-2.540530	2.594857	0.672629

C	2.361427	1.356728	-0.353057
C	1.664786	-0.837067	0.383145
O	1.018398	-1.722502	0.902288
O	2.300528	2.541881	-0.618586
C	1.203003	0.479067	-0.093971
N	-0.006301	1.012985	-0.054029
C	-1.210672	0.469978	0.003990
C	-2.377331	1.344765	0.235574
C	-1.660392	-0.865968	-0.427588
C	-3.158529	-0.821028	-0.277988
C	-3.574678	0.459805	0.103463
C	-4.918745	0.763228	0.276847
C	-5.846213	-0.260644	0.061385
C	-5.430331	-1.541394	-0.325864
C	-4.075303	-1.836764	-0.508547
H	-3.740124	-2.833228	-0.819438
H	-6.915949	-0.059619	0.192160
H	-6.182078	-2.322222	-0.490869
H	-5.234156	1.771725	0.568902
O	-1.006249	-1.762166	-0.917419
O	-2.327668	2.538447	0.462118
H	-0.010879	2.052777	-0.071387

O-protonated Ruhemann's Purple (**1'**), PCM = H_2O

E_0 = -1046.241647
E (298 K) = -1046.224302
H (298 K) = -1046.223358
G (298 K) = -1046.288406
Imaginary frequencies = 0

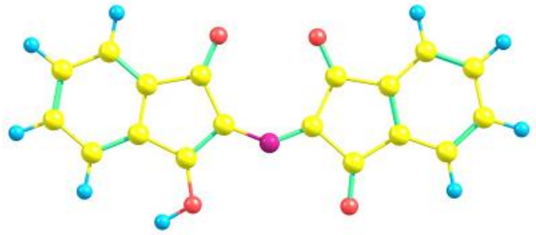

Cartesian coordinates:

C	0.464947	0.846365	0.000003
C	0.789121	-0.511702	-0.000020
C	1.463351	1.831576	0.000019
C	2.783233	1.403027	0.000011
C	3.104977	0.043726	-0.000011
C	2.128198	-0.932351	-0.000027
H	-0.015392	-1.256682	-0.000032
H	2.400724	-1.994479	-0.000046
H	-0.588055	1.150722	0.000008
H	1.186246	2.893070	0.000037
C	4.061094	2.145361	0.000014
C	4.608346	-0.115739	-0.000022
O	5.170173	-1.184664	-0.000049
O	4.165997	3.449561	0.000028
N	6.386988	1.871353	-0.000003
C	7.606605	1.489518	0.000007

C-protonated Ruhemann's Purple (**1''**), PCM = H_2O

E_0 = -1046.261422
E (298 K) = -1046.244161
H (298 K) = -1046.243217
G (298 K) = -1046.308217
Imaginary frequencies = 0

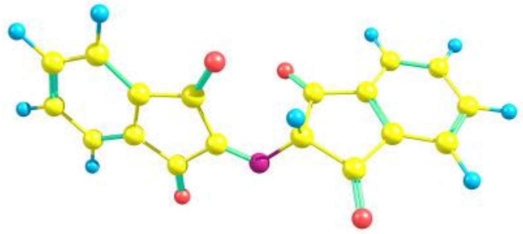

Cartesian coordinates:

C	-3.830346	-0.521810	0.646660
C	-3.254693	-1.640065	0.020375
C	-3.065172	0.599746	0.963698
C	-1.711101	0.571304	0.640862
C	-1.138297	-0.543016	0.017389
C	-1.899478	-1.663863	-0.304639
H	-3.884934	-2.505673	-0.214966
H	-1.437767	-2.528910	-0.794670
H	-4.899796	-0.535250	0.887713
H	-3.504062	1.478871	1.449560
C	-0.685441	1.626836	0.863802
C	0.313661	-0.325548	-0.226579
C	0.627049	1.056449	0.339538
O	-0.838490	2.716243	1.361487
O	1.077206	-1.068888	-0.800141
N	1.704415	1.722107	0.371351

C	8.672574	2.571720	-0.000014
C	8.344746	0.159442	0.000041
C	9.801668	0.502630	0.000019
C	9.994548	1.883738	-0.000012
C	11.272608	2.436214	-0.000031
C	12.356708	1.558283	-0.000019
C	12.162118	0.167047	0.000011
C	10.878521	-0.379403	0.000031
H	10.709269	-1.462820	0.000056
H	11.411729	3.523730	-0.000053
H	13.378663	1.955578	-0.000033
H	13.035787	-0.495508	0.000020
O	7.906034	-0.966152	0.000074
O	8.478824	3.767409	-0.000040
C	5.159064	1.287060	0.000002
H	3.296707	3.884101	0.000032

C	2.915281	1.073375	-0.057017
C	4.093842	2.030867	-0.261278
C	3.446817	0.070281	0.996152
H	2.787616	0.475201	-0.983059
O	2.760065	-0.666132	1.657320
O	4.042356	3.117685	-0.784160
C	5.301347	1.368758	0.314916
C	4.926187	0.236224	1.039822
C	6.633852	1.763653	0.229958
C	5.866820	-0.543748	1.708821
C	7.203443	-0.158123	1.617739
C	7.582116	0.981225	0.888116
H	8.641848	1.258967	0.841922
H	6.916605	2.659316	-0.335296
H	5.558593	-1.425672	2.282171
H	7.974718	-0.748215	2.126630

Hydrogen chloride, PCM = H_2O

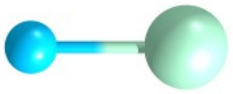

E_0 = -460.777622
E (298 K) = -460.775261
H (298 K) = -460.774317
G (298 K) = -460.795528
Imaginary frequencies = 0

Cl	-3.515911	-0.411782	0.000000
H	-2.216878	-0.411782	0.000000

Chloride ion, PCM = H_2O

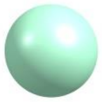

E_0 = -460.348937
E (298 K) = -460.347520
H (298 K) = -460.346576
G (298 K) = -460.363959
Imaginary frequencies = 0

Cl	0.000000	0.000000	0.000000

N-Protonated Ruhemann's Purple (**1**), PCM = THF

E_0 = -1046.263857
E (298 K) = -1046.246762
H (298 K) = -1046.245818
G (298 K) = -1046.310010
Imaginary frequencies = 0

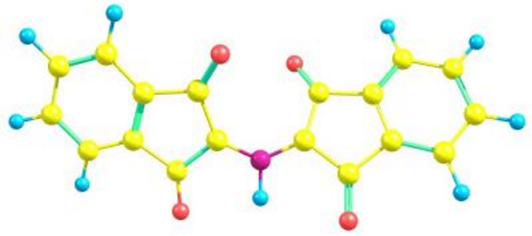

Cartesian coordinates:

C	5.843497	-0.208940	-0.116265
C	5.437647	-1.481264	0.306978
C	4.907846	0.799728	-0.363652
C	3.566325	0.489911	-0.184790
C	3.160392	-0.782227	0.233753
C	4.085107	-1.782609	0.495715
H	6.195658	-2.250695	0.495638
H	3.755623	-2.771558	0.835334
H	6.911883	-0.002873	-0.250178
H	5.213300	1.802575	-0.684274

1,2,3-Triphenylcyclopropene (**2a**), PCM = THF

E_0 = -809.038794
E (298 K) = -809.022008
H (298 K) = -809.021064
G (298 K) = -809.087218
Imaginary frequencies = 0

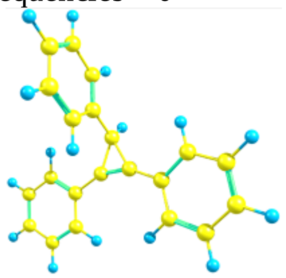

Cartesian coordinates:

C	-0.198883	-0.721064	-0.682374
C	0.779691	0.270271	-1.263840
C	1.062981	-1.056400	-0.601530
C	1.079236	1.558192	-0.549564
H	0.870959	0.346013	-2.362726
C	1.461767	2.691855	-1.275745
C	0.985517	1.654799	0.845304
C	1.268098	2.855115	1.495354
C	1.745585	3.894627	-0.627314
C	1.649993	3.980947	0.761980

C	2.362242	1.360451	-0.346617
C	1.662072	-0.835219	0.383090
O	1.014612	-1.719154	0.901685
O	2.303127	2.545878	-0.610938
C	1.203037	0.483319	-0.091513
N	-0.006351	1.017049	-0.054653
C	-1.210337	0.472832	0.000681
C	-2.377484	1.347009	0.228513
C	-1.657708	-0.865376	-0.428223
C	-3.156132	-0.821525	-0.276836
C	-3.573461	0.460090	0.099323
C	-4.917499	0.762782	0.271398
C	-5.843849	-0.262803	0.061266
C	-5.426599	-1.544764	-0.319673
C	-4.071657	-1.839186	-0.501977
H	-3.733336	-2.835857	-0.808712
H	-6.913850	-0.062674	0.191255
H	-6.177494	-2.327550	-0.479706
H	-5.231926	1.772919	0.558729
O	-1.003474	-1.760385	-0.918505
O	-2.329035	2.541195	0.452604
H	-0.011670	2.056479	-0.071654

H	1.872586	4.924842	1.274084
H	1.189528	2.913305	2.588162
H	0.685846	0.772328	1.427155
H	1.537664	2.626491	-2.369800
H	2.044419	4.772705	-1.213393
C	2.167951	-1.889789	-0.161680
C	1.949925	-3.128965	0.459980
C	3.480134	-1.435012	-0.355506
C	4.559462	-2.208073	0.066862
C	4.336874	-3.441125	0.682383
C	3.031191	-3.899668	0.877473
H	0.924134	-3.485175	0.612064
H	2.855794	-4.868237	1.360656
H	3.639656	-0.462294	-0.838549
H	5.583303	-1.846190	-0.085278
H	5.186464	-4.050347	1.013628
C	-1.614914	-0.886781	-0.405373
C	-2.491405	0.167280	-0.700444
C	-3.853136	0.041779	-0.434368
C	-4.350102	-1.137761	0.123027
C	-3.481537	-2.192474	0.416646
C	-2.119597	-2.069621	0.156639
H	-1.435624	-2.896039	0.383876
H	-5.422342	-1.237327	0.330472
H	-3.871529	-3.119791	0.852934
H	-2.085876	1.089182	-1.137071
H	-4.533313	0.870591	-0.664124

1,2-Diphenylcyclopropene (**2b**), PCM = THF

E_0 = -578.192681
E (298 K) = -578.180589
H (298 K) = -578.179644
G (298 K) = -578.233052
Imaginary frequencies = 0

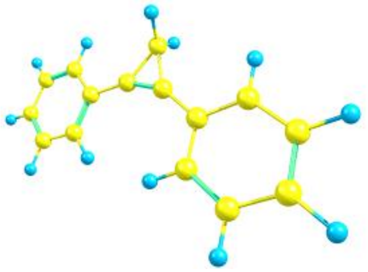

Cartesian coordinates:

C	-3.049442	0.584125	-0.016217
C	-2.218220	-0.673337	-0.027454
C	-1.751623	0.759887	-0.016210
H	-2.138231	-1.263367	-0.958205
H	-2.136174	-1.278501	0.893345
C	-0.552859	1.579633	-0.010909
C	-0.622934	2.981855	0.002162
C	0.543404	3.741228	0.007071
C	1.791174	3.111718	-0.001111

3-Ethyl-1,2-diphenylcyclopropene (**2c**), PCM = THF

E_0 = -656.712452
E (298 K) = -656.697408
H (298 K) = -656.696464
G (298 K) = -656.756802
Imaginary frequencies = 0

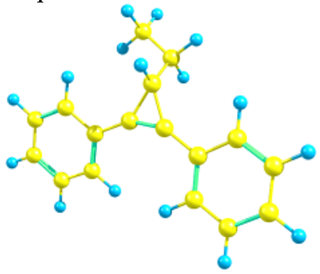

Cartesian coordinates:

C	-6.051096	0.414588	0.276192
C	-5.585322	-0.810270	0.334128
C	-4.581572	0.282470	0.594117
C	-7.130791	1.372091	0.095229
C	-6.933308	2.713135	0.456152
C	-7.961764	3.641364	0.307524
C	-9.194951	3.242022	-0.210742
C	-9.397484	1.909260	-0.578410
C	-8.373714	0.978327	-0.425396

C	1.868474	1.718059	-0.014183
C	0.702671	0.954997	-0.019022
H	0.749549	-0.141263	-0.029191
H	-1.602476	3.474202	0.008586
H	0.480751	4.835950	0.017358
H	2.708404	3.712837	0.002769
H	2.846342	1.221793	-0.020634
C	-4.423640	1.053958	-0.011203
C	-4.732158	2.423696	0.000205
C	-6.059464	2.842425	0.004430
C	-7.092822	1.901513	-0.002636
C	-6.793539	0.538162	-0.013900
C	-5.465855	0.115738	-0.018168
H	-5.217133	-0.952966	-0.027098
H	-3.920722	3.160923	0.005738
H	-6.292603	3.913887	0.013350
H	-8.137636	2.234684	0.000705
H	-7.602552	-0.202119	-0.019457

H	-8.528335	-0.066633	-0.719802
H	-5.959732	3.016275	0.862299
H	-7.799543	4.686700	0.596568
H	-10.003157	3.973439	-0.331187
H	-10.363924	1.593549	-0.989377
C	-5.726760	-2.254467	0.261715
C	-6.974239	-2.861827	0.046847
C	-7.076906	-4.248658	-0.014413
C	-5.938046	-5.044528	0.136368
C	-4.694600	-4.447841	0.352024
C	-4.588878	-3.060001	0.415665
H	-3.617039	-2.578828	0.585131
H	-7.867828	-2.236191	-0.064949
H	-8.055014	-4.715790	-0.180680
H	-6.021958	-6.136837	0.086420
H	-3.799628	-5.070078	0.472037
C	-3.513229	0.644898	-0.431028
H	-4.263911	0.455734	1.642268
C	-3.237610	2.148737	-0.477833
H	-3.841117	0.288983	-1.426952
H	-2.577701	0.101467	-0.191119
H	-4.132525	2.698630	-0.823504
H	-2.404716	2.392676	-1.161113
H	-2.974237	2.531405	0.526537

1,2-Diphenyl-3-vinylcyclopropene (**2d**), PCM = THF

E_0 = -655.511005
E (298 K) = -655.496515
H (298 K) = -655.495570
G (298 K) = -655.554702
Imaginary frequencies = 0

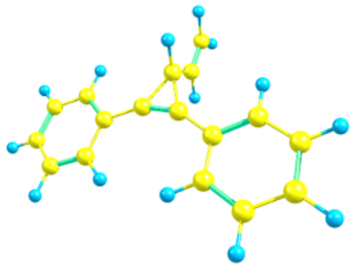

Cartesian coordinates:

C	-5.230075	0.965871	0.262665
C	-4.330310	0.018179	0.311516
C	-3.810485	1.424615	0.516129
C	-6.575124	1.483608	0.084331
C	-6.780157	2.870151	0.127143
C	-8.059176	3.395910	-0.042609
C	-9.142730	2.541904	-0.255409
C	-8.944560	1.159149	-0.299946
C	-7.668192	0.629987	-0.131971
H	-5.918413	3.529452	0.292924
H	-8.212710	4.481122	-0.008684
H	-10.149683	2.955431	-0.388512
H	-9.795094	0.487752	-0.467935

1,2-Diphenyl-3-(phenylethynyl)cyclopropene (**2e**), PCM = THF

E_0 = -885.134900
E (298 K) = -885.116102
H (298 K) = -885.115158
G (298 K) = -885.186969
Imaginary frequencies = 0

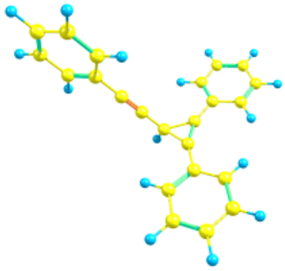

Cartesian coordinates:

C	-4.657849	1.240480	-1.022050
C	-4.062208	0.080008	-1.088988
C	-3.386340	1.205491	-1.842452
C	-5.681389	2.166482	-0.573173
C	-5.560658	3.525077	-0.896799
C	-6.527828	4.433474	-0.472422
C	-7.621752	3.991524	0.273671
C	-7.747024	2.637865	0.597596
C	-6.781747	1.726971	0.178704
H	-6.876451	0.664049	0.430439
H	-4.694350	3.859007	-1.481986
H	-6.428002	5.495513	-0.725971
H	-8.383617	4.706858	0.606062

H	-7.509416	-0.454348	-0.167408
C	-3.875539	-1.358820	0.233774
C	-4.776685	-2.422385	0.069789
C	-4.306629	-3.730519	-0.002949
C	-2.936234	-3.990127	0.085383
C	-2.035246	-2.936375	0.248244
C	-2.501798	-1.625783	0.323605
H	-1.805066	-0.787091	0.449952
H	-5.851470	-2.215902	0.002791
H	-5.015129	-4.557756	-0.129787
H	-2.569224	-5.021909	0.027005
H	-0.959680	-3.138277	0.317519
C	-3.036263	2.103951	-0.561210
H	-3.531368	1.741162	1.538943
C	-2.082841	3.014409	-0.345494
H	-3.294636	1.809902	-1.591827
H	-1.812651	3.319122	0.675848
H	-1.540500	3.486354	-1.173824

H	-8.606393	2.290304	1.183138
C	-3.887218	-1.326143	-0.774752
C	-4.840106	-2.028857	-0.021327
C	-4.644215	-3.376510	0.265759
C	-3.498407	-4.032282	-0.192580
C	-2.546851	-3.336694	-0.940406
H	-3.346896	-5.094069	0.036003
C	-2.739566	-1.988256	-1.232461
H	-1.646661	-3.849819	-1.298940
H	-5.737743	-1.510956	0.336744
H	-5.391878	-3.922570	0.853138
H	-1.998328	-1.428335	-1.816840
C	-2.160825	1.805562	-1.322694
H	-3.459217	1.231528	-2.943364
C	-1.138282	2.309461	-0.896584
C	0.071831	2.903301	-0.383242
C	0.288984	2.984561	1.001556
C	1.459204	3.557601	1.494000
C	2.423203	4.054841	0.615071
C	2.212085	3.976899	-0.762774
C	1.044425	3.405115	-1.262716
H	2.966076	4.366060	-1.457433
H	0.874291	3.341293	-2.343814
H	-0.471206	2.592507	1.687186
H	1.620021	3.616491	2.577129
H	3.343498	4.505204	1.005785

N,*N*-Dimethyl-2,3-diphenylcycloprop-2-ene-1-carbonitrile (**2f**), PCM = THF

E_0 = -670.407466
E (298 K) = -670.393655
H (298 K) = -670.392710
G (298 K) = -670.450322
Imaginary frequencies = 0

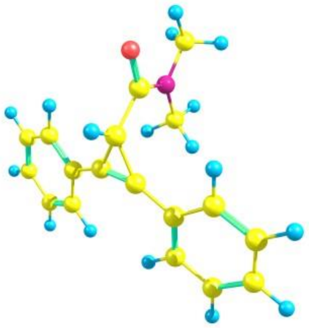

Cartesian coordinates:

H	-8.685694	2.069685	1.091844
H	-7.736369	4.374453	0.990138
C	-7.664960	2.227145	0.723754
H	-7.314016	0.122905	0.359973
C	-7.132486	3.518168	0.666484
H	-7.093261	-4.684720	0.553063
C	-6.901177	1.137661	0.315364
H	-6.761688	-2.244450	0.177155
C	-6.125979	-4.313023	0.194871

2,3-Diphenylcycloprop-2-ene-1-carbonitrile (**2g**), PCM = THF

E_0 = -670.407466
E (298 K) = -670.393655
H (298 K) = -670.392710
G (298 K) = -670.450322
Imaginary frequencies = 0

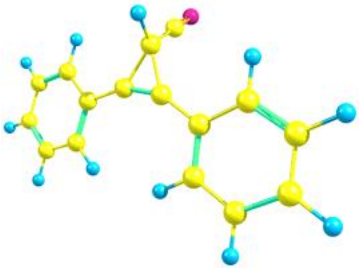

Cartesian coordinates:

C	-6.118600	0.489565	0.344580
C	-5.607997	-0.710920	0.387263
C	-4.661574	0.413641	0.736864
C	-7.170389	1.467589	0.138385
C	-6.876041	2.829968	0.283170
C	-7.872846	3.782982	0.087167
C	-9.165605	3.381266	-0.252671
C	-9.462527	2.023464	-0.398203
C	-8.470614	1.067101	-0.204744
H	-8.698685	0.000852	-0.318200
H	-5.855546	3.133373	0.549129

C	-5.833351	3.718618	0.196458
C	-5.943379	-2.948936	-0.013834
C	-5.595484	1.334224	-0.160584
H	-5.415157	4.731181	0.150538
H	-5.227383	-6.280680	0.117311
C	-5.066733	2.631662	-0.218761
C	-5.079746	-5.206945	-0.050107
C	-4.765798	0.216442	-0.569542
C	-4.707432	-2.469199	-0.475317
C	-4.472965	-1.051719	-0.676467
H	-4.045182	2.775452	-0.594147
H	-3.395802	-1.213430	1.670946
C	-3.848387	-4.734597	-0.507712
C	-3.661871	-3.370429	-0.722309
C	-3.415526	-0.093238	-1.170111
H	-3.276461	0.030329	-2.258352
H	-3.766690	0.543598	1.768421
H	-3.027271	-5.435212	-0.700602
C	-2.957616	-0.207749	1.802394
H	-2.503007	-0.166123	2.805527
H	-2.700080	-2.985077	-1.084863
C	-2.065107	0.145347	-0.516906
N	-1.903299	0.070353	0.833233
O	-1.121115	0.401773	-1.269659
C	-0.574605	0.289000	1.386174
H	-0.239924	-0.603016	1.949059
H	-0.580129	1.155164	2.075019
H	0.120015	0.483531	0.558243

H	-7.639232	4.848131	0.199923
H	-9.949870	4.132113	-0.406460
H	-10.477958	1.708477	-0.665729
C	-5.629185	-2.156764	0.267086
C	-6.810279	-2.835504	-0.069453
C	-6.805863	-4.222595	-0.178956
C	-5.627529	-4.940061	0.044004
C	-4.450613	-4.267755	0.377176
C	-4.449282	-2.879312	0.489382
H	-3.530071	-2.339324	0.749519
H	-7.732259	-2.268652	-0.244157
H	-7.729818	-4.751032	-0.441543
H	-5.627797	-6.033105	-0.043931
H	-3.525682	-4.830166	0.550523
C	-3.637332	0.815737	-0.227750
H	-4.350822	0.582854	1.780951
N	-2.825650	1.134652	-0.989870

Methyl 2,3-diphenylcycloprop-2-ene-1-carboxylate (**2h**), PCM = THF

E_0 = -805.955099
E (298 K) = -805.938469
H (298 K) = -805.937525
G (298 K) = -805.002974
Imaginary frequencies = 0

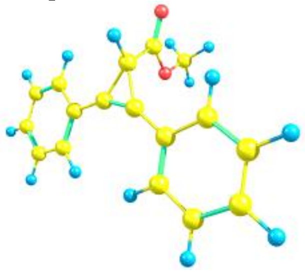

Cartesian coordinates:

C	-4.679169	0.167128	-0.585694
C	-4.389036	-1.096858	-0.717446
C	-3.390007	-0.133192	-1.314288
C	-5.479320	1.293716	-0.142077
C	-5.023284	2.596076	-0.387386
C	-5.773900	3.691277	0.034517
C	-6.983322	3.493144	0.702525
C	-7.442155	2.196373	0.949379
C	-6.695513	1.099314	0.530368

2,3-Diphenylcycloprop-2-ene-1-carboxylic acid (**2i**), PCM = THF

E_0 = -766.711100
E (298 K) = -766.696128
H (298 K) = -766.695184
G (298 K) = -766.756545
Imaginary frequencies = 0

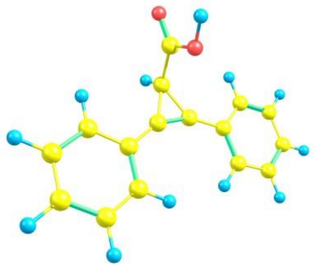

Cartesian coordinates:

C	-4.527138	0.193120	-0.485044
C	-4.238314	-1.070814	-0.616793
C	-3.232016	-0.108101	-1.204772
C	-5.331272	1.319665	-0.049115
C	-4.901513	2.622314	-0.336770
C	-5.658321	3.715703	0.079414
C	-6.845562	3.515495	0.785734
C	-7.277114	2.218227	1.076007
C	-6.525590	1.122946	0.660720

H	-7.051914	0.080535	0.722725
H	-4.071100	2.738575	-0.914216
H	-5.412536	4.708112	-0.159418
H	-7.574209	4.355320	1.034289
H	-8.392147	2.040483	1.474126
C	-4.602899	-2.521320	-0.540124
C	-5.758954	-3.008986	0.088272
C	-5.937376	-4.379566	0.250547
C	-4.966747	-5.273064	-0.210469
C	-3.815266	-4.792503	-0.836064
C	-3.632727	-3.421342	-1.001661
H	-2.732236	-3.031121	-1.492954
H	-5.110406	-6.352472	-0.080693
H	-3.052985	-5.492453	-1.198190
H	-6.518489	-2.305053	0.448232
H	-6.842280	-4.756629	0.741577
C	-2.076045	0.096990	-0.632557
H	-3.313684	-0.002122	-2.406370
O	-1.047241	0.391151	-1.204141
O	-2.150422	-0.057397	0.698624
C	-0.921400	0.152894	1.400377
H	-0.551677	1.178115	1.225543
H	-0.156935	-0.565503	1.056882
H	-1.145633	-0.001217	2.465767

H	-6.859332	0.103698	0.888458
H	-3.965221	2.766954	-0.890856
H	-5.318369	4.733001	-0.148204
H	-7.439838	4.376744	1.113990
H	-8.209150	2.060216	1.631459
C	-4.460315	-2.495398	-0.452377
C	-5.644420	-2.983142	0.121380
C	-5.831284	-4.354070	0.271556
C	-4.840538	-5.247361	-0.145449
C	-3.661231	-4.766505	-0.716781
C	-3.470697	-3.395087	-0.871466
H	-2.548640	-3.004810	-1.320918
H	-4.991321	-6.326972	-0.024818
H	-2.883490	-5.466172	-1.045169
H	-6.420183	-2.279481	0.445816
H	-6.758707	-4.731619	0.718218
C	-1.926220	0.118037	-0.510912
H	-3.147067	0.025411	-2.295903
O	-0.894624	0.425830	-1.068075
O	-1.999643	-0.057741	0.820941
H	-1.110979	0.111395	1.179519

3-Methyl-3-phenylcyclopropene (**2j**), PCM = THF

E_0 = -386.599146

E (298 K) = -386.590419

H (298 K) = -386.589475

G (298 K) = -386.633071

Imaginary frequencies = 0

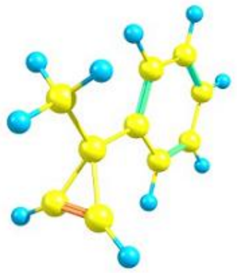

Cartesian coordinates:

C	2.279815	-0.986310	-0.648922
C	2.279855	-0.986252	0.648790
C	1.505145	0.143155	-0.000093
H	2.568846	-1.440046	-1.598773
H	2.568942	-1.439902	1.598664
C	-0.006289	0.063046	-0.000043
C	2.093021	1.546887	-0.000173
H	3.194245	1.490365	-0.000202
H	1.778145	2.118855	0.893058
H	1.778093	2.118777	-0.893436
C	-0.656477	-1.181126	0.000041
C	-2.046825	-1.268890	0.000088

Methyl 1-methylcycloprop-2-ene-1-carboxylate (**2k**), PCM = THF

E_0 = -383.518236

E (298 K) = -383.509567

H (298 K) = -383.508623

G (298 K) = -383.551458

Imaginary frequencies = 0

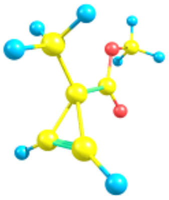

Cartesian coordinates:

C	-1.173006	0.390692	-0.690528
C	-1.094012	0.186116	0.584038
C	-0.164354	1.252201	0.041327
H	-1.599159	0.177523	-1.671144
H	-1.402645	-0.330474	1.493170
C	-0.401419	2.732000	0.293582
H	-1.483031	2.923976	0.391917
H	-0.007386	3.345202	-0.536323
H	0.102196	3.063505	1.219053
C	1.272172	0.823862	-0.116565
O	1.639808	-0.313490	-0.322358
O	2.128835	1.851377	-0.004079
C	3.511510	1.507918	-0.144987
H	3.694988	1.064055	-1.138905

C	-2.826584	-0.110202	0.000053
C	-2.195612	1.132326	-0.000031
C	-0.801284	1.217501	-0.000078
H	-0.331695	2.207681	-0.000143
H	-0.057072	-2.100136	0.000069
H	-2.528091	-2.254962	0.000154
H	-3.921188	-0.177484	0.000090
H	-2.793320	2.052419	-0.000060

H	3.804704	0.779544	0.630948
H	4.074256	2.445472	-0.029146

3-Methyl-1,2,3-triphenylcyclopropene (**2l**), PCM = THF

E_0 = -848.300173
E (298 K) = -848.281832
H (298 K) = -848.280887
G (298 K) = -848.350046
Imaginary frequencies = 0

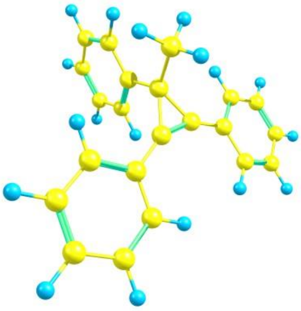

Cartesian coordinates:

H	-5.415209	-1.188715	0.393726
H	-4.516817	0.899961	-0.634247
C	-4.343521	-1.099425	0.178473
H	-3.874928	-3.075685	0.930329
C	-3.841324	0.069152	-0.397546
C	-3.480717	-2.156743	0.480197
C	-2.480169	0.180979	-0.673169
C	-2.118971	-2.047101	0.211028
H	-2.070421	1.094266	-1.124547
C	-1.608775	-0.875285	-0.368796
H	-1.439567	-2.875780	0.444446
C	-0.194167	-0.717893	-0.656550
H	0.217003	1.090827	-3.224219
H	0.630201	-0.650525	-3.240744
H	0.555194	0.711464	1.399497
C	0.779233	0.255883	-1.283232
C	0.895047	0.325754	-2.800500
H	0.947376	-3.407657	0.775151
C	0.900747	1.602309	0.858388
C	1.068655	-1.050482	-0.576722
C	1.070440	1.538708	-0.534298
H	1.018418	2.798624	2.649900
C	1.161275	2.775724	1.562227
C	1.510313	2.692581	-1.196702
H	1.651383	2.681421	-2.283624
C	1.601642	3.918656	0.890019
C	1.774733	3.869840	-0.492028

1-Chloro-2-phenylcyclopropene (**2m**), PCM = THF

E_0 = -806.937394
E (298 K) = -806.928797
H (298 K) = -806.927853
G (298 K) = -806.972529
Imaginary frequencies = 0

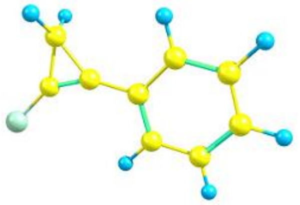

Cartesian coordinates:

H	-1.808248	2.370182	0.927564
C	-1.682833	1.783966	0.000456
H	1.364074	2.253500	-0.000426
C	-0.728301	0.586829	0.000407
C	1.663018	1.197845	-0.000245
C	-2.001691	0.328639	0.000085
H	3.782208	1.616904	-0.000896
C	0.672871	0.207211	0.000241
C	3.009728	0.838711	-0.000496
C	1.042093	-1.146953	0.000511
C	3.373505	-0.508626	-0.000236
Cl	-3.254077	-0.827587	-0.000398
H	0.262039	-1.918500	0.000912
C	2.388188	-1.500109	0.000277
H	4.433419	-0.790110	-0.000425
H	2.674276	-2.558626	0.000496
H	-1.807918	2.370553	-0.926465

H	1.807503	4.842949	1.443152
H	1.924220	0.577241	-3.120313
C	1.970842	-3.074405	0.565012
H	2.119279	4.759159	-1.034649
C	2.178552	-1.873876	-0.131481
H	2.890683	-4.771812	1.529876
C	3.058568	-3.834334	0.986384
C	3.487762	-1.447316	-0.398118
H	3.639700	-0.504206	-0.939562
C	4.360975	-3.404352	0.717726
C	4.573736	-2.210254	0.026096
H	5.216064	-4.004942	1.050761
H	5.595174	-1.870951	-0.183988

1-Methyl-2-phenylcyclopropene (**2n**), PCM = THF

E_0 = -386.610784
E (298 K) = -386.601487
H (298 K) = -386.600543
G (298 K) = -386.646113
Imaginary frequencies = 0

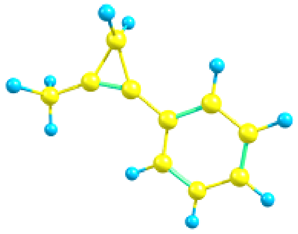

Cartesian coordinates:

C	1.153433	0.380550	0.000720
C	2.203031	1.462093	0.001667
C	2.388802	-0.038241	-0.000033
H	2.398660	2.035238	0.926839
H	2.398363	2.037222	-0.922345
C	-0.281178	0.135746	0.000402
C	-1.173614	1.215828	-0.000806
C	-2.550125	0.991894	-0.001285
C	-3.046551	-0.312568	-0.000467
C	-2.161538	-1.394755	0.000851
C	-0.786832	-1.174189	0.001273
H	-0.087738	-2.020078	0.002395
H	-0.771895	2.237103	-0.001405
H	-3.241808	1.843073	-0.002302
H	-4.128957	-0.489155	-0.000815
H	-2.549436	-2.420644	0.001575
C	3.408486	-1.111871	-0.001856
H	4.059983	-1.016035	-0.889282
H	2.943038	-2.111111	-0.006404
H	4.056299	-1.022542	0.888951

1-Phenyl-2-(trimethylsilyl)cyclopropene (**2o**), PCM = THF

E_0 = -755.826082
E (298 K) = -755.810628
H (298 K) = -755.809684
G (298 K) = -755.870455
Imaginary frequencies = 0

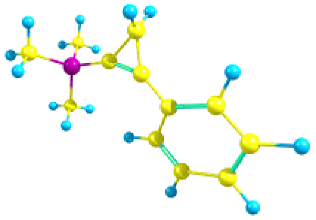

Cartesian coordinates:

C	0.353239	-0.938213	-0.000387
C	-0.947949	-0.773090	-0.000250
C	-0.412712	-2.215254	-0.000125
H	-0.481763	-2.814544	0.924863
H	-0.481986	-2.814782	-0.924940
C	1.687613	-0.356220	-0.000239
C	2.817069	-1.184850	0.000009
C	4.095671	-0.627699	0.000228
C	4.253667	0.759001	0.000193
C	3.129871	1.591049	-0.000070
C	1.852841	1.037325	-0.000284
Si	-2.507052	0.262756	0.000050
C	-2.033694	2.084149	-0.000860
H	-1.438519	2.339295	0.895066
H	-2.937191	2.721242	-0.001167
H	-1.438575	2.338375	-0.897090
C	-3.488990	-0.176182	-1.545022
H	-2.918834	0.066362	-2.459798
H	-4.440507	0.386048	-1.574078
H	-3.728120	-1.255011	-1.564786
C	-3.487537	-0.175019	1.546375
H	-2.917063	0.069628	2.460395
H	-3.725371	-1.254098	1.567969
H	-4.439696	0.386137	1.575198
H	2.678289	-2.273499	0.000036

H	4.976726	-1.280729	0.000433
H	5.259637	1.196188	0.000372
H	3.253827	2.680727	-0.000100
H	0.963327	1.680094	-0.000476

Parent cyclopropene (**2p**), PCM = THF

E_0 = -116.493751
E (298 K) = -116.490422
H (298 K) = -116.489478
G (298 K) = -116.517054
Imaginary frequencies = 0

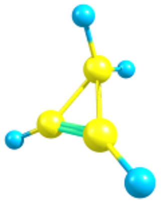

Cartesian coordinates:

C	0.501210	-0.648999	0.000000
C	0.501210	0.648999	0.000000
C	-0.862973	0.000000	0.000000
H	-1.467727	0.000000	0.923494
H	-1.467727	0.000000	-0.923494
H	1.049383	-1.591586	0.000000
H	1.049383	1.591586	0.000000

Cycloadduct **4-*endo***, PCM = THF

E_0 = -1432.958377
E (298 K) = -1432.932771
H (298 K) = -1432.931827
G (298 K) = -1433.014601
Imaginary frequencies = 0

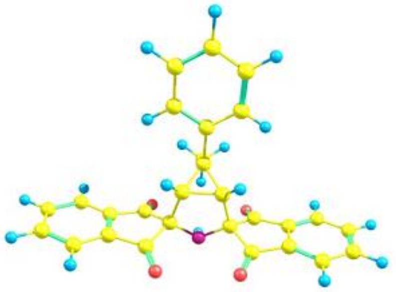

Cartesian coordinates:

C	0.760809	0.338686	-0.394100
C	-0.751947	0.375288	-0.393527
C	0.028343	1.380446	0.425385
H	1.320410	0.677977	-1.274679
H	-1.293541	0.740279	-1.275075
C	0.062877	2.769039	-0.170588
C	0.026129	1.413984	1.944218
H	-0.002545	0.416279	2.397903
H	-0.854368	1.983475	2.293906
H	0.933746	1.935577	2.299233
C	1.284930	3.403616	-0.413550
C	1.320165	4.707639	-0.907924
C	0.130042	5.391400	-1.161593
C	-1.093557	4.765952	-0.917018
C	-1.125269	3.461819	-0.422492
H	-2.087496	2.967385	-0.229636
H	2.220542	2.862942	-0.213750
H	2.284978	5.193391	-1.098188
H	0.156142	6.415538	-1.552989
H	-2.032307	5.297754	-1.114474
C	-5.826014	-1.302954	-0.974333
C	-5.986808	-0.775212	0.318061
C	-4.562865	-1.622442	-1.469529
C	-3.467285	-1.384618	-0.643351
C	-3.627718	-0.858945	0.638307
C	-4.888436	-0.555185	1.147213
H	-6.995406	-0.541910	0.679178
H	-5.001720	-0.158813	2.162954
H	-6.712655	-1.472123	-1.596835
H	-4.424264	-2.048659	-2.470043
C	-2.020709	-1.658564	-0.903336
C	-2.301495	-0.784217	1.310176
O	-2.097301	-0.695747	2.499077

O	-1.563890	-2.270715	-1.834545
C	-1.219522	-0.957109	0.226127
N	-0.041603	-1.650408	0.708734
C	1.167154	-1.011052	0.231826
C	1.953769	-1.756020	-0.879937
C	2.249060	-0.864600	1.319824
C	3.573658	-0.954129	0.645943
C	3.404101	-1.486353	-0.632234
C	4.494893	-1.739498	-1.460010
C	5.762320	-1.425533	-0.971873
C	5.932100	-0.890930	0.316405
C	4.838598	-0.658070	1.148649
H	4.959168	-0.257037	2.161724
H	6.645218	-1.605035	-1.596809
H	6.943775	-0.662708	0.672136
H	4.349921	-2.172260	-2.456801
O	2.044408	-0.781473	2.508871
O	1.489671	-2.407139	-1.780381
H	-0.044345	-1.680232	1.733091

Cycloadduct **4'-*endo***, PCM = THF

E_0 = -1432.949754

E (298 K) = -1432.924184

H (298 K) = -1432.923239

G (298 K) = -1433.005302

Imaginary frequencies = 0

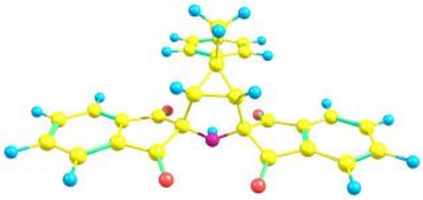

Cartesian coordinates:

C	-0.768108	-0.306228	1.177458
C	0.752557	-0.351294	1.160315
C	0.033083	0.929632	1.523519
H	-1.321330	-0.776842	2.000268
H	1.299477	-0.857649	1.965997
C	0.071150	2.217347	0.747070
C	0.054702	1.214622	3.025030
H	-0.818957	1.829905	3.305587
H	0.969439	1.774254	3.290702
H	0.030495	0.278772	3.611598
C	-1.110931	2.911788	0.473576
C	-1.073609	4.180230	-0.103650
C	0.152864	4.780340	-0.391068
C	1.339341	4.114174	-0.080396
C	1.294835	2.846148	0.495800
H	2.228377	2.331726	0.762775
H	-2.075710	2.452290	0.725846
H	-2.009936	4.706347	-0.325455
H	0.184350	5.777468	-0.846830
H	2.307280	4.587787	-0.283942
C	-5.989985	-0.521026	-0.259413

Cycloadduct **4-*exo***, PCM = THF

E_0 = -1432.956851

E (298 K) = -1432.931116

H (298 K) = -1432.930171

G (298 K) = -1433.013198

Imaginary frequencies = 0

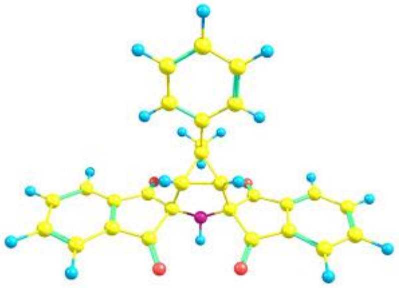

Cartesian coordinates:

C	0.759111	0.296183	-0.328330
C	-0.750436	0.333905	-0.328166
C	0.028514	1.342256	0.489560
H	1.326010	0.629110	-1.206400
H	-1.298188	0.693384	-1.208006
C	0.066353	2.730152	-0.105387
C	0.022617	1.356105	2.008045
H	-0.005573	0.343059	2.430831
H	-0.860913	1.917402	2.363022
H	0.928686	1.873136	2.373074
C	1.290469	3.362041	-0.345479
C	1.329998	4.665761	-0.840195
C	0.141986	5.351986	-1.097387
C	-1.083632	4.729327	-0.855794
C	-1.119575	3.425436	-0.360791
H	-2.083400	2.933195	-0.169992
H	2.224273	2.819095	-0.142691
H	2.296368	5.149414	-1.027941

C	-5.827114	-1.838554	0.200852
C	-4.891474	0.253509	-0.628470
C	-3.628578	-0.319884	-0.500341
C	-3.465749	-1.628375	-0.044473
C	-4.561464	-2.413195	0.305690
H	-6.713978	-2.423400	0.472277
H	-4.421356	-3.445556	0.647041
H	-7.000422	-0.102566	-0.336944
H	-5.007419	1.275979	-1.006555
C	-2.297951	0.229808	-0.878786
C	-2.015893	-1.996610	-0.062296
O	-1.554857	-3.099157	0.088101
O	-2.074840	1.142526	-1.638704
C	-1.221263	-0.675701	-0.253476
N	-0.045177	-0.731981	-1.091554
C	1.153767	-0.721041	-0.286875
C	2.236718	0.171625	-0.920003
C	1.934556	-2.056342	-0.138974
C	3.385183	-1.690322	-0.075198
C	3.560520	-0.378071	-0.517217
C	4.826428	0.195100	-0.609601
C	5.914776	-0.582517	-0.216787
C	5.739576	-1.903405	0.228325
C	4.471442	-2.478939	0.294396
H	4.323091	-3.514386	0.622696
H	6.926881	-0.163497	-0.263572
H	6.618802	-2.490567	0.518955
H	4.952978	1.220249	-0.977076
O	1.472201	-3.165712	-0.065604
O	2.020536	1.069933	-1.698365
H	-0.036733	0.094162	-1.703070

H	0.171363	6.375929	-1.489086
H	-2.020639	5.263220	-1.055913
C	-5.772751	-1.271423	-1.118237
C	-5.987676	-0.677970	0.137338
C	-4.494555	-1.648769	-1.525897
C	-3.439759	-1.400032	-0.650630
C	-3.652526	-0.810505	0.594464
C	-4.929952	-0.448150	1.015490
H	-7.007314	-0.400429	0.430007
H	-5.085939	-0.000799	2.004171
H	-6.628579	-1.445651	-1.781160
H	-4.314244	-2.125698	-2.496436
C	-1.993653	-1.728506	-0.811503
C	-2.361566	-0.728067	1.340059
O	-2.241061	-0.544261	2.526887
O	-1.507207	-2.444380	-1.650691
C	-1.227673	-0.980128	0.311072
N	-0.041373	-1.620051	0.845055
C	1.174457	-1.033908	0.320569
C	1.926963	-1.836290	-0.772231
C	2.312262	-0.801436	1.351189
C	3.598498	-0.893019	0.597306
C	3.374752	-1.500589	-0.637776
C	4.422619	-1.766322	-1.516082
C	5.704407	-1.383297	-1.124659
C	5.930195	-0.771379	0.119862
C	4.879866	-0.527919	1.003400
H	5.044871	-0.067912	1.984798
H	6.554639	-1.568462	-1.791807
H	6.952480	-0.490863	0.400202
H	4.235203	-2.259274	-2.477204
O	2.198086	-0.623042	2.539177
O	1.435759	-2.612731	-1.552677
H	-0.061951	-2.633564	0.723832

Cycloadduct **4'-*exo***, PCM = THF

E_0 = -1432.943661

E (298 K) = -1432.917789

H (298 K) = -1432.916845

G (298 K) = -1432.999548

Imaginary frequencies = 0

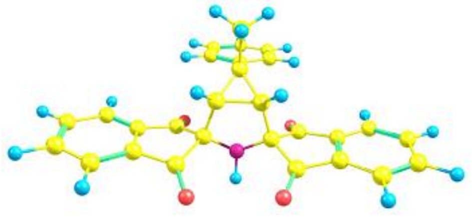

Cartesian coordinates:

C	-0.764237	-0.318861	1.070900
C	0.755319	-0.287909	1.084139
C	-0.031676	0.954908	1.440329
H	-1.316355	-0.817356	1.877606
H	1.309965	-0.764615	1.902265

Cycloadduct **5a-*endo***, PCM = THF

E_0 = -1853.306889

E (298 K) = -1853.281526

H (298 K) = -1853.280582

G (298 K) = -1853.362401

Imaginary frequencies = 0

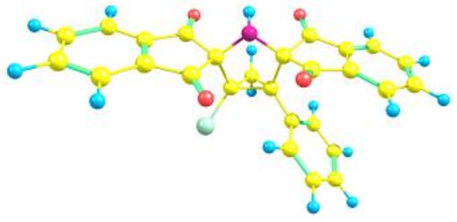

Cartesian coordinates:

C	-5.362732	1.722574	0.247643
C	-5.254909	1.222900	-1.060722
C	-4.227750	2.000996	1.007856
C	-2.987746	1.747052	0.426704
C	-2.880760	1.256356	-0.876339

C	-0.044384	2.254511	0.684997	C	-4.009597	0.993337	-1.645322
C	-0.049623	1.217683	2.946092	H	-6.168546	1.014848	-1.630193
H	-0.956998	1.785298	3.219833	H	-3.911728	0.608638	-2.667419
H	0.832013	1.817206	3.235489	H	-6.357823	1.896182	0.673890
H	-0.037854	0.273202	3.519387	H	-4.299219	2.392397	2.029310
C	-1.253162	2.912584	0.438569	C	-1.626483	1.894048	1.005215
C	-1.267216	4.191487	-0.115365	C	-1.439216	1.081490	-1.235974
C	-0.065969	4.839478	-0.406133	O	-0.978950	0.974716	-2.343752
C	1.145975	4.210516	-0.118765	O	-1.286108	2.495707	1.998505
C	1.153090	2.931296	0.434874	C	-0.667166	1.102059	0.101450
H	2.106479	2.449867	0.688094	N	0.677074	1.634547	0.099694
H	-2.198629	2.415805	0.693016	C	1.652647	0.564520	0.227658
H	-2.224169	4.687843	-0.316964	C	2.838554	1.003497	1.103451
H	-0.074511	5.844679	-0.844979	C	2.324474	0.124635	-1.094913
H	2.094588	4.721712	-0.322661	C	3.799952	0.157471	-0.877745
C	-5.936594	-0.668027	-0.010068	C	4.093772	0.634249	0.401184
C	-5.704831	-1.994535	0.390598	C	5.408013	0.748310	0.850198
C	-4.892293	0.144163	-0.449840	C	6.422336	0.361300	-0.022800
C	-3.609751	-0.398859	-0.451637	C	6.127184	-0.119662	-1.309991
C	-3.380010	-1.716579	-0.054777	C	4.810993	-0.227097	-1.754945
C	-4.422702	-2.540817	0.361706	H	4.566766	-0.599160	-2.756704
H	-6.550509	-2.609119	0.721316	H	7.469310	0.433752	0.294289
H	-4.231122	-3.580040	0.653795	H	6.950148	-0.412837	-1.972493
H	-6.959008	-0.272547	0.015048	H	5.623573	1.129698	1.854990
H	-5.063957	1.173973	-0.784904	O	1.745414	-0.231754	-2.090743
C	-2.325950	0.203392	-0.920518	O	2.718134	1.584508	2.157544
C	-1.932236	-2.042637	-0.225455	H	0.777034	2.273059	0.897590
O	-1.442075	-3.142370	-0.275967	H	0.242065	0.605369	2.566299
O	-2.205086	1.145756	-1.662238	C	0.077251	-0.352103	2.054811
C	-1.185278	-0.685453	-0.364608	H	-2.804068	-1.152870	2.044514
N	0.020040	-0.643698	-1.148266	C	-0.584298	-0.339282	0.692845
C	1.212294	-0.652993	-0.341256	C	-2.710513	-1.628434	1.058442
C	2.337964	0.247452	-0.906830	C	0.890770	-0.625067	0.827804
C	1.980525	-1.993542	-0.148220	H	-4.587492	-2.689691	1.209917
C	3.427577	-1.646390	-0.026061	C	-1.592583	-1.353990	0.265767
C	3.635388	-0.334693	-0.451931	C	-3.707424	-2.483353	0.589150
C	4.911667	0.222319	-0.482503	C	-1.466224	-1.952412	-0.993630
C	5.972499	-0.570297	-0.046851	C	-3.584399	-3.071654	-0.670925
C	5.762530	-1.891216	0.383853	Cl	1.563197	-2.237728	0.573200
C	4.486018	-2.450694	0.390140	H	-0.584271	-1.718658	-1.607540
H	4.309562	-3.484965	0.708223	C	-2.460789	-2.811376	-1.458740
H	6.990796	-0.163545	-0.047469	H	-4.369406	-3.742191	-1.041152
H	6.620686	-2.490098	0.711205	H	-2.360552	-3.278561	-2.445710
H	5.065675	1.247866	-0.838409	H	-0.138336	-1.216083	2.692548
O	1.499665	-3.097412	-0.095026				
O	2.196629	1.184894	-1.651681				
H	0.034910	-1.297525	-1.929650				

Cycloadduct **5a-*exo***, PCM = THF	**TS-4-*endo***, PCM = THF
E_0 = -1853.302950	E_0 = -1432.854105
E (298 K) = -1853.277418	E (298 K) = -1432.827609
H (298 K) = -1853.276474	H (298 K) = -1432.826665
G (298 K) = -1853.358476	G (298 K) = -1432.912343

Imaginary frequencies = 0

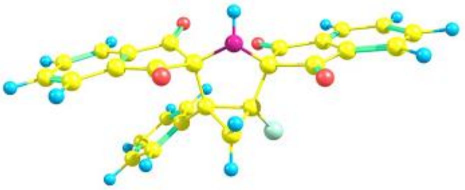

Cartesian coordinates:

C	-6.094405	-0.340792	1.309732
C	-6.431352	0.385572	0.154355
C	-4.763718	-0.550462	1.664603
C	-3.781957	-0.011895	0.835365
C	-4.116109	0.708009	-0.312632
C	-5.445576	0.919387	-0.672766
H	-7.488801	0.531877	-0.095673
H	-5.693534	1.487376	-1.576973
H	-6.895541	-0.747062	1.938432
H	-4.485193	-1.116720	2.560975
C	-2.303073	-0.110327	0.976235
C	-2.884046	1.170538	-1.008704
O	-2.820524	1.861748	-1.996550
O	-1.694170	-0.674316	1.852699
C	-1.664725	0.601940	-0.244561
N	-0.669047	1.623494	0.034801
C	0.679022	1.110970	-0.026000
C	1.411546	0.836832	1.305404
C	1.698070	2.023142	-0.757659
C	3.031000	1.737969	-0.155324
C	2.866729	1.022653	1.032129
C	3.961182	0.590811	1.775387
C	5.231597	0.885408	1.281634
C	5.397868	1.611871	0.090755
C	4.297197	2.055190	-0.640769
H	4.412473	2.622877	-1.571592
H	6.118977	0.549712	1.831326
H	6.411502	1.831521	-0.264913
H	3.819015	0.028213	2.705710
O	1.448197	2.768786	-1.673791
O	0.893912	0.604002	2.370017
H	-0.858000	2.160739	0.880653
H	-0.241575	1.142050	-2.515256
C	-0.083408	0.096680	-2.223729
H	2.800281	-0.738587	-2.362709
C	0.576339	-0.175306	-0.886775
C	2.696265	-1.391112	-1.484909
C	-0.898241	-0.420146	-1.078077
H	4.566302	-2.422222	-1.821965
C	1.576075	-1.261089	-0.660636
C	3.683176	-2.328425	-1.178700
C	1.435868	-2.084159	0.463582
C	3.546621	-3.142095	-0.052700
Cl	-1.578406	-2.046606	-1.168790
H	0.551417	-1.960725	1.105668

Imaginary frequencies = 1 (-334 cm^{-1})

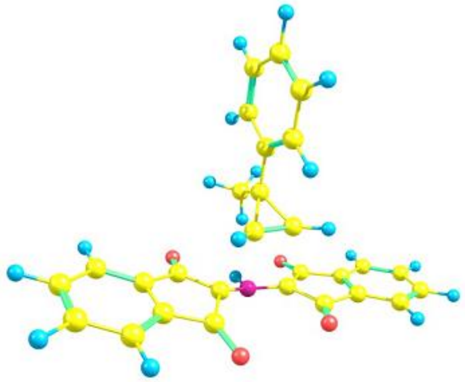

Cartesian coordinates:

C	0.698444	0.566230	-0.515273
C	-0.600839	0.885874	-0.487107
C	0.289466	1.666593	0.451199
H	1.512161	0.407023	-1.229809
H	-1.445810	1.076894	-1.154528
C	0.654427	3.074116	0.027570
C	0.219805	1.449695	1.954653
H	-0.204286	0.469640	2.211240
H	-0.436201	2.212589	2.413053
H	1.215713	1.522763	2.428911
C	1.055457	4.040882	0.960670
C	1.390546	5.333961	0.555750
C	1.327241	5.690735	-0.790811
C	0.931864	4.737839	-1.730653
C	0.603152	3.444914	-1.325137
H	0.297212	2.707760	-2.078451
H	1.112884	3.788057	2.025189
H	1.703242	6.071142	1.305879
H	1.587038	6.707964	-1.107684
H	0.878109	5.002961	-2.793905
C	5.596560	-1.648394	0.079948
C	5.284117	-2.058377	-1.223770
C	4.591621	-1.412131	1.021235
C	3.274806	-1.588093	0.612366
C	2.963240	-1.990906	-0.688807
C	3.957834	-2.241841	-1.624517
H	6.096466	-2.241227	-1.937205
H	3.699414	-2.571615	-2.637714
H	6.647389	-1.517814	0.364195
H	4.824172	-1.103097	2.046973
C	2.009758	-1.442688	1.386954
C	1.474892	-2.146881	-0.858166
O	0.924839	-2.695639	-1.782732
O	1.856848	-1.248696	2.576207
C	0.899483	-1.585304	0.400362
N	-0.330523	-1.612482	0.918331
C	-1.508372	-1.271657	0.404434
C	-2.583254	-1.000955	1.400050
C	-2.114020	-1.331921	-0.956619
C	-3.563909	-0.985541	-0.733005

C	2.419898	-3.024195	0.764422
H	4.323941	-3.877201	0.188862
H	2.309350	-3.667536	1.645537
H	0.125213	-0.621020	-3.024793

C	-3.834086	-0.784216	0.622912
C	-5.105323	-0.441734	1.067745
C	-6.110437	-0.308427	0.106510
C	-5.840274	-0.511042	-1.254102
C	-4.557439	-0.851919	-1.693011
H	-4.330607	-1.012502	-2.753562
H	-7.127195	-0.041947	0.418098
H	-6.651211	-0.399294	-1.983592
H	-5.303678	-0.285771	2.134477
O	-1.605979	-1.553205	-2.031051
O	-2.408902	-0.965203	2.602401
H	-0.324153	-1.537085	1.953241

TS-4'-*endo*, PCM = THF

E_0 = -1432.854596

E (298 K) = -1432.828209

H (298 K) = -1432.827265

G (298 K) = -1432.910770

Imaginary frequencies = 1 (-355 cm^{-1})

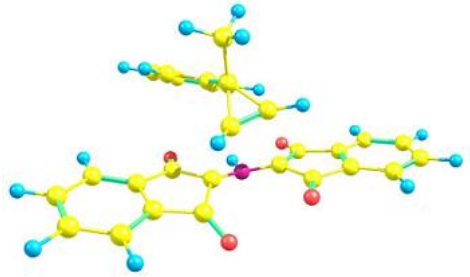

Cartesian coordinates:

C	-0.496720	-0.510664	1.553414
C	0.740526	0.008149	1.681609
C	-0.403258	0.928347	2.015979
H	-1.039294	-1.410327	1.859656
H	1.701143	-0.258273	2.129889
C	-0.807484	2.047031	1.087126
C	-0.667524	1.242131	3.486667
H	-1.741580	1.432373	3.666199
H	-0.112079	2.147852	3.792464
H	-0.352788	0.401559	4.128929
C	-2.139732	2.480872	1.049795
C	-2.522603	3.519885	0.204911
C	-1.579449	4.135281	-0.621938
C	-0.250555	3.715586	-0.585294
C	0.131946	2.687243	0.278864
H	1.180938	2.371260	0.335159
H	-2.889263	1.983902	1.681889
H	-3.569662	3.846845	0.180423
H	-1.883666	4.945243	-1.296069
H	0.500010	4.191670	-1.228662
C	-5.506667	-1.364826	-0.236061
C	-5.071445	-2.646456	0.129890
C	-4.600370	-0.392457	-0.665525
C	-3.254973	-0.738802	-0.695412
C	-2.819494	-2.013566	-0.324402
C	-3.718092	-2.991653	0.080636

TS-4-*exo*, PCM = THF

E_0 = -1432.848408

E (298 K) = -1432.822051

H (298 K) = -1432.821106

G (298 K) = -1432.905766

Imaginary frequencies = 1 (-349 cm^{-1})

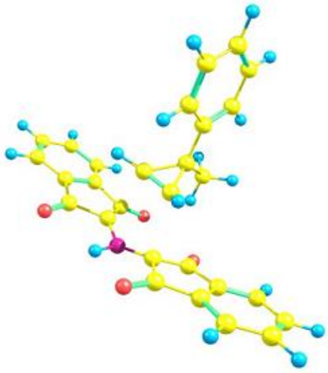

Cartesian coordinates:

C	0.668594	0.696891	-0.588213
C	-0.674930	0.682049	-0.603827
C	-0.025652	1.624629	0.394927
H	1.497142	0.800351	-1.297504
H	-1.488450	0.765692	-1.332950
C	-0.034791	3.090127	0.015893
C	-0.047119	1.338896	1.883636
H	-0.040706	0.259618	2.084223
H	-0.952367	1.772839	2.349184
H	0.835149	1.785740	2.379982
C	-0.054501	4.101483	0.987413
C	-0.061337	5.448027	0.622025
C	-0.049017	5.814483	-0.723683
C	-0.029616	4.819276	-1.701245
C	-0.022636	3.474148	-1.334343
H	-0.007094	2.705998	-2.117726
H	-0.064469	3.840429	2.051249
H	-0.076643	6.219756	1.401590
H	-0.054376	6.873074	-1.010024
H	-0.019730	5.091692	-2.763852
C	-5.868504	-0.977475	-0.302540
C	-5.643432	-0.809846	1.071019
C	-4.819812	-1.291187	-1.170335

H	-5.809027	-3.390085	0.454403
H	-3.367448	-3.994884	0.350265
H	-6.576263	-1.128285	-0.191348
H	-4.928484	0.608644	-0.969733
C	-2.073645	0.053123	-1.137801
C	-1.326129	-2.136189	-0.497936
O	-0.705848	-3.171636	-0.537147
O	-2.035153	1.087739	-1.767889
C	-0.868268	-0.728546	-0.713498
N	0.291843	-0.201618	-1.118125
C	1.519775	-0.361703	-0.629476
C	2.549469	0.600520	-1.118053
C	2.214768	-1.507486	0.026902
C	3.668745	-1.114620	0.031940
C	3.862309	0.105166	-0.619220
C	5.124751	0.673685	-0.736823
C	6.199560	-0.020990	-0.176781
C	6.005648	-1.245421	0.478076
C	4.732015	-1.809356	0.592225
H	4.565065	-2.766600	1.100010
H	7.211644	0.393993	-0.249861
H	6.870121	-1.766977	0.905747
H	5.262480	1.631272	-1.252370
O	1.762666	-2.516733	0.516622
O	2.316067	1.581230	-1.797058
H	0.167137	0.707248	-1.603910

C	-3.550900	-1.424301	-0.619822
C	-3.325269	-1.253308	0.747708
C	-4.364405	-0.950679	1.616629
H	-6.488376	-0.566980	1.726243
H	-4.175472	-0.828870	2.689606
H	-6.884645	-0.863505	-0.697806
H	-4.982848	-1.431680	-2.245195
C	-2.259413	-1.755597	-1.279996
C	-1.876190	-1.478377	1.088272
O	-1.428909	-1.578098	2.207172
O	-2.043381	-2.049627	-2.438578
C	-1.202060	-1.624406	-0.234176
N	0.019634	-1.981335	-0.631263
C	1.234636	-1.608059	-0.230148
C	2.296575	-1.744377	-1.271613
C	1.902133	-1.409785	1.089648
C	3.352119	-1.191924	0.747082
C	3.583951	-1.393962	-0.614839
C	4.854750	-1.269893	-1.163358
C	5.899045	-0.933947	-0.298766
C	5.667630	-0.734395	1.069549
C	4.386548	-0.864723	1.612750
H	4.192201	-0.717211	2.681528
H	6.916666	-0.827227	-0.692224
H	6.509246	-0.474426	1.722481
H	5.022418	-1.434636	-2.234046
O	1.452094	-1.455481	2.210844
O	2.084792	-2.057942	-2.425882
H	0.024387	-2.224414	-1.642371

TS-4'-*exo*, PCM = THF

E_0 = -1432.846428

E (298 K) = -1432.819824

H (298 K) = -1432.818879

G (298 K) = -1432.903497

Imaginary frequencies = 1 (-370 cm^{-1})

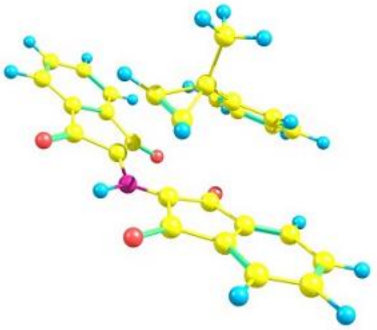

Cartesian coordinates:

C	-0.782949	-0.331563	1.617344
C	0.536760	-0.609659	1.640713
C	0.138380	0.803018	2.000433
H	-1.702873	-0.753004	2.034576
H	1.197035	-1.375874	2.060745
C	0.429191	1.949109	1.065391
C	0.199937	1.221449	3.467755

TS-NI-4, PCM = THF

E_0 = -1432.955065

E (298 K) = -1432.929575

H (298 K) = -1432.928631

G (298 K) = -1433.011232

Imaginary frequencies = 1 (-394 cm^{-1})

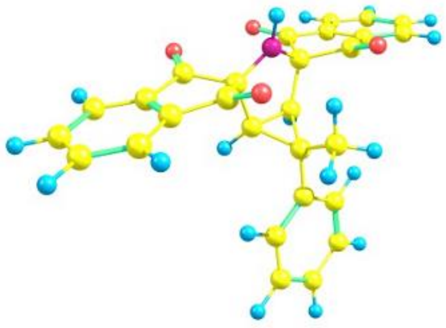

Cartesian coordinates:

C	0.762460	0.312875	-0.374916
C	-0.751914	0.348328	-0.375026
C	0.028084	1.351823	0.446614
H	1.321449	0.651249	-1.255869
H	-1.292884	0.711772	-1.257234
C	0.061703	2.742087	-0.144088
C	0.025313	1.369322	1.965463

H	1.196967	1.631996	3.713602	H	0.000000	0.359564	2.393336
H	-0.001053	0.364286	4.133747	H	-0.857428	1.931990	2.320695
H	-0.545227	2.012255	3.672968	H	0.931372	1.890159	2.325735
C	1.738223	2.435191	0.959391	C	1.283301	3.378874	-0.383868
C	2.030574	3.506171	0.118380	C	1.317843	4.685284	-0.871977
C	1.011113	4.108855	-0.623381	C	0.127326	5.369499	-1.122722
C	-0.297281	3.642401	-0.507429	C	-1.095890	4.741925	-0.881602
C	-0.585651	2.573323	0.342702	C	-1.126754	3.435385	-0.393355
H	-1.615492	2.203534	0.440861	H	-2.088641	2.939293	-0.202884
H	2.539738	1.946923	1.533041	H	2.219166	2.837839	-0.185991
H	3.061627	3.873425	0.035507	H	2.282383	5.172695	-1.059470
H	1.239445	4.947289	-1.292845	H	0.152840	6.395604	-1.509005
H	-1.101994	4.110923	-1.087408	H	-2.034976	5.274054	-1.076668
C	5.661408	-1.194359	-0.016872	C	-5.827324	-1.279027	-1.030099
C	5.457975	0.094034	-0.532240	C	-6.003224	-0.728080	0.250802
C	4.595223	-2.082362	0.141049	C	-4.560334	-1.624707	-1.496294
C	3.330490	-1.638415	-0.227559	C	-3.476024	-1.391327	-0.653182
C	3.126886	-0.354200	-0.737714	C	-3.650885	-0.842264	0.616486
C	4.183730	0.530420	-0.904693	C	-4.916437	-0.510579	1.095544
H	6.316669	0.766304	-0.646745	H	-7.014600	-0.473944	0.589647
H	4.007145	1.533226	-1.312444	H	-5.040919	-0.094893	2.102334
H	6.674420	-1.507119	0.262230	H	-6.704977	-1.444957	-1.666122
H	4.741234	-3.094110	0.537007	H	-4.409809	-2.067861	-2.487746
C	2.023951	-2.350870	-0.175195	C	-2.030678	-1.686571	-0.881804
C	1.678079	-0.134962	-1.086490	C	-2.337347	-0.761009	1.317152
O	1.236340	0.797191	-1.710300	O	-2.174731	-0.626577	2.507050
O	1.787971	-3.496916	0.151391	O	-1.564386	-2.331752	-1.786486
C	0.985997	-1.352134	-0.561409	C	-1.232815	-0.972575	0.252350
N	-0.257643	-1.816372	-0.668141	N	-0.038185	-1.594201	0.722911
C	-1.431902	-1.187108	-0.571587	C	1.183776	-1.025561	0.258575
C	-2.547562	-2.023626	-0.048046	C	1.965354	-1.782931	-0.858651
C	-2.021378	0.033564	-1.191327	C	2.290826	-0.841896	1.326247
C	-3.453174	0.033888	-0.716263	C	3.601493	-0.939750	0.621752
C	-3.765750	-1.166565	-0.075074	C	3.415017	-1.495875	-0.643572
C	-5.040221	-1.417647	0.417719	C	4.493085	-1.746679	-1.489537
C	-6.003812	-0.419158	0.251972	C	5.765481	-1.408311	-1.032447
C	-5.691571	0.784662	-0.394156	C	5.952921	-0.849982	0.243483
C	-4.408209	1.024438	-0.895127	C	4.872497	-0.617432	1.092530
H	-4.153594	1.956958	-1.413185	H	5.006308	-0.196654	2.096012
H	-7.021076	-0.578639	0.628219	H	6.638287	-1.586262	-1.671897
H	-6.470600	1.547344	-0.511409	H	6.968237	-0.602301	0.575276
H	-5.273470	-2.366937	0.913950	H	4.334156	-2.196443	-2.476686
O	-1.553419	0.820259	-1.979213	O	2.130705	-0.713885	2.517061
O	-2.423676	-3.165079	0.351021	O	1.489928	-2.462697	-1.732651
H	-0.325645	-2.795518	-0.329092	H	-0.056802	-2.362983	1.380529

**TS-NI -4'**, PCM = THF	**TS-5a-*endo***, PCM = THF
E_0 = -1432.943535	E_0 = -1853.206823
E (298 K) = -1432.917975	E (298 K) = -1853.180696
H (298 K) = -1432.917031	H (298 K) = -1853.179752
G (298 K) = -1432.999297	G (298 K) = -1853.263107
Imaginary frequencies = 1 (-363 cm^{-1})	Imaginary frequencies = 1 (-323 cm^{-1})

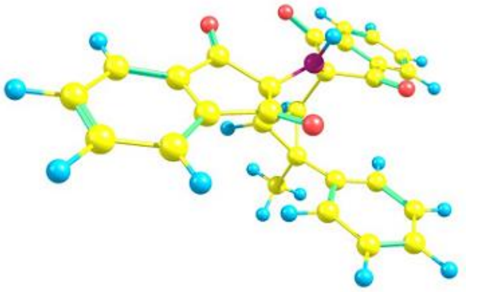

Cartesian coordinates:

C	-0.759948	-0.309600	1.141420
C	0.763961	-0.308894	1.126626
C	0.003498	0.951968	1.477602
H	-1.299102	-0.791482	1.966574
H	1.321975	-0.789896	1.939724
C	-0.007994	2.237274	0.697865
C	0.016859	1.241972	2.978335
H	-0.876951	1.827970	3.258011
H	0.912381	1.833040	3.241311
H	0.025060	0.307819	3.568314
C	-1.216667	2.889988	0.438891
C	-1.231039	4.159684	-0.136227
C	-0.029880	4.803503	-0.435949
C	1.182303	4.179700	-0.137471
C	1.189588	2.910169	0.437592
H	2.143194	2.431877	0.698125
H	-2.161636	2.396631	0.701556
H	-2.188042	4.652393	-0.346423
H	-0.038345	5.801796	-0.890270
H	2.130698	4.688247	-0.348955
C	-5.987578	-0.663255	-0.156108
C	-5.780470	-1.973419	0.307948
C	-4.918896	0.133063	-0.564184
C	-3.638813	-0.409014	-0.473258
C	-3.433025	-1.710227	-0.014305
C	-4.499163	-2.517187	0.376838
H	-6.644658	-2.576260	0.611365
H	-4.324028	-3.542945	0.722134
H	-7.009351	-0.268359	-0.204827
H	-5.069515	1.150325	-0.944487
C	-2.334072	0.180860	-0.889896
C	-1.977895	-2.042386	-0.068516
O	-1.485902	-3.134823	0.061244
O	-2.174393	1.100231	-1.655302
C	-1.214612	-0.695118	-0.281053
N	-0.017216	-0.686876	-1.047549
C	1.194890	-0.680389	-0.306852
C	2.291173	0.226715	-0.912909
C	1.991628	-2.013540	-0.138045
C	3.434625	-1.637426	-0.039499
C	3.607576	-0.323817	-0.477852
C	4.870781	0.260451	-0.535961
C	5.956307	-0.505733	-0.114509

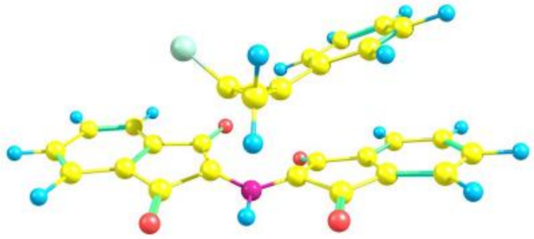

Cartesian coordinates:

C	5.358432	-1.286081	-0.264136
C	5.171156	-0.331696	-1.273712
C	4.272946	-1.964785	0.295221
C	3.006120	-1.652413	-0.182626
C	2.820250	-0.698531	-1.187038
C	3.894012	-0.029067	-1.754479
H	6.043990	0.184581	-1.691138
H	3.731778	0.718644	-2.540093
H	6.373831	-1.502416	0.088415
H	4.406074	-2.714804	1.083696
C	1.677010	-2.171991	0.239070
C	1.359081	-0.548698	-1.515973
O	0.905764	0.023694	-2.479825
O	1.409800	-3.056357	1.032785
C	0.667371	-1.349589	-0.470515
N	-0.605506	-1.702700	-0.282602
C	-1.720474	-0.980715	-0.326512
C	-2.927675	-1.629773	0.252735
C	-2.133809	0.250400	-1.061100
C	-3.625328	0.308437	-0.875683
C	-4.081126	-0.758062	-0.096279
C	-5.422332	-0.884953	0.246238
C	-6.303351	0.090700	-0.224453
C	-5.845673	1.161426	-1.006309
C	-4.494504	1.287616	-1.337480
H	-4.119801	2.124289	-1.938714
H	-7.369666	0.022850	0.020829
H	-6.563028	1.912232	-1.358289
H	-5.766469	-1.723531	0.862586
O	-1.460903	1.092925	-1.608666
O	-2.906172	-2.665794	0.888024
H	-0.712945	-2.563711	0.285526
H	-0.277993	-1.501152	2.138275
C	-0.152666	-0.413466	2.305490
H	2.892047	0.288674	2.353998
C	0.446848	0.439730	1.209424
C	2.814485	1.087590	1.604795
C	-0.877780	0.529054	1.411703
H	4.855033	1.751405	1.847676
C	1.601930	1.282203	0.930330
C	3.907037	1.902939	1.317154
C	1.494469	2.283747	-0.047142
C	3.799095	2.901890	0.347062
Cl	-2.023837	1.801002	1.599109
H	0.545382	2.396775	-0.589042

C	5.782588	-1.828012	0.327860
C	4.518794	-2.414927	0.361252
H	4.371707	-3.451131	0.687935
H	6.965349	-0.077113	-0.136007
H	6.659499	-2.406466	0.642092
H	4.996178	1.286713	-0.901031
O	1.533974	-3.127271	-0.093573
O	2.111492	1.137579	-1.683624
H	-0.027254	-0.808886	-2.051889
C	2.594131	3.088344	-0.335656
H	4.662953	3.537451	0.117171
H	2.511718	3.868279	-1.102211
H	0.045036	-0.138977	3.355357

TS-5a-*exo*, PCM = THF

E_0 = -1853.203145

E (298 K) = -1853.176979

H (298 K) = -1853.176035

G (298 K) = -1853.259376

Imaginary frequencies = 1 (-315 cm^{-1})

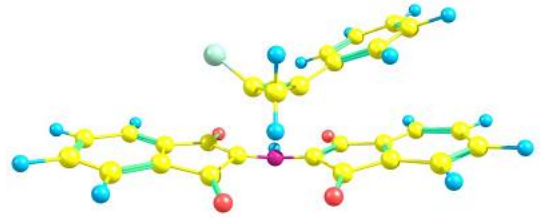

Cartesian coordinates:

C	-18.962791	3.894757	2.151659
C	-18.746329	2.516511	2.294922
C	-17.913297	4.758441	1.831909
C	-16.651927	4.199359	1.665223
C	-16.434655	2.827736	1.810885
C	-17.475296	1.962927	2.122265
H	-19.592003	1.864950	2.545029
H	-17.292444	0.887025	2.226162
H	-19.973092	4.296498	2.292082
H	-18.068653	5.837203	1.715149
C	-15.360030	4.855577	1.334300
C	-14.993677	2.475658	1.559622
O	-14.551886	1.353124	1.469379
O	-15.133280	6.016543	1.062020
C	-14.311423	3.792753	1.416837
N	-13.096928	4.187166	1.035689
C	-11.871524	3.738465	1.293650
C	-10.797867	4.764005	1.156309
C	-11.240927	2.414348	1.529769
C	-9.780920	2.731868	1.721022
C	-9.523780	4.087979	1.508532
C	-8.253667	4.624731	1.671177
C	-7.230942	3.752510	2.052084
C	-7.484777	2.389183	2.257015
C	-8.767951	1.858947	2.092333
H	-8.981331	0.795905	2.255315
H	-6.213755	4.136390	2.193546
H	-6.660222	1.729896	2.553621
H	-8.072770	5.694485	1.512671
O	-11.725075	1.303911	1.568307

O	-10.997064	5.924591	0.855875	
H	-13.082564	5.204218	0.817336	
H	-13.178547	1.616532	3.341860	
C	-13.122178	2.427271	4.087528	
H	-10.172537	2.659402	4.880225	
C	-12.376802	3.703551	3.759951	
C	-10.131505	3.753847	4.803719	
C	-13.721250	3.757364	3.740777	
H	-8.165853	3.914492	5.683168	
C	-11.218290	4.441663	4.245479	
C	-9.013134	4.457050	5.246333	
C	-11.171127	5.839743	4.128031	
C	-8.965688	5.846741	5.120038	
Cl	-14.890952	4.810445	4.464226	
H	-12.020727	6.371931	3.679032	
C	-10.044864	6.535612	4.559577	
H	-8.080723	6.398598	5.459256	
H	-10.008582	7.627029	4.460069	
H	-13.111290	2.098020	5.142205	

References

1. Moore, S.; Stein, W. H. *J. Biol. Chem.* **1948**, *176*, 367–388.

2. Wigfield, D. C.; Buchanan, G. W.; Croteau, S. M. *Can. J. Chem.* **1980**, *58,* 201–205.

3. Breslow, D.; Dowd, P. *J. Am. Chem. Soc.* **1963**, *85*, 2729–2735.

4. Longone, D. T.; Stehouwer, D. M. *Tetrahedron Lett.* **1970**, *11*, 1017–1019.

5. Padwa, A.; Blacklock, T. J.; Getman, D.; Hatanaka, N.; Loza, R. *J. Org. Chem.* **1978**, *43*, 1481–1492.

6. Gilbertson, R. D.; Weakley, T. J. R.; Haley, M. M. *J. Org. Chem.* **2000**, 65, 1422–1430.

7. White, E. H.; Winter, R. E. K.; Graeve, R.; Zirngibl, U.; Friend, E. W.; Maskill, H.; Mende, U.; Kreiling, G.; Reisenauer, H. P.; Maier, G. *Chem. Ber.* **1981**, *114*, 3906–3915.

8. Jones, W. M.; Stowe, M. E.; Wells, Jr., E. E. *J. Am. Chem. Soc.* **1968**, *90*, 1849–1859.

9. Rubin, M.; Gevorgyan, V. *Synthesis* **2004**, 796–800.

10. Alnasleh, B. K.; Sherrill, W. M.; Rubin, M. *Org. Lett.* **2008**, *10*, 3231–3234.

11. Henseling, K.-O.; Weyerstahl, P. *Chem. Ber.* **1975**, *108*, 2803–2808.

12. Lin, H.-C.; Tsai, R.-T.; Wu, H.-P.; Lee, H.-Y; Lee, G.-A. *Tetrahedron* **2016**, *72*, 184–191.

13. Lee, G.-A.; Chang, C.-Y. *Tetrahedron Lett.* **1998**, *39*, 3013−3016.

14. Binger, P.; Wedemann, P.; Goddard, R.; Brinker, U. H. *J. Org. Chem.* **1996**, *61*, 6462−6464.

15. Peveratti, R.; Truhlar, D. G. *J. Phys. Chem. Lett.* **2011**, *2*, 2810−2817.

16. Dunning, T. H. *J. Chem. Phys.* **1989**, *90*, 1007−1023.

17. Cossi, M.; Rega, N.; Scalmani, G.; Barone, V. *J. Comput. Chem.* **2003**, *24*, 669−681.

18. Schlegel, H. B. *J. Comput. Chem.* **1982**, *3*, 214−218.

19. Fukui, K. J. *J. Phys. Chem.* **1970**, *74*, 4161−4163.

20. Frisch, M. J.; Trucks, G.W.; Schlegel, H. B.; Scuseria, G. E.; Robb, M. A.; Cheeseman, J. R.; Scalmani, G.; Barone, V.; Mennucci, B.; Petersson, G. A.; Nakatsuji, H.; Caricato, M.; Li, X.; Hratchian, H. P.; Izmaylov, A. F.; Bloino, J.; Zheng, G.; Sonnenberg, J. L.; Hada, M.; Ehara, M.; Toyota, K.; Fukuda, R.; Hasegawa, J.; Ishida, M.; Nakajima, T.; Honda, Y.; Kitao, O.; Nakai, H.; Vreven, T.; Montgomery, J. A.; Peralta, J. E.; Ogliaro, F.; Bearpark, M.; Heyd, J. J.; Brothers, E.;

Kudin, K. N.; Staroverov, V. N.; Kobayashi, R.; Normand, J.; Raghavachari, K.; Rendell, A.; Burant, J. C.; Iyengar, S. S.; Tomasi, J.; Cossi, M.; Rega, N.; Millam, N. J.; Klene, M.; Knox, J. E.; Cross, J. B.; Bakken, V.; Adamo, C.; Jaramillo, J.; Gomperts, R.; Stratmann, R. E.; Yazyev, O.; Austin, A. J.; Cammi, R.; Pomelli, C.; Ochterski, J. W.; Martin, R. L.; Morokuma, K.; Zakrzewski, V. G.; Voth, G. A.; Salvador, P.; Dannenberg, J. J.; Dapprich, S.; Daniels, A. D.; Farkas, Ő.; Foresman, J. B.; Ortiz, J. V.; Cioslowski, J.; Fox, D. J. Gaussian 09, Revision C.01; Gaussian: Wallingford CT, **2013**.
